# Connecting data and expertise: a new alliance for biodiversity knowledge

**DOI:** 10.3897/BDJ.7.e33679

**Published:** 2019-03-08

**Authors:** Donald Hobern, Brigitte Baptiste, Kyle Copas, Robert Guralnick, Andrea Hahn, Edwin van Huis, Eun-Shik Kim, Melodie McGeoch, Isayvani Naicker, Laetitia Navarro, Daniel Noesgaard, Michelle Price, Andrew Rodrigues, Dmitry Schigel, Carolyn A. Sheffield, John Wieczorek

**Affiliations:** 1 Global Biodiversity Information Facility Secretariat, Copenhagen, Denmark Global Biodiversity Information Facility Secretariat Copenhagen Denmark; 2 Instituto de Investigación de Recursos Biológicos Alexander von Humboldt, Bogotá, Colombia Instituto de Investigación de Recursos Biológicos Alexander von Humboldt Bogotá Colombia; 3 Vertnet, Florida, United States of America Vertnet Florida United States of America; 4 University of Colorado, Boulder; University of Colorado Museum of Natural History, Boulder, United States of America University of Colorado, Boulder; University of Colorado Museum of Natural History Boulder United States of America; 5 Univ. of Florida, Gainesville, United States of America Univ. of Florida Gainesville United States of America; 6 Naturalis, Amsterdam, Netherlands Naturalis Amsterdam Netherlands; 7 Kookmin University, Seoul, South Korea Kookmin University Seoul South Korea; 8 Monash University, Clayton, Australia Monash University Clayton Australia; 9 African Academy of Sciences, Nairobi, Kenya African Academy of Sciences Nairobi Kenya; 10 German Centre for Integrative Biodiversity Research, Leipzig, Germany German Centre for Integrative Biodiversity Research Leipzig Germany; 11 Conservatoire et Jardin botaniques de la Ville de Genève, Geneva, Switzerland Conservatoire et Jardin botaniques de la Ville de Genève Geneva Switzerland; 12 Smithsonian Libraries/Biodiversity Heritage Library, Washington, DC, United States of America Smithsonian Libraries/Biodiversity Heritage Library Washington, DC United States of America; 13 VertNet, Bariloche, Argentina VertNet Bariloche Argentina; 14 Museum of Vertebrate Zoology, University of California, Berkeley, United States of America Museum of Vertebrate Zoology, University of California Berkeley United States of America

**Keywords:** Biodiversity, biodiversity data, biodiversity informatics, GBIC2, alliance, collaboration, data quality, sustainability, research infrastructure, open science, open data, investment

## Abstract

There has been major progress over the last two decades in digitising historical knowledge of biodiversity and in making biodiversity data freely and openly accessible. Interlocking efforts bring together international partnerships and networks, national, regional and institutional projects and investments and countless individual contributors, spanning diverse biological and environmental research domains, government agencies and non-governmental organisations, citizen science and commercial enterprise. However, current efforts remain inefficient and inadequate to address the global need for accurate data on the world's species and on changing patterns and trends in biodiversity. Significant challenges include imbalances in regional engagement in biodiversity informatics activity, uneven progress in data mobilisation and sharing, the lack of stable persistent identifiers for data records, redundant and incompatible processes for cleaning and interpreting data and the absence of functional mechanisms for knowledgeable experts to curate and improve data.

Recognising the need for greater alignment between efforts at all scales, the Global Biodiversity Information Facility (GBIF) convened the second Global Biodiversity Informatics Conference (GBIC2) in July 2018 to propose a coordination mechanism for developing shared roadmaps for biodiversity informatics. GBIC2 attendees reached consensus on the need for a global alliance for biodiversity knowledge, learning from examples such as the Global Alliance for Genomics and Health (GA4GH) and the open software communities under the Apache Software Foundation. These initiatives provide models for multiple stakeholders with decentralised funding and independent governance to combine resources and develop sustainable solutions that address common needs.

This paper summarises the GBIC2 discussions and presents a set of 23 complementary ambitions to be addressed by the global community in the context of the proposed alliance. The authors call on all who are responsible for describing and monitoring natural systems, all who depend on biodiversity data for research, policy or sustainable environmental management and all who are involved in developing biodiversity informatics solutions to register interest at https://biodiversityinformatics.org/ and to participate in the next steps to establishing a collaborative alliance.

The supplementary materials include brochures in a number of languages (English, Arabic, Spanish, Basque, French, Japanese, Dutch, Portuguese, Russian, Traditional Chinese and Simplified Chinese). These summarise the need for an *alliance for biodiversity knowledge* and call for collaboration in its establishment.

## Background

Biodiversity, the variation within species, between species and of ecosystems*^1^, is essential for the well-being of humanity and equilibrium of the planet. Sustainable use of biodiversity, including management of human impacts on biodiversity, is an increasingly important global challenge. The importance of biodiversity is reflected in the Aichi Biodiversity Targets of the Strategic Plan for Biodiversity 2011-2020 (www.cbd.int/sp/targets), the United Nations Sustainable Development Goals (http://sustainabledevelopment.un.org) and the GEO societal benefit areas (http://earthobservations.org/sbas.php). These global plans highlight the need for better integrated information on biodiversity patterns and change. Aichi Target 19 specifically focuses on knowledge access: “By 2020, knowledge, the science base and technologies relating to biodiversity, its values, functioning, status and trends, and the consequences of its loss, are improved, widely shared and transferred, and applied”.

Addressing this challenge is difficult because our fundamental understanding of the complexity and dynamics of biodiversity remains inadequate. Over the past quarter of a millennium, taxonomists have described around 2 million distinct species. Estimates of the actual number of extant species vary (Chapman 2009), in particular because of uncertainties around the scale of microbial biodiversity, but a majority of the world’s species are still undescribed, even to a basic level. Our understanding of their evolutionary history, community interactions and functional biology lags even further behind. Sustainable management of the landscape depends on monitoring, understanding, predicting and responding to changes in the spatiotemporal organisation of a system within which even the most basic units have not been catalogued and characterised. This knowledge gap affects our ability to preserve a significant portion of existing biodiversity*^2^, as well as our prospects for maintaining food security, human health and fundamental environmental processes that provide clean air and fresh water.

For centuries, biologists have been collecting specimens and recording observations that offer the historical context for modern scientific biodiversity-based investigations and predictive modelling. Many complementary sources of observations and measurements provide contemporary perspectives, including, amongst others, ecological research and field-based monitoring, citizen science and local community initiatives, molecular studies, automated cameras and sensors and satellite imagery.

Over the last twenty years, a steady growth in informatics investments, on many different scales, has served to make biodiversity information from all of these sources accessible online. During 2018, data published and aggregated through the Global Biodiversity Information Facility (GBIF) surpassed one billion records, each serving as evidence for the occurrence of a species at a given time and place. Associated networks, such as the Ocean Biogeographic Information System (OBIS) and national biodiversity information facilities, also each provide access to significant volumes of occurrence data. The 2018 edition of the Catalogue of Life Annual Checklist includes taxonomic information on 1,803,488 living and extinct species (around 75% of known species). The Biodiversity Heritage Library now includes more than 55 million pages of scanned biodiversity literature. The Barcode of Life Data System includes 6,293,000 barcode sequences representing 280,000 species. The recently established Global Genome Biodiversity Network*^3^(www.ggbn.org/ggbn_portal) provides access to more than 2 million DNA and tissue samples of 45,000 species. The Encyclopedia of Life Traitbank*^4^(https://eol.org/docs/what-is-eol/traitbank) holds structured data on 11 million species traits. Important resources such as the World Database on Protected Areas*^5^, the IUCN Red List of Threatened Species and the Global Register of Introduced and Invasive Species (GRIIS) organise and maintain essential information for conservation and management of biodiversity. Hundreds of other projects and organisations, working at different scales, are actively databasing or aggregating information on the world’s species.

These efforts have made significant progress in facilitating open access to biodiversity data, increasing scientific understanding and providing relevant information for policy on the environment, conservation and sustainability. Improved modelling of species distributions at all scales supports evidence-based planning and decision-making. However, as acknowledged on multiple occasions by major biodiversity informatics initiatives*^6^, ^7^, ^8^, ^9^, existing systems remain poorly interconnected and do not yet deliver a truly global infrastructure for biodiversity knowledge access. In addition, many taxonomic and geographic gaps remain in available data. Even when data have been shared online in digital formats, they are not always integrated into existing data networks using common standards and practices.

A further concern is the disconnection between these systems and the communities which have the necessary expertise to validate, curate and improve data from diverse sources. This leads to quality issues with implications for subsequent analysis and use. The causes of this disconnection include both sociological (Franz and Sterner 2018) and technical factors (Mesibov 2018). Novel approaches are required to enable researchers to engage effectively and efficiently with vast volumes of complex data, to maximise the information that can be contributed through simple curatorial actions and to give full and appropriate credit for efforts to improve digital knowledge.

Stakeholders have been able to develop a common vision for aligning their activities, as described in the 2012 *Global Biodiversity Informatics Outlook (GBIO)* (see Hobern et al. 2012) and the 2013 *Decadal View of Biodiversity Informatics* (see Hardisty et al. 2013). Nevertheless, the complexity of the domain covered by biodiversity informatics, combined with the number and diversity of relevant projects and initiatives, mostly reliant on short-term funding, has limited progress in making such alignment.

The Senckenberg Gesellschaft für Naturforschung hosted a workshop on *Exploring Synergies and Sustainability for Biodiversity Information Systems* in March 2017. Attendees representing global data infrastructures, national data centres and major research institutions agreed that the global community needed to develop a shared mechanism for planning, delivering and sustaining a linked and open global biodiversity data infrastructure. This workshop did not determine what form such a mechanism should take or whether the desired outcomes would require a sustainable long-term, persistent decision-making body or could be achieved through shorter time-bound collaboration. Accordingly, the Global Biodiversity Information Facility (GBIF), as part of its 2018 Work Programme *^10^, organised a follow-up workshop to explore how such a mechanism might operate. This event, GBIC2: the 2nd Global Biodiversity Informatics Conference*^11^ (https://biodiversityinformatics.org), took place in Copenhagen from 24 to 27 July 2018. The name refers back to the first GBIC conference, which delivered the GBIO document in 2012. GBIC2 hosted 103 representatives from key global, regional, national and subnational networks, agencies, organisations and institutions. Participants are listed in *Annex B - List of GBIC2 Attendees* (*Suppl. material 2*).

## Structure of GBIC2 discussions

GBIC2 began with an overview of the complex landscape of stakeholders that contribute or make use of biodiversity information. These stakeholders included a broad range of scientific communities, politicians, non-governmental organisations, commercial entities and public stakeholders, including indigenous and local communities.

**Figure 1.**
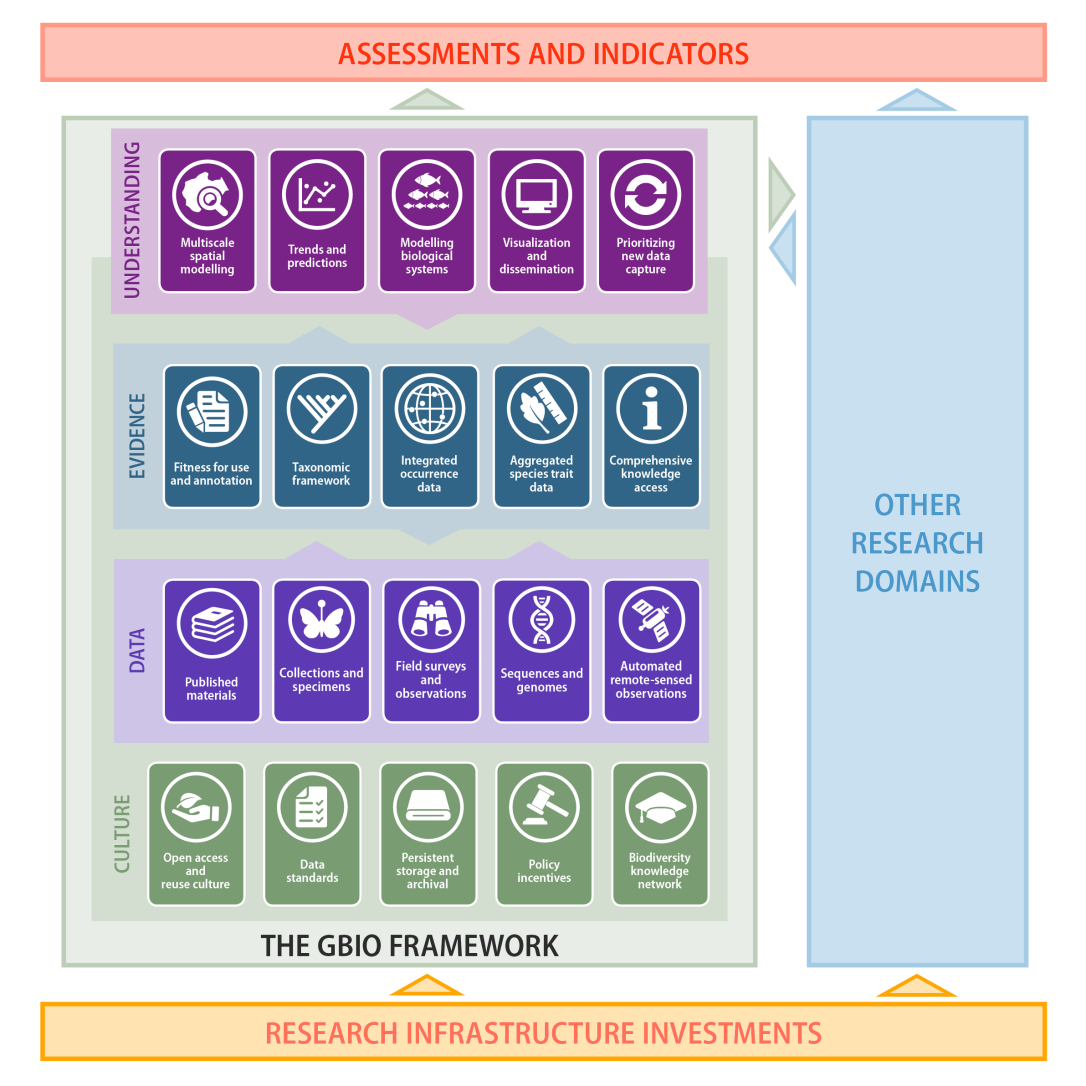
The GBIO Framework (Hobern et al. 2012) identified 20 components as essential elements of biodiversity informatics and organised as four layers: Culture, Data, Evidence and Understanding. The framework offered a coordinated model for delivering the data products required to support biodiversity *assessments and indicators*. An organised approach of this kind also facilitated the bidirectional linkages necessary with *other research domains* (environment, climate, agriculture etc.), particularly to refine modelled representations of biodiversity. The GBIO Framework (and the efforts of other research domains) should benefit from and build on *research infrastructure investments* that are in place or planned in many countries and regions.

The earlier GBIO framework (Fig. 1) offered a structured, high-level vision for mobilising, managing and delivering biodiversity data to benefit and support all of these groups. It described four mutually supportive tiers, each with five constituent components:

- The **Culture** tier addresses the open data and open science context required to enable effective collaborative information sharing, with the following components:
  - Open access and reuse culture
  - Data standards
  - Persistent storage and archives
  - Policy incentives
  - Biodiversity knowledge network
- The **Data** tier focuses on digital access to well-formed streams of data from all relevant sources of biodiversity observations and measurements, with the following components:
  - Published materials
  - Collections and specimens
  - Field surveys and observations
  - Sequences and genomes
  - Automated, remote-sensed observations
- The **Evidence** tier focuses on organising these disparate streams into accessible, integrated information resources, with the following components:
  - Fitness-for-use and annotations
  - Taxonomic and phylogenetic frameworks
  - Integrated occurrence data
  - Aggregated species trait data
  - Comprehensive knowledge access
- The **Understanding** tier focuses on building modelled representations of biodiversity patterns and properties, based on all available evidence, with the following components:
  - Multiscale species modelling
  - Trends and predictions
  - Modelling biological systems
  - Visualisation and dissemination
  - Prioritising new data capture

Each of the twenty components in this framework encompasses the activities of many existing organisations, agencies or projects as well as individual researchers.

The challenge presented for the GBIC2 attendees was to propose a collaborative approach for the global community for planning and agreeing on an optimal set of new or improved policies, data standards, processes, governance arrangements, software tools, informatics infrastructure investments and research programmes, with sufficient clarity to deliver an interoperable global infrastructure of the kind envisioned by the GBIO framework.

Four parallel working groups reviewed different components from the GBIO framework, each selected to capture a broad range of different challenges and opportunities:

- Biodiversity knowledge network (Culture)
- Published materials (Data)
- Integrated occurrence data (Evidence)
- Trends and predictions (Understanding)

Each of these working groups sought to identify major sociological and technical issues limiting the effectiveness of current solutions in the area concerned. They then considered possible responses by the global community to resolve these issues and accelerate delivery as part of a broader biodiversity informatics landscape. The outputs from these four working groups are summarised in *Annex A - Outputs from GBIC2 Working Groups* (Suppl. material 1).

Plenary presentations helped guide discussion of governance models that could enable such coordination:

- Donald Hobern: GBIC2 - Realising the Vision*^12^and GBIC2 - Closing Session*^13^
- Robert Hanisch: International Collaboration for Open Data and Open Science*^14^, including examples from the International Virtual Observatory Alliance (IVOA) and Research Data Alliance (RDA)
- Maria Uhle: A Funders’ Perspective*^15^ , including examples from the work of the Belmont Forum
- Jerry Lanfear: ELIXIR: the European ELIXIR consortium and delivery of core data services*^16^, presenting ELIXIR as an example of an intergovernmental organisation working to coordinate national resources to form a single infrastructure
- Ismaêl Mejía: The Apache Way and collaborative development*^17^, presenting the model of the Apache Software Foundation for community-based incubation of collaborative software development projects

## A common ambition for biodiversity informatics

The attendees at GBIC2 reached consensus*^18^ on a multifaceted vision for interconnected solutions that address existing limitations. This vision represents a large number of shared ambitions:

*Support for science and evidence-based planning*

1. To deliver biodiversity knowledge and understanding in forms which support critical research requirements and enable biodiversity to be correctly measured and assessed for societal goals*^19^
2. To serve as the foundation for basic research in biodiversity and information science that serves human understanding of the functioning and state of natural systems
3. To provide a platform for continuous growth in understanding of biodiversity by preserving, building on and improving existing knowledge

*Support for open data and open science*

1. To remove barriers to free and open sharing of data and to the adoption of FAIR data principles (Wilkinson et al. 2016) for biodiversity data
2. To describe all data resources with rich metadata that supports present and future reuse
3. To ensure all data resources are preserved in stable and persistent trusted repositories
4. To enable collaborative curation, annotation and improvement of all data by any relevant experts and expert communities*^20^
5. To enable all contributors of knowledge or expertise to have their contributions fully recorded, acknowledged and credited
6. To track the provenance and attribution of all sources of information

*Support for highly-connected biodiversity data*

1. To mobilise structured digital representations of historical data sources, including museum collections and literature
2. To ensure that all new observations and measurements are accessible in structured digital representations as rapidly as possible after capture
3. To enable the combination, querying and analysis of different classes of biodiversity information (distribution, traits, genes etc.) as an interconnected whole
4. To work with other research communities and infrastructures to achieve interoperability with earth observations, social science data and other resources

*Support for international collaboration*

1. To address capacity needs around biodiversity informatics in all regions and in all sectors
2. To secure funding to maintain services and components that the community recognises as critical elements within a distributed knowledge infrastructure*^21^
3. To develop flexible, collaborative approaches to designing, building and sustaining all components of this distributed knowledge infrastructure
4. To enable stakeholders in every country and region to adopt and benefit from advances in infrastructure, tools, services, practices and capacity
5. To enable full participation of and collaboration with all stakeholder groups in all regions at all stages, from data generation to analysis and application
6. To enable data repatriation that supports science and policy-making in all countries and regions
7. To ensure effective access to and use of data at every scale—global, regional, national and local
8. To acknowledge and support the role of regional, national and local investments as critical and effective components within a global solution
9. To overcome barriers to data sharing or use arising from language or culture
10. To support the practical implementation of international agreements with reference to access and benefit sharing

Although the stakeholders at GBIC2 all represented organisations that focus on a subset of the listed ambitions, each recognised the role that their work programmes can play as components within this larger shared vision. No single stakeholder has responsibility for coordination and delivery of this integrated vision, nor is any stakeholder positioned to address these challenges for all communities and at all scales.

The potential strategies and investments identified by the four working groups to address current impediments in biodiversity informatics reinforced this vision. Many of the suggested solutions depended fundamentally on all stakeholders adopting a single shared process or implementation. The majority of the rest of the suggestions would deliver significant multiplier effects as stakeholders converged on more standardised approaches. The robust and sustainable solutions needed to address many well-understood challenges are unimaginable without an open and inclusive approach that maximises cooperation.

Accordingly, GBIC2 attendees re-asserted the need for a coordination mechanism that helps all parties align their missions and work programmes in a complementary fashion.

## Models for coordination

Many diverse organisations and institutions, including governments, academia, industry and public stakeholders, have an interest in some aspects of biodiversity knowledge management. In particular, independent national investments form a major and significant part of overall funded activity and frequently deliver tools, standards and services later adopted in other countries and regions. Examples include the services and tools provided by the Flanders Marine Institute (VLIZ) to support the World Register of Marine Species, the Living Atlases software originally developed for the Atlas of Living Australia and tools built by the Humboldt Institute in Colombia to support GEO BON. As a result, any coordination mechanism must provide the flexibility both to accommodate and to benefit from this diversity, rather than seeking to implement a prescriptive programme of planned deliverables.

The design of any viable model for coordinating activity should therefore support:

- open discussion of requirements and open communication and participation by all stakeholders
- development of a shared vision and roadmap for increasing alignment
- collaborative approaches to design, fund, implement and sustain infrastructure components and tools required by multiple stakeholders

It is possible that some aspects of this coordination could be handled within the mission of existing international organisations.

The final report of the OECD Megascience Forum Working Group on Biological Informatics*^22^ in 1999 proposed the establishment of a Global Biodiversity Informatics Facility, with a suggested scope that included most or all of the vision described above and included within the GBIO Framework. However, upon its establishment in 2002, the resources available to make this ‘GBIF’ operational reduced its initial scope to focus on the informatics and capacity enhancement needed to support the digitisation of natural history collections and the development of a taxonomic framework. Other proposed work areas were deferred and have since been adopted within the missions of, for example, the Encyclopedia of Life (corresponding to the OECD proposal for a “SpeciesBank”) and of the Biodiversity Heritage Library (corresponding to the proposed inclusion of “digital biodiversity literature resources”). Today’s biodiversity informatics landscape is much more complex than in 1999, with many international initiatives that have taken up challenges originally intended for GBIF. Given these developments and the wide range of other stakeholders with an interest in high-quality access to biodiversity data, the role of an international organisation, such as GBIF, in enabling and facilitating global coordination of players in this ever-changing ecosystem, remains essential.

The Intergovernmental Science-Policy Platform on Biodiversity and Ecosystem Services (IPBES) and the Convention on Biological Diversity (CBD) may provide relevant settings in which further necessary discussions could take place, though these bodies are less closely involved in the technical development of data infrastructures. GEO BON is also an important international network, particularly for the earth-observing and monitoring aspects.

As a result, the GBIC2 attendees agreed to explore the establishment of a lightweight alliance, building on lessons learned from different collaborative models, including open-source software communities such as the Apache Software Foundation (ASF) and open science partnerships such as the Global Alliance for Genomics and Health (GA4GH). GBIC2 tasked GBIF with facilitating the exploration and establishment phase for a global alliance to transform our understanding of biodiversity by connecting all efforts to observe, measure and model the living planet.

By facilitating open discussion between interested and aligned parties, this alliance will seek to refine the GBIO framework and construct a shared roadmap for interoperable infrastructure components, as with the GA4GH Strategic Roadmap*^23^. A related goal for this alliance would be the documentation of agreed standards and best practices and a catalogue of services identified by the community as critical and requiring sustained support, along the lines of the ELIXIR Core Data Resources*^24^. Perhaps most significantly, an alliance could function as an incubator for designing and implementing important but missing components of the distributed infrastructure, drawing from the project model supported by the Apache Software Foundation*^25^.

Such an alliance should be expansive enough to include representation and perspectives outside those actively delivering data or building informatics infrastructure. In particular, it should facilitate closer collaboration with practitioners working in conservation, environmental management and sustainable development.

## Towards an alliance for biodiversity knowledge

GBIC2 attendees identified five initial steps to advance the concept of this proposed *alliance for biodiversity knowledge*. The GBIF Secretariat will provide administrative and technical support for this exploratory phase.

### Expand engagement

The goal following GBIC2 is to refine the concept and to develop a broad and inclusive community of stakeholders interested in mobilising, improving and using biodiversity information. The immediate priority is to communicate this goal and expand engagement and support for implementing an appropriate model.

This paper is therefore presented as an invitation to the global community to develop a new coordination and collaboration model and to work towards the multifaceted vision outlined above. We call below for institutions and individuals to indicate their support and to participate in online discussions around possible models for such an alliance. An alliance site has been established at https://biodiversityinformatics.org/ to organise supporting materials and to host discussions for the other steps outlined below. Contributions in languages other than English are welcomed. Recommendations arising from these discussions will be synthesized within a proposed Charter and Mission for the alliance. We urge readers to participate in this process and to contribute to developing a model that will benefit all parties.

### Evaluate models

Although GBIC2 discussions wholeheartedly supported the establishment of a lightweight alliance and identified relevant examples from other domains, more work is required to meet the needs of this complex and diverse stakeholder community. Review of other similar alliances, coalitions and consortia should guide and assist planning for longer term approaches.

GBIC2 attendees acknowledged the remarkable success of the Apache Software Forum in fostering healthy and productive international projects involving many organisations and individuals. The biodiversity informatics community might benefit from adoption of elements from ‘The Apache Way’, in particular its merit- or reputation-based model for individual contributors to participate within a community. These approaches could be relevant, not only to the development of components of a collaborative infrastructure, but also to cooperation around curation of major datasets. A major aspect of ASF’s success comes from the culture that they promote through their Code of Conduct*^26^. This focuses on aspects such as openness, respect, diversity, conciseness in communication and mutual dependence on a collaborative approach to problem-solving. Similar approaches are likely to be highly beneficial within the proposed *alliance for biodiversity knowledge*.

Important questions relating to the basis of membership remain open, such as whether both individuals and organisations should be eligible to become members. Both models may be valid, as exemplified by GA4GH, which represents an alliance of member organisations and ASF, which operates through participation of individuals, even when many of them represent organisations.

### Clarify scope and target outcomes

The GBIO framework and other reviews offer clear conceptual precedents for fitting disparate components together within an integrated biodiversity informatics infrastructure. The multifaceted vision outlined above shows the convergence of understanding amongst international stakeholders. Calls for the development of Essential Biodiversity Variables (Pereira et al. 2013), a Biodiversity Knowledge Graph (Page 2016) or a Global Virtual Natural History Collection (Balke et al. 2013) each represent different aspects or consequences of this core vision.

However, these perspectives are not adequate by themselves to establish the immediate and medium-term goals for this alliance. It is important to establish a set of significant but achievable use cases to guide thinking and prioritisation over a five- to ten-year period.

GBIC2 attendees therefore proposed a consultation with diverse stakeholders—including research groups, taxonomic facilities, the CBD, IPBES, FAO, conservation bodies and other user communities—to develop a set of defining questions or use cases against which to measure progress. These questions should address a range of research and societal needs with sufficient detail and precision to guide priorities for collaborative planning, development and implementation, while serving as milestones for improved collaborative delivery.

GBIF will work with representatives from GBIC2 and with other interested parties to consult widely in developing this set of defining questions online at https://biodiversityinformatics.org/.

### Map stakeholders

The stakeholder landscape is complex. Overlapping and changing missions, work programmes and responsibilities applying at different scales make it difficult to identify all parties with existing interests in addressing particular challenges—increasing the risk of inadvertent conflict or duplication of effort.

GBIC2 attendees therefore agreed on the importance of a network analysis to understand the roles and responsibilities of major organisations, particularly at global, regional and national scales, including major components in their work programmes and deliverables and dependencies.

This challenging problem could readily absorb significant resources, especially if the proposed alliance seeks to maintain an up-to-date information overview over time. It is critical, therefore, that the entities and information to be included in a stakeholder map are clearly and tightly scoped. This work will benefit from expertise within the social sciences in mapping and analysing the structure of community networks.

GBIF will coordinate initial discussion of possible models to capture appropriate landscape information. Successful understanding of stakeholder relationships will assist the proposed alliance to identify critical services that need to be created or sustained and indicate opportunities for better alignment or unification of services developed by different parties.

### Adopt proof-of-concept projects

Several existing collaborative activities are representative of the range of possible projects that an alliance might incubate. In order to raise visibility and to make progress in a more open and transparent way, GBIC2 participants and other interested parties are encouraged to propose existing activities as early proof-of-concept projects for the alliance model.

During the establishment phase of the alliance, governance processes will not be in place to support all aspects of such projects. However, adoption of practical examples will likely help develop the concept for and approaches to the alliance itself.

A transparent process is required for selecting even proof-of-concept projects. This should take into account the relevance of the project to a broad range of stakeholders, as well as the openness of the project for new partners to join and contribute to the plans and deliverables. The incubation process*^27^ used by the Apache Software Foundation and the criteria used by GA4GH to select Driver Projects*^28^ are useful models.

As an example of an existing collaboration, which might serve as a proof-of-concept project and which would benefit from increased exposure and openness, Catalogue of Life, GBIF, Encyclopedia of Life, Barcode of Life Data Systems and Biodiversity Heritage Library are currently working to develop a new collaborative model for building a shared taxonomic framework, under the project name, *Catalogue of Life Plus* (CoL+). This activity is currently funded by GBIF Netherlands and is seeking wider engagement with other bodies that are responsible for checklists or nomenclatural datasets. Increasing the transparency of this activity and involving other biodiversity informatics initiatives would allow CoL+ to engage more broadly with taxonomic authorities and maximise expertise contributed across different taxonomic groups. CoL+ could therefore serve as an early proof of concept for an alliance-led incubator project.

Additionally, there are growing efforts in several regions, particularly the NSF Advancing Digitization of Biodiversity Collections (ADBC) program*^29^ and the European Distributed Systems of Scientific Collections (DiSSCo)*^30^ consortium, to develop virtual natural history collections offering online access to rich digital representations of natural history specimens. These projects will drive development of new technologies and standards and need community models to ensure interoperability between the systems they deliver. The alliance will provide the necessary framework for cooperation and alignment of efforts.*^29^

Stakeholders are encouraged to identify other suitable collaborative projects, not just in the development of software or management of data, but also in other areas of activity, such as capacity enhancement and sustainability planning.

## Call to action

GBIF has allocated funds as part of its 2019 Work Programme for work on the initial steps identified above. The *SYNTHESYS*+ project, funded by the European Commission, includes additional funding for coordination activity and workshops. The *biodiversity_next*conference, which is planned as a major international forum for biodiversity informatics and which will take place in Leiden in October 2019, will serve as a venue for updates on progress.

Information on these activities and opportunities to participate in developing the alliance concept will be shared through an alliance website at https://biodiversityinformatics.org/. As far as possible, to maximise input from stakeholders in all regions, all discussions will be take place through open online discussion. A brochure for wider communication, *an alliance for biodiversity knowledge*, is included here (Suppl. material 3), along with translations of this brochure into Arabic (Suppl. material 4), Spanish (Suppl. material 5), Basque (Suppl. material 6), French (Suppl. material 7), Japanese (Suppl. material 8), Dutch (Suppl. material 9), Portuguese (Suppl. material 10), Russian (Suppl. material 11), Simplified Chinese (Suppl. material 12) and Traditional Chinese (Suppl. material 13).

We urge all stakeholders with an interest in the production, management and use of data on the world’s biodiversity to visit this site and indicate their interest and support for collaborating on development of an *alliance for biodiversity knowledge*. Discussion threads have also been opened to facilitate exploration of the initial steps identified above (Expand engagement; Evaluate models; Clarify scope and target outcomes; Map stakeholders; Adopt proof-of-concept projects). These discussions aim to define the parameters for workshops, white papers and online consultations starting in the coming months, so that, by late 2019, a model can be proposed for the operation of the alliance, including a framework for developing the shared roadmap for implementing infrastructure components and services.

## Supplementary Material

Supplementary material 1 Annex A - Outputs from GBIC2 Working Groups Data type: text document
Brief description: Outputs from GBIC2 Working Groups
File: oo_268553.pdf Donald Hobern, Brigitte Baptiste, Kyle Copas, Robert Guralnick, Andrea Hahn, Edwin van Huis, Eun-Shik Kim, Melodie McGeoch, Isayvani Naicker, Laetitia Navarro, Daniel Noesgaard, Michelle Price, Andrew Rodrigues, Dmitry Schigel, Carolyn Sheffield, John Wieczorek

Supplementary material 2 Annex B - List of GBIC2 Attendees Data type: text document
Brief description: List of GBIC2 Attendees
File: oo_269053.pdf Donald Hobern, Brigitte Baptiste, Kyle Copas, Robert Guralnick, Andrea Hahn, Edwin van Huis, Eun-Shik Kim, Melodie McGeoch, Isayvani Naicker, Laetitia Navarro, Daniel Noesgaard, Michelle Price, Andrew Rodrigues, Dmitry Schigel, Carolyn Sheffield, John Wieczorek

Supplementary material 3 Call for an alliance for biodiversity knowledge Data type: text document
Brief description: English PDF brochure presenting a call to participate in an alliance for biodiversity knowledge
File: oo_269025.pdf Donald Hobern, Brigitte Baptiste, Kyle Copas, Robert Guralnick, Andrea Hahn, Edwin van Huis, Eun-Shik Kim, Melodie McGeoch, Isayvani Naicker, Laetitia Navarro, Daniel Noesgaard, Michelle Price, Andrew Rodrigues, Dmitry Schigel, Carolyn Sheffield, John Wieczorek

Supplementary material 4 دعوة لتكوين تحالف لمعرفة التنوع البيولوجي Data type: text document
Brief description: Arabic PDF brochure presenting a call to participate in an alliance for biodiversity knowledge
File: oo_269026.pdf Donald Hobern, Brigitte Baptiste, Kyle Copas, Robert Guralnick, Andrea Hahn, Edwin van Huis, Eun-Shik Kim, Melodie McGeoch, Isayvani Naicker, Laetitia Navarro, Daniel Noesgaard, Michelle Price, Andrew Rodrigues, Dmitry Schigel, Carolyn Sheffield, John Wieczorek

Supplementary material 5 Convocatoria de una alianza para el conocimiento de la biodiversidad Data type: text document
Brief description: Spanish PDF brochure presenting a call to participate in an alliance for biodiversity knowledge
File: oo_269027.pdf Donald Hobern, Brigitte Baptiste, Kyle Copas, Robert Guralnick, Andrea Hahn, Edwin van Huis, Eun-Shik Kim, Melodie McGeoch, Isayvani Naicker, Laetitia Navarro, Daniel Noesgaard, Michelle Price, Andrew Rodrigues, Dmitry Schigel, Carolyn Sheffield, John Wieczorek

Supplementary material 6 Biodibertsitatea ezagutzeko aliantza baterako deialdia Data type: text document
Brief description: Basque PDF brochure presenting a call to participate in an alliance for biodiversity knowledge
File: oo_269028.pdf Donald Hobern, Brigitte Baptiste, Kyle Copas, Robert Guralnick, Andrea Hahn, Edwin van Huis, Eun-Shik Kim, Melodie McGeoch, Isayvani Naicker, Laetitia Navarro, Daniel Noesgaard, Michelle Price, Andrew Rodrigues, Dmitry Schigel, Carolyn Sheffield, John Wieczorek

Supplementary material 7 Appel à une alliance pour la connaissance sur la biodiversité Data type: text document
Brief description: French PDF brochure presenting a call to participate in an alliance for biodiversity knowledge
File: oo_269029.pdf Donald Hobern, Brigitte Baptiste, Kyle Copas, Robert Guralnick, Andrea Hahn, Edwin van Huis, Eun-Shik Kim, Melodie McGeoch, Isayvani Naicker, Laetitia Navarro, Daniel Noesgaard, Michelle Price, Andrew Rodrigues, Dmitry Schigel, Carolyn Sheffield, John Wieczorek

Supplementary material 8 生物多様性の知識のための連携協力の要請 Data type: text document
Brief description: Japanese PDF brochure presenting a call to participate in an alliance for biodiversity knowledge
File: oo_269725.pdf Donald Hobern, Brigitte Baptiste, Kyle Copas, Robert Guralnick, Andrea Hahn, Edwin van Huis, Eun-Shik Kim, Melodie McGeoch, Isayvani Naicker, Laetitia Navarro, Daniel Noesgaard, Michelle Price, Andrew Rodrigues, Dmitry Schigel, Carolyn Sheffield, John Wieczorek

Supplementary material 9 Call: Een alliantie voor kennis over biodiversiteit Data type: text document
Brief description: Dutch PDF brochure presenting a call to participate in an alliance for biodiversity knowledge
File: oo_269031.pdf Donald Hobern, Brigitte Baptiste, Kyle Copas, Robert Guralnick, Andrea Hahn, Edwin van Huis, Eun-Shik Kim, Melodie McGeoch, Isayvani Naicker, Laetitia Navarro, Daniel Noesgaard, Michelle Price, Andrew Rodrigues, Dmitry Schigel, Carolyn Sheffield, John Wieczorek

Supplementary material 10 Apelo a uma aliança para o conhecimento da biodiversidade Data type: text document
Brief description: Portuguese PDF brochure presenting a call to participate in an alliance for biodiversity knowledge
File: oo_269033.pdf Donald Hobern, Brigitte Baptiste, Kyle Copas, Robert Guralnick, Andrea Hahn, Edwin van Huis, Eun-Shik Kim, Melodie McGeoch, Isayvani Naicker, Laetitia Navarro, Daniel Noesgaard, Michelle Price, Andrew Rodrigues, Dmitry Schigel, Carolyn Sheffield, John Wieczorek

Supplementary material 11 Призыв к созданию альянса знаний по биоразнообразию Data type: text document
Brief description: Russian PDF brochure presenting a call to participate in an alliance for biodiversity knowledge
File: oo_269034.pdf Donald Hobern, Brigitte Baptiste, Kyle Copas, Robert Guralnick, Andrea Hahn, Edwin van Huis, Eun-Shik Kim, Melodie McGeoch, Isayvani Naicker, Laetitia Navarro, Daniel Noesgaard, Michelle Price, Andrew Rodrigues, Dmitry Schigel, Carolyn Sheffield, John Wieczorek

Supplementary material 12 对建立生物多样性知识联盟的呼吁 Data type: text document
Brief description: Simplified Chinese PDF brochure presenting a call to participate in an alliance for biodiversity knowledge
File: oo_269037.pdf Donald Hobern, Brigitte Baptiste, Kyle Copas, Robert Guralnick, Andrea Hahn, Edwin van Huis, Eun-Shik Kim, Melodie McGeoch, Isayvani Naicker, Laetitia Navarro, Daniel Noesgaard, Michelle Price, Andrew Rodrigues, Dmitry Schigel, Carolyn Sheffield, John Wieczorek

Supplementary material 13 呼籲成立生物多樣性知識聯盟 Data type: text document
Brief description: Traditional Chinese PDF brochure presenting a call to participate in an alliance for biodiversity knowledge
File: oo_269038.pdf Donald Hobern, Brigitte Baptiste, Kyle Copas, Robert Guralnick, Andrea Hahn, Edwin van Huis, Eun-Shik Kim, Melodie McGeoch, Isayvani Naicker, Laetitia Navarro, Daniel Noesgaard, Michelle Price, Andrew Rodrigues, Dmitry Schigel, Carolyn Sheffield, John Wieczorek

## Footnotes

*1
https://www.cbd.int/convention/articles/default.shtml?a=cbd-02

*2
http://www.pnas.org/content/early/2016/04/26/1521291113

*3
http://www.ggbn.org/ggbn_portal/

*4
https://content.eol.org/traitbank

*5
https://www.protectedplanet.net/c/world-database-on-protected-areas

*6
http://old.tdwg.org/fileadmin/documentation/outreach/OGC_TDWG_GBIF_presentation_Hobern.pdf

*7
http://wayback.archive-it.org/org-660/20140924185333/http://www.e-biosphere09.org/assets/files/workshop/Resolution.pdf

*8
https://cordis.europa.eu/result/rcn/164198_en.html

*9
http://www.globis-b.eu/files/intranet/Deliverables/Final/D4_3-Policy%20position%20final.pdf

*10
https://www.gbif.org/document/36j6HhbR4kOMY6oqcamEMk

*11
https://biodiversityinformatics.org

*12
https://doi.org/10.15468/doc.j9z1-ad72

*13
https://doi.org/10.15468/doc.2dz0-fk53

*14
https://doi.org/10.15468/78eg-pd83

*15
https://doi.org/10.15468/doc.pnd4-wq59

*16
https://doi.org/10.15468/bjrz-rf70

*17
https://doi.org/10.15468/doc.mbrj-gq93

*18 Consensus was reached through open discussion by all participants during closing plenary sessions of the GBIC2 workshop and confirmed through input, open review and feedback to this document by attendees.

*19 Societal goals will evolve over time and systems and services will need to evolve to meet new requirements.

*20 The term "expert" is intended to cover any contributor with relevant expertise and is not restricted to professional researchers.

*21 This "distributed global infrastructure" will necessarily depend on many underlying tools and services developed and maintained by many institutions and agencies. The community must establish processes to identify components that are important to the operation and sustainability of this overall infrastructure and to manage the lifecycle of such components to ensure continuity. ELIXIR offers one community model for identifying "Core Data Resources"*^24^, proposed by the ELIXIR Nodes and endorsed by their community.

*22
https://oecd.org/sti/sci-tech/2105199.pdf

*23
https://ga4gh.org/howwework/strategic-roadmap.html

*24
https://elixir-europe.org/platforms/data/core-data-resources

*25
https://apache.org/index.html#projects-list

*26
https://apache.org/foundation/policies/conduct.html

*27
https://incubator.apache.org/policy/process.html

*28
https://www.ga4gh.org/howwework/driver_project_applications.html

*29
https://www.idigbio.org/content/nsf-adbc-program-information

*30
https://dissco.eu/
