# Supplementary material for: Connecting data and expertise: a new alliance for biodiversity knowledge
Source: Biodivers Data J. 2019 Mar 8;7:e33679. doi: 10.3897/BDJ.7.e33679 (PMC6420472; doi:10.3897/BDJ.7.e33679)
Supplement: Supplementary material 1 — Annex A - Outputs from GBIC2 Working Groups [file bdj-07-e33679-s001.pdf]

Annex A - Outputs from GBIC2 Working Groups

Attendees at GBIC2 participated in parallel working groups to explore the major sociological and technical issues limiting progress in four component areas identified in the *Global Biodiversity Informatics Outlook (GBIO).*

This annex summarizes the issues identified for each component, as well as strategies and solutions each group identified for addressing the issues. Nearly all of these strategies and solutions would benefit from increased international coordination. In many cases, solving the issues identified may prove impossible without the widespread adoption of a common approach or service.

Biodiversity Knowledge Network

The Biodiversity Knowledge Network component (GBIO Culture layer) relates to the development of models, workflows and social networks to ensure community engagement. These include the recognition systems that a coordination mechanism could support to empower and remunerate those who contribute, curate or improve digital biodiversity information on biodiversity.

The sociological aspects of integration, change and engagement are key factors that the coordination mechanism will need to address from the start to ensure successful collaboration on a global scale. The stakeholder community—from data providers and data curators to data users—will need to gather its full range of skills to explore and seek solutions to the current obstacles in this landscape. Stakeholder engagement will be essential from the very beginning of the development of a future biodiversity knowledge network, and remain so at all stages. The provision of concrete, mutually agreed processes for data contribution, linking data and verifying assertions, as well as for professional recognition, are practical possibilities. But to facilitate community engagement, the network must develop trust in the system and remain steadily assured of the quality of the data.

Current impediments to delivery

The GBIC2 Biodiversity Knowledge Network working sessions identified the following as the major impediments that affect engagement or limit mobilization and verification of data on biodiversity, with the lack of coordination and prioritization of efforts seen as an overarching weakness in today's landscape.

1. **Practical and technical obstacles** exist in relation to data curation and linkage, the need to guarantee the accuracy and quality of data and to data traceability. Feedback loops must be established to ensure bidirectional flow of data to support trusted clearing house mechanisms for data. Access to technology and digital expertise varies between different countries and communities, and this creates practical and technical barriers to data capture and mobilization. In the future, machine learning and more automated data capture need to be accommodated, as the time needed to gather and standardize data into machine-ready formats (for sharing and connecting via unique identifiers) is currently a limiting factor.
2. **Behavioural and cultural hurdles** relate to the need to inspire 'trust in the system', so that an environment of 'data sharing confidence' is built upon a global scale. Closely linked to

this, the data provided to users must be both accurate and up-to-date. Community confidence is limited at present, as accreditation or acknowledgement for contributions, such as data provision, data annotation, data verification, and expert contributions, is neither assured nor consistently tracked. Many individuals who make contributions of different kinds (whether online or not) are not reliably recognized. As a result, the scope of tangible benefits available to individuals, collaborative teams or organizations receive for their efforts is limited. The lack of personal traceability also links directly to concerns over the accuracy and quality of the available data. Differences in data-sharing cultures, whether across borders or even in different communities, need to be understood and addressed.

3. **Scientific and taxonomic impediments** limit the state of our knowledge of species on Earth and fall short of fulfilling the fundamental need for support and enhancement of ongoing efforts to document, describe and name species. The lack of a dynamic operational system for integrating up-to-date knowledge and presenting a consensus classification across all organisms hinders taxonomic research; it also compromises the organization of biodiversity data into a recognizable format in ways that permit data linking, either to records of the same entity or to different classes of data (for example, DNA, trait, phenological or ecological data) for the same organism. It is difficult for today's end users to evaluate whether different data sources refer to the same taxonomic entity or not. Gaps in current knowledge have a negative impact on trust in the data as a whole, which further discourages taxonomic experts or taxonomic and nomenclatural organizations from committing more fully to managing the data.

Potential strategies and investments to address impediments

The working group identified the following as significant potential efforts that could significantly address the impediments to delivery.

1. **Address user problems and user needs** through a thorough baseline assessment to understand the actual difficulties encountered by the different stakeholder communities, particularly current data providers and data users. The assessment should identify areas for which solutions already exist and those where new ones are needed. It would also be beneficial to solicit opinions from current and potential data user and data provider communities about what they want or need from an interconnected knowledge system for global biodiversity. Bringing their problems and needs to the forefront will permit them to be used in prioritizing efforts at a number of different levels and support a culture of market-driven development.
2. **Universal software workbench and data management system** – Data providers need a set of consistent web services to facilitate data ingestion, validation and curation, quality assessment, assignment of identifiers, and data management functions. Tools built on these services would assist in community engagement and provide substantial economic value for stakeholders themselves. Integrated into institutional, regional or national systems, such services could add value and quality to the data and form the informatics backbone of a global biodiversity information platform. The services could also facilitate the standardization, handling and cross-linking of data, as the provision of unique identifiers allows data users to traverse bidirectional links between data objects, while speeding up data mobilization and increasing data quality in the process. The provision of rich data analysis options (geospatial, semantic, machine learning, etc.), supported by supercomputing clusters, would allow end users to work with the data as part of the mobilization process. The creation of a universal

software workbench and data management system would equip the stakeholder community for complementary benefits around all stages in data capture, data ingestion and data analysis. The provision of web-based services would go some way toward addressing the problem of differential access to technical capabilities (IT resources, developers) experienced by the providers and the users of data.

3. **Authentication and citation system for taxonomic knowledge and biodiversity data** – Two main elements are included here: first, the creation of a fair, reliable and equitable citation and recognition system for contributors of all types (institutions, individuals, associations, etc.,), and second, targeted lobbying and advocacy efforts to ensure that researchers receive recognition for citations linked to digital data contributions or annotations as well paper citations, and that these are considered during the process of assessing researcher impact. Recognition given to biodiversity scientists or other experts, as well as to the contributors of specimen, observation or other types of data, is an important form of support for individual scientists, their institutions, and science. It also delivers incentives to drive further contributions and to curate and improve the quality of the existing data. A trusted citation and data authentication system is key to both contributors and end users.
4. **Consensus Tree of Life and nomenclatural backbone** – The creation of a consensus phylogenetic classification and nomenclatural framework within a global biodiversity information network will facilitate scientific advancement as well as biodiversity data mobilization, as a commonly recognized and collaboratively agreed tool allows data to be structured and more accurately linked. A consensus nomenclatural backbone is core to all other categories of data, as names provide us with a consistent means of communicating about the data available for each entity. A clearing house mechanism for names and taxonomy would ensure the ability to link different kinds of information to species names and provide a platform for raising and highlighting issues related to names so that expert communities can contribute to resolving them and funders can see what issues remain within our understanding of the species on Earth.
5. **Engagement with stakeholder communities and expert groups** – A broad baseline study of the stakeholder community on data sharing activities, data sharing benefits and data gap analysis would establish the foundations from which to build workflows and best practices. The tracking of data publication and the subsequent electronic availability of data, data citation, and data use and reuse provides metrics for providers, as well as feeding into the coordination mechanism. The establishment of standards, best practices and operating norms will go a long way toward ensuring the delivery of quality data in forms that can be used immediately by the wider community. Promoting the single input of quality data and subsequently promoting universal data availability and connectivity saves both time and money, but this also facilitates the exploitation of the data by reducing duplication of effort and increasing data viability. The documentation of benefits secured through ongoing data sharing would contribute to the overall coordination and prioritization mechanism. Reducing gaps in data (taxonomic, geographic, territorial, cultural) increases the predictive value of the data itself. Huge specialist expertise can potentially be mobilized via engagement with professional and amateur societies and other expert groups. Coordinated ‘gap-filling’ activities could also stimulate community input into the shared resource: the biodiversity information network.
6. **Culture value through capacity building, leadership and services** – A culture of sharing needs to be built and supported, with the inherent need to establish trust built into both the

contribution model and the governance model. In order to foster a spirit of cooperation, the empowerment and training of stakeholders, the provision of services, solving of problems and the establishment of skill-building pathways are all important. In this context all data sources – large and small – should be embraced, with the knowledge that for local data contributors and users the data must be locally relevant as well as being locally accessible. Supporting smaller communities to share and contribute data as well as larger ones will help ensure equitable contributions.

Published Materials

The Published Materials component (GBIO Data layer) relates to both sociological and technical challenges. Printed and semi-structured documents have long served as the primary means for disseminating biodiversity knowledge. How can we transform the knowledge held in this system into accessible digital formats while ensuring that relevant information can be merged with structured data from other sources?

The Published Materials group outlined a number of current issues hindering or preventing discovery, access, linkage, or otherwise full use of published materials and the data they contain. These issues range from direct access to a given publication to larger scale data-mining capabilities. Additionally, the group noted that the community might benefit from increased capacity for publishers to accommodate the range and volume of materials being produced for publication (including taxonomic treatments, research papers, and other multimedia formats) and for digital libraries and other organizations to digitize the legacy literature.

Current impediments to delivery

The GBIC2 Published Materials working sessions identified the following limiting issues.

1. **Access restrictions** – With the growing cultural shift towards open access to research, paywalls and copyright restrictions increasingly act as a major impediment to access and use of scientific data, information, and knowledge. The complexities of copyright law and inconsistencies across international boundaries can further complicate matters. In addition, rights claims can be and are being asserted on digital surrogates of out-of-copyright publications. Assigning DOIs to such content and then placing them behind a paywall further compounds the issue by adding a barrier to older content that is otherwise out of copyright.
2. **Unstructured Content** – As researchers increasingly rely on automated processes, there is also a need to improve the machine accessibility of the data within published materials. Whether materials have been created in native digital formats, represent recent print publications, or derive from legacy literature, full access to these materials—including those presented in open access forms—will depend on greater consistency in the structure of publications; the use of persistent and unique identifiers; and improvements to standard vocabularies, metadata schemas, and markup to facilitate text and data mining. The proliferation of relevant technology solutions for both consumptive and non-consumptive access (e.g., GeoDeepDive[1], HathiTrust Research Center[2]) could help facilitate both the use

[1] https://geodeepdive.org
[2] https://www.hathitrust.org/htrc

of these tools and their integration with data aggregators, thus enabling linking between taxonomic treatments and occurrence records and other significant data objects.

Potential strategies and investments to address impediments

The group identified the following initiatives as ways to address some of the issues above. The proposed investments vary widely in cost and effort but this variation does not necessarily reflect the impact. The sequence is arbitrary.

1. **Global system to support linked biodiversity data from published material** – Establish infrastructure and resources (people and hardware) to support identifiers, ontologies, standards, and governance. Good tools are required for data extraction. This would involve establishing and maintaining interlinked registries of identifiers (with associated resolution services) operating at global scale. A standards-based platform is required to enable authors and publishers to capture OCR representations and to maintain multilingual semantic metadata on content and on the full chain of attribution and provenance. Training sets are required to assist machine learning approaches to address the backlog.
2. **Connecting taxon names to the published literature** – Biodiversity aggregators should include links to the definitive versions of the literature online (via DOIs). If behind a paywall, they should also include link to a freely available copy.
3. **Managing datasets connected with published literature** – Clear protocols and enforcement mechanisms should be established to ensure proper citation of data sets within literature so that these can become more discoverable.
4. **Assigning DOIs to historical literature** – Ensure versions of historical literature are discoverable via DOIs and offered in accordance with FAIR principles.
5. **Organization/Institution IDs** – An official, sustainable registry for biodiversity repositories would facilitate linking publications to specimens and reinforce the value of collections and institutions.
6. **Open digital publishing tools** – Institutional publishers need functional solutions to produce structured, dynamic, semantically enhanced publications.
7. **Community agreement on how publications are produced** – It is important to address the current lack of standards, variation in solutions, and interoperability issues.
8. **Standards for persistent identifiers and metadata for all data classes** – Standards and software solutions are required to support reliable references to specimens, observations, traits, species interactions, and multimedia objects.
9. **Adding semantics to link information to legacy literature** – Mechanisms are needed to enhance literature with direct links to resources such as the Catalogue of Life via resolvable identifiers. This implies the need for robust global authorities to maintain catalogues and registries of such identifiers.
10. **Open licenses for media** – Promoting the use of open licenses for the media in publications will make these components available for reuse.

Integrated Occurrence Data

The Integrated Occurrence Data component (GBIO Evidence layer) focuses on organizing all evidence for the recorded occurrence of species in time and space. By bringing together data from all relevant sources, this effort aims to document the occurrence and abundance of all

species throughout the world, at the finest possible scales and for all time periods, while supporting evaluation of the coverage, completeness and quality of these resources.

At the beginning of the workshop, the shared understanding of the target area's goal was refined as: "Create a dynamic, unified infrastructure framework (collection of methods and mechanisms) through which any person can contribute/find/map/process/assemble existing digital or non-digital evidence of organisms existing or not existing at a certain time and place for any study, while maintaining access to the data in its original form, and the provenance of any altered form."

Current impediments to delivery

The GBIC2 Integrated Occurrence Data working sessions identified the following impediments and areas in which current solutions are insufficient.

1. **User needs** – It is important to understand target audiences and to deliver user friendly solutions for each. The broader community represented by the public needs information presented in different forms and using different language from expert communities. Understanding of community requirements can support and deliver demand-driven prioritization of tools, documentation and services that are functional and accessible to users without high levels of technical expertise. Solutions should flexibly accommodate the range of expectations, including rapid access to essential information and more exhaustive and potentially time-consuming processing for complex questions. The overall need is to focus on a culture of service.
2. **Data content** – Data content and services need to be robust to gain trust to serve key audiences, such as government agencies, but information may be valuable even when it is not considered "research grade". The focus is on good management of digital data. It is critical to store data in their original form, to allow future validation or mapping of data for new uses. In addition to presence-based occurrence data (evidence of an organism existing at a certain time and place), systems need to provide appropriate handling for evidence of absence (an organism not existing at a certain time and place). Support for data attribution is critical.
3. **Integration/interoperability** – It is not necessary to build a single, clean monolithic view of data, but the building blocks must exist that allow anyone to build a view to meet their needs. Collaborative development is important to reduce duplication of effort. Processes to integrate or aggregate data must aim to support lossless data exchange at all stages. Integration of occurrence data needs to include services that support the integration of these data with external data sources such as trait information, taxonomy, phylogeny and other geospatial datasets. All solutions will need to evolve to allow new knowledge to be added over time.

Potential strategies and investments to address impediments

The following were identified as significant potential investments to drive progress in this area.

1. **New occurrence data model** – Increase the richness and flexibility of the Darwin Core[3] standard to prepare data for integration. Darwin Core needs to evolve into a more expressive model to represent evidence of organisms, including the level of effort, recording method, time period and spatial extent for all sampling activities, and allowing representation of more

[3] https://dwc.tdwg.org/

relationships between concepts than the current ("star schema") model allows. This model should accommodate the full range of traditional occurrence, sample-event and checklist datasets and all related contextual elements such as people involved and associated measurements. The expected impact from addressing these issues would include both accommodating more data and communities and also improved linkages to external data sources (via careful data modelling or identifiers). A robust model for occurrence data also depends strongly on delivering a comprehensive taxonomic framework, managing stable identifiers and addressing a general lack in controlled vocabularies for properties that can be normalized. Backwards compatibility with Darwin Core and other standards is also important.

2. **Support for new data standards** – A wide range of ancillary data standards and catalogues could contribute to data quality and integration, including, for example, biographical details of botanical collectors. Information such as their names; their birth, death and activity dates; places they deposited their specimens; and collaborators, among other details, would aid research and validate data. The expected impact of such efforts would be to reduce duplication, to increase the possibility for data validation, and to support cross-collection integration. Other examples include: 1) adoption of ORCID identifiers for researchers; 2) adoption of standards to represent evidence of species interactions from different sources (allowing interdependencies to improve predictions of species distributions); and 3) standards to incorporate environmental DNA (eDNA) sources.
3. **Attribution in occurrence data** – Information should be organized on all forms of individual contribution to the development and improvement of any data. The expected impact would be to give credit for digital activity alongside other categories of research output (e.g. Altmetric[4]. Functional solutions will depend on stable identifier schemes, such as ORCID identifiers, and on addressing possible privacy concerns.
4. **Centralized occurrence ID (GUID) registration service** – An identifier scheme and associated minting services are required for associating GUIDs with all specimens and occurrence records. The expected impacts would be increased traceability of records and stable linkages between disparate records (tissues to vouchers, host to parasites, etc.). The challenges to overcome include guaranteeing adequate funding for long-term hosting and maintenance of all components, and the reality that different groups and collections vary in their technical capability to participate. The model for the International GeoSample Number scheme (IGSN[5]) includes features which may assist in these regards.
5. **Centralized registry of Institution and collection identifiers** – A comprehensive catalogue of institutions and collections is required, building on the examples including GRBio[6] and Index Herbariorum[7], supporting registration and curation of data objects widely referenced throughout biodiversity information resources. The expected impact would include unambiguous identification of specimen repositories and metadata to support planning and prioritization of digitization activity. The TDWG Natural Collections Descriptions standard (NCD)[8] is currently under revision and would provide a model for extensible

---

[4] https://www.altmetric.com/
[5] http://igsn.github.io/
[6] https://www.ncbi.nlm.nih.gov/pmc/articles/PMC5018115/
[7] http://sweetgum.nybg.org/science/ih
[8] https://www.tdwg.org/standards/ncd/

metadata management. GBIF is currently assuming responsibility for future hosting of GRBio and for developing its scope as a comprehensive catalogue of collections[9].

6. **Simple integration tools** – There is scope for significantly simplified versions of the tools offered to support reuse of integrated data. These may include spreadsheet templates, standard structures for databases and data files, documented application programming interfaces, etc. There should also be support for users who do not need to download data, but simply require pre-processed yet traceable data products (as in a legal context such as an environmental impact assessment).
7. **Capacity building to improve the data publishing and using for all data users** – Data publishers require support materials and guidance, including manuals, workshops (both face-to-face and virtual), forums and support communities. It is important to avoid duplication of efforts. Gathering feedback from users as they download and process data could help prioritize additional guidance materials. Guidance for users on best practice for data citation is particularly important.
8. **Training in occurrence data** – Institutional capability and overall data quality can benefit from training programmes that instruct data providers and curators in understanding key principles associated with occurrence data (such as stable identifiers, scale, resolution, best practices, how to use controlled vocabularies, linked data).
9. **Provenance and trusted source** – Standards and mechanisms should be developed and employed to allow data users to assess characteristics related to the provenance and trustworthiness of data, such as taxonomy reliability, suitability for policy and regulation uses, and data precision, among others.
10. **Tools (and programming libraries) for geographic data quality control** – Many initiatives and tools exist to assist with geospatial and geographic data quality, but there is no "one-stop shop" to access and evaluate available options.
11. **Unified aggregation layer** – A shared pipeline for initial harvesting and integration of data sources would enable collaboration around the range of existing aggregation services for occurrence data, while at the same time supporting the different, additional processing routines required by different geographic and thematic communities. This system would enable more efficient and collaborative use of both financial and human resources. A single data pipeline could standardize common functions and data manipulations and combine extended processing steps as part of a unified whole. The expected impact would include stable identifier management; improvements to traceability, annotation and attribution of data; reductions in effort for each aggregation service; and simplified end-user experiences. A unified approach could also establish a platform for innovative development of a new generation of tools for publishing, accessing and curating data.
12. **Data richness indicators** – Metadata and data products should offer easy access to additional information about the original source or to the original source itself, especially in cases where publicly available data do not represent the full richness of the original. Challenges include developing effective standards for communicating the richness of additional information available at source and handling of information that is dispersed across multiple storage locations and formats.
13. **Multilingual data conversion support** – Given the diverse international community contributing and using data, language differences are a significant limiting factor. Better

[9] https://www.gbif.org/document/36j6HhbR4kOMY6oqcamEMk/

standardization and interpretation of data could be facilitated via semi-automatic smart tools that map natural language to standard vocabularies, combined with curation by human beings to assure the best quality, probably via a crowdsourcing model. Such support should be integrated across the entire data chain.

14. **Remote sensing biodiversity data extraction suite** – Machine-learning approaches are increasingly capable of unlocking species-level information from remote-sensing data. With the potential for very large volumes of repeated data to become accessible from these sources, they should be integrated as part of the data pipeline for integrated occurrence data.

Trends and Predictions

The Trends and Predictions component (GBIO Understanding layer) component aims to integrate historical data and changes over time to create models that support decision-making, make biodiversity estimates and predict the potential impact of changing conditions anywhere on Earth.

The GBIC2 Trends and Predictions working sessions explored the broad systemic issues that affect efforts to build complex integrative models and analyses of biodiversity patterns, trends and future predictions, including the use of data from domains outside biodiversity research.

Current impediments to delivery

The sessions identified the following broad areas as major impediments to using biodiversity data to deliver complex modeled knowledge products that fulfil research and policy needs.

1. **New data** – To set effective priorities for data mobilization, we need a clear understanding of what data are needed and what data are already available. Establishing clear priorities for new data mobilization will help to alleviate the tension between data supply and demand and to address the growing complexity of data value chains as increasing volumes of data are integrated. Following such a strategy, it will be possible to scale up the mobilization of well-curated biodiversity data and target high-priority biodiversity data gaps, with a particular focus on standardized sampling-event data from underrepresented areas.
2. **Research and Development** – It is essential to deal with data quality and uncertainty associated with data. Data quality concerns limit the downstream usability in demand-driven policy outcomes. We need to implement governance and technical solutions that improve the tracking of uncertainty within and between datasets, including highlighting data gaps and improving metadata to support as wide a range of data sources as possible. Without this, progress will remain too slow to meet the demands for evidence-based biodiversity policy and management.
3. **Implementation** – True data integration and synthesis at the global scale is enormously challenging in the absence of an implementation plan. It is not clear whether it is better for the community to unite around centralized delivery mechanisms or, rather, to rely on shared standards, governance and accreditation. The former, while expensive to develop, could scale down costs over time, whereas the latter, though more cost-effective, has the potential downside of creating a suite of alternative, potentially conflicting and difficult-to-use end products. Regardless of approach, it is important to focus on developing trust between organizations across regions and countries, and ensuring the open sharing of tools, best practice, IT infrastructure and resources for capacity building.

4. **Communication** – Better mechanisms are needed to translate the evolving needs of multiple different knowledge user groups into high-quality indicators and visualization tools. This should include agreement on formal frameworks for global-scale reporting on international biodiversity policy to support national needs.
5. **Cross-cutting actions** – Strengthening of the perceived value of biodiversity data and its use is essential to improve its collection and analysis. Communication and promotional materials for this purpose could be tailored according to regional and thematic needs.

Potential strategies and investments to address impediments

The investment areas identified included

- technical aspects, like quality assurance, capture of sampling event data, model integration, standard protocols
- bridging gaps between the supply from data providers and scientists to the demand from governments and user groups
- strategic/smart communication/engagement with decision-makers and public stakeholders
- improving uptake through capacity building and training as well as national action plans

Perhaps the most significant need is to develop an understanding of which gaps in existing data are most significant; how these gaps compromise modelling of trends and predictions; and what mechanisms can best address these gaps.

The complexity and multidisciplinary nature of modelling complex systems using heterogeneous data means that all of the following aspects require large-scale coordination to maximize benefits and efficiency.

1. **Addressing data gaps** – Establish mechanisms for identifying the shortfall between available data and adequate estimation of patterns and trends across all regions and scales, and for stimulating mobilization and capture of new data, particularly sampling-event data, for underrepresented areas.
2. **Metadata and quality control** – Develop governance and technical solutions, including quality assurance mechanisms, so that the strengths and weaknesses of different datasets are well understood and documented with machine-readable metadata that includes information on provenance and transformations.
3. **Data curation** – Ensure that data sources—in particular on species occurrences, absences and abundance—are well-curated, authoritative, comprehensive and fit for use, with concrete data management policies for data derived from citizen science and other sources.
4. **Funding** – Define the scope of planned data modelling activities and the sources of sustainable funding to alleviate the tension that may exist between supply and demand as data volumes grow.
5. **Cross-discipline** – Coordinate formats, vocabularies, services and modelling systems with those in use by disciplines other than biodiversity, so that digitized information can be combined and interact more readily and effectively.
6. **Coordinated planning** – Develop a clear, forward-looking implementation plan to deal with the challenge of data integration at a global scale, recognizing the diverse stakeholders that contribute data, resources or expertise. This plan should include coordination of access to supercomputing facilities and capacity building. Formal frameworks for global-scale reporting

include CBD National Reports[10], UN Sustainable Development Goals (https://www.un.org/sustainabledevelopment/sustainable-development-goals/), and the Sendai Framework for Disaster Risk Reduction[11]; and global, regional, and national strategies and action plans for data mobilization of relevant data types.

7. **Reusable tools and standards** – Adopt open source and collaborative models to develop and document reusable standards, software modules, data management protocols and analytic workflows.
8. **Capacity enhancement** – Plan and resource capacity enhancement efforts across the full chain of data management from data capture to data analysis.
9. **Equitable access** – Focus on equitable access to biodiversity information by stakeholders in all regions and at all scales, particularly through high-quality indicators and visualization tools.
10. **Communication** – Establish communication with all stakeholders, including decision-makers and the public, to improve perceptions of the value of biodiversity data; to enhance understanding of data needs, data availability and knowledge products; and to secure broader engagement in all stages of data production, management and use.
11. **Community trust** – Build trust and cooperation between different organizations and sectors in the context of bilateral or multilateral international cooperation for coordinated infrastructure development.

---

[10] https://www.cbd.int/reports
[11] https://www.unisdr.org/we/coordinate/sendai-framework
