# Supplementary material for: Connecting data and expertise: a new alliance for biodiversity knowledge
Source: Biodivers Data J. 2019 Mar 8;7:e33679. doi: 10.3897/BDJ.7.e33679 (PMC6420472; doi:10.3897/BDJ.7.e33679)
Supplement: Supplementary material 2 — Annex B - List of GBIC2 Attendees [file bdj-07-e33679-s002.pdf]

Annex B - List of GBIC2 Attendees

Aaron Wilton, Landcare Research, New Zealand
Alex Asase, University of Ghana, Ghana
Alex Mitchell, European Bioinformatics Institute, United Kingdom
Ana Casino, Consortium of European Taxonomic Facilities, Belgium
Anabela Plos, Museo Argentino de Ciencias Naturales, Argentina
Anders Finstad, Norwegian University of Technology and Science, Norway
Anders Telenius, Naturhistoriska Riksmuseet, Sweden
Andrea Hahn, GBIF Secretariat, Denmark
Andrea Portela, Ministério da Ciência, Tecnologia, Inovações e Comunicações, Brazil
Andrew Rodrigues, GBIF Secretariat, Denmark
Andy Bentley, Kansas University Biodiversity Institute, United States
Anne-Sophie Archambeau, GBIF France, France
Bob Corrigan, Encyclopedia of Life, United States
Brigitte Baptiste, Instituto de Investigación de Recursos Biológicos Alexander von Humboldt, Colombia
Carolyn Sheffield, Smithsonian Institution, United States
Chih-Jen Ko, Academia Sinica, Taiwan
Chloe Chester, Museum National d'Histoire Naturelle, France
Chris Gentle, Western Australian Biodiversity Science Institute, Australia
Christian Elloran , Asean Biodiversity, Philippines
Christoph Haeuser, Museum für Naturkunde, Germany
Corinna Gries, University of Wisconsin, United States
Cristina Villaverde, GBIF Spain, Spain
Daniel Noesgaard, GBIF Secretariat, Denmark
Dave Martin, Atlas of Living Australia, United Kingdom
David Patterson, WWF UK, United Kingdom
David Thau, Google / WWF, United States
Dimitris Koureas, DiSSCo, Netherlands
Dirk Steinke, Centre for Biodiversity Genomics, Canada
Dmitry Schigel, GBIF Secretariat, Denmark
Don Doering, JRS Foundation, United States
Donald Hobern, GBIF Secretariat, Denmark
Edwin van Huis, Naturalis, Netherlands
Eun-Shik Kim, Kookmin University, Korea
Fatima Parker-Allie, South African National Biodiversity Institute, South Africa
Francois Hissel, Agence française pour la biodiversité, France
Gil Nelson, iDigBio, United States
Gregoire Dubois, Joint Research Centre, Italy
Hamish Holewa, Atlas of Living Australia, Australia
Ina Smith, Academy of Science of South Africa, South Africa
Isayvani Naicker, African Academy of Sciences, Kenya
Ismaël Mejía, Apache Software Foundation, France
James Hanken, Museum of Comparative Zoology, Harvard University, United States
James Macklin, Agriculture Canada, Canada
Jerry Lanfear, ELIXIR, United Kingdom
Jim Beach, Kansas University Biodiversity Institute, United States
John Wieczorek, Museum of Vertebrate Zoology, University of California Berkeley, Argentina
Jose Fortes, iDigBio, United States

Juan Miguel Gonzalez Aranda, Lifewatch EU, Spain
Junko Shimura, Convention on Biological Diversity, Canada
Keping Ma, Chinese Academy of Sciences, China
Kevin Thiele, Australian Academy of Science, Australia
Kyle Copas, GBIF Secretariat, Denmark
Laetitia Navarro, German Centre for Integrative Biodiversity Research, Germany
Larry Page, iDigBio, United States
Laura Russell, GBIF Secretariat, Denmark
Lawrence Monda, National Museums of Kenya, Kenya
Leonard Krishtalka, Kansas University Biodiversity Institute, United States
Liliana Ballesteros, Muséum national d'Histoire naturelle, France
Lyubo Penev, Pensoft, Bulgaria
Maria Uhle, Belmont Forum, United States
Marta Iturribarria, Gobierno Vasco, Spain
Mélianie Raymond, GBIF Secretariat, Denmark
Melodie McGeoch, Monash University, Australia
Michel Guiraud, Muséum national d'Histoire naturelle, France
Michelle Price, Consortium of European Taxonomic Facilities, Switzerland
Mikhail Kalyakin, Zoological Museum of Moscow University, Russia
Nicky Nicolson, Royal Botanic Gardens Kew, United Kingdom
Nicole Kearney, Museums Victoria, Australia
Olaf Banki, Catalogue of Life Plus project, Netherlands
Ole Seberg, Statens Naturhistoriske Museum, Denmark
Patricia Koleff, CONABIO, Mexico
Paula Zermoglio, TDWG, Argentina
Peter Desmet, Instituut Natuur- En Bosonderzoek, Belgium
Peter Schalk, Catalogue of Life, Netherlands
Philippe Grandcolas, Muséum national d'Histoire naturelle, France
Piotr Tykarski, University of Warsaw, Poland
Prabhakar Rajagopal, Strand Life Sciences, India
Quentin Groom, Botanic Garden Meise, Belgium
Ramona Walls, University of Arizona, United States
Riza Batista-Navarro, University of Manchester, United Kingdom
Rob Guralnick, Florida Museum of Natural History, United States
Rob Zschernitz, The Field Museum, United States
Robert Hanisch, National Institute of Standards and Technology, United States
Robert Montoya, Indiana University, United States
Roland Roberts, National Science Foundation, United States
Roseli Pellens, Muséum national d'Histoire naturelle, France
Scott Loarie, iNaturalist, United States
Serge Belongie, Cornell University, United States
Sharon Grant, The Field Museum, United States
Shyama Pagad, IUCN SSG Invasive Species Specialist Group, New Zealand
Sky Bristol, Ocean Biogeographic Information System, United States
Takeshi Osawa, Tokyo Metropolitan University, Japan
Talia Karim, University of Colorado, United States
Tanya Abrahamse, GBIF Governing Board, South Africa
Thomas Hickler, Senckenberg Biodiversity and Climate Research Centre,
Germany Thomas Pape, International Commission on Zoological Nomenclature,
Denmark Tim Hirsch, GBIF Secretariat, Denmark
Tim Robertson, GBIF Secretariat, Denmark

Tsuyoshi Hosoya, National Museum of Nature and Science, Japan
Walter Berendsohn, Botanischer Garten und Botanisches Museum Berlin, Germany
Wim Hugo, ICSU World Data System, South Africa
Wouter Addink, Naturalis, Netherlands
Yichuan Shi, UNEP World Conservation Monitoring Centre, United Kingdom
