# Supplementary material for: Connecting data and expertise: a new alliance for biodiversity knowledge
Source: Biodivers Data J. 2019 Mar 8;7:e33679. doi: 10.3897/BDJ.7.e33679 (PMC6420472; doi:10.3897/BDJ.7.e33679)
Supplement: Supplementary material 3 — Call for an alliance for biodiversity knowledge [file bdj-07-e33679-s003.pdf]

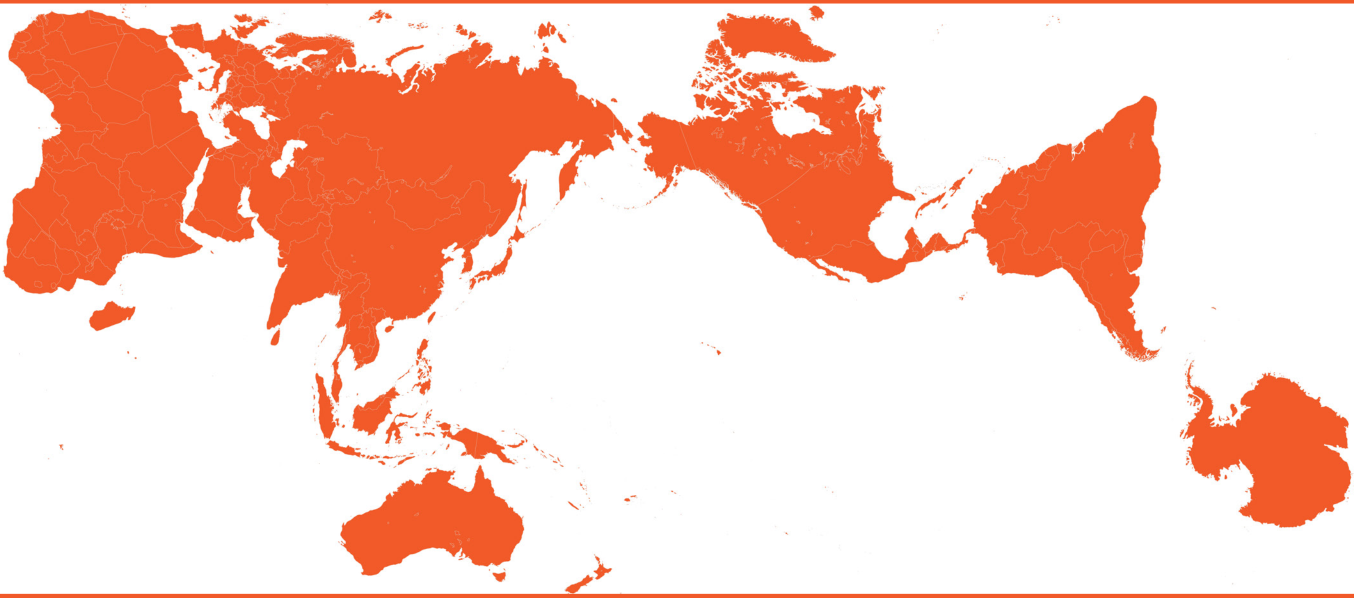

Call for
an alliance for biodiversity knowledge

VISION

There has been major progress over the last two decades in digitizing historical knowledge of biodiversity and in making biodiversity data freely and openly accessible. Interlocking efforts bring together international partnerships and networks, national, regional and institutional projects and investments, and countless individual contributors. These collaborations span across diverse biological and environmental research domains, government agencies and non-governmental organizations, citizen science and commercial enterprise.

However, current efforts remain inefficient and inadequate to address the global need for accurate data on the world's species and on changing patterns and trends in biodiversity. Significant challenges include:

- imbalances in regional engagement in biodiversity informatics activity
- uneven progress in data mobilization and sharing
- the lack of stable persistent identifiers for data records
- redundant and incompatible processes for cleaning and interpreting data
- the absence of functional mechanisms for knowledgeable experts to curate and improve data

Recognizing the need for greater alignment between efforts at all scales, the Global Biodiversity Information Facility (GBIF) convened the second Global Biodiversity Informatics Conference (GBIC2) in July 2018 to propose a coordination mechanism for developing shared roadmaps for biodiversity informatics. GBIC2 attendees reached consensus on the need for a global alliance for biodiversity knowledge, learning from examples such as the **Global Alliance for Genomics and Health** (GA4GH) and the open software communities under the **Apache Software Foundation**. These initiatives provide models for multiple stakeholders with decentralized funding and independent governance to combine resources and develop sustainable solutions that address common needs.

Increased cooperation, improved management of existing data and coordinated development of novel sources of data could deliver an integrated, interlinked knowledge base for all aspects of biodiversity—one freely and openly disseminated to everyone who has need or interest in using it. Achieving such a system would enable us to embed current scientific understanding of biodiversity within rational and timely decision-making processes that support a sustainable future.

Supplementary Material 3: Call for an alliance on biodiversity knowledge
doi:10.3897/BDJ.7.e33679.suppl3

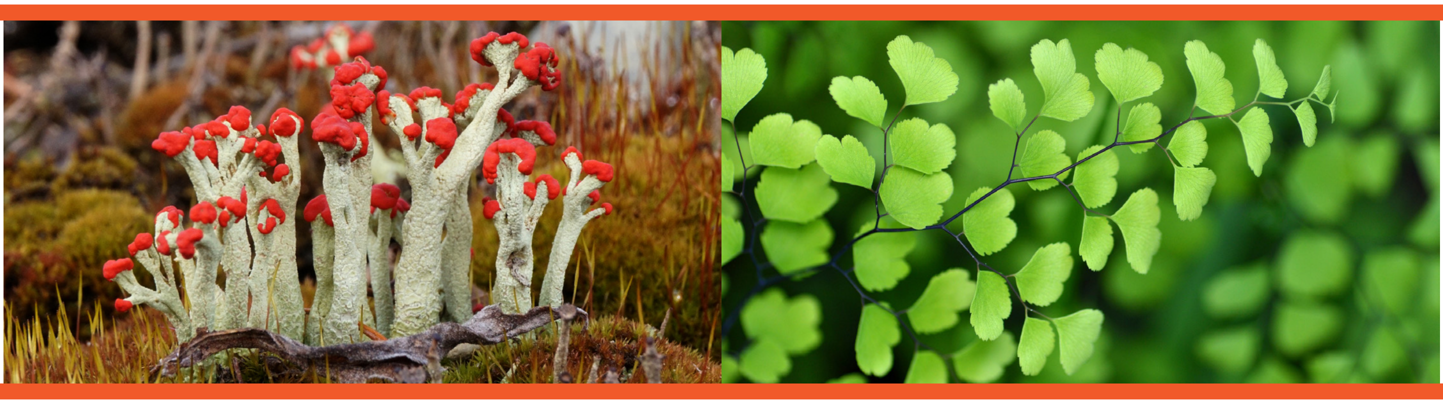

AMBITIONS

The attendees at GBIC2 proposed the following multifaceted vision to define the ambitions for an alliance for biodiversity knowledge:

SUPPORT FOR SCIENCE AND EVIDENCE-BASED PLANNING

1. To deliver biodiversity knowledge and understanding in forms which support critical research requirements and enable biodiversity to be correctly measured and assessed for societal goals
2. To serve as the foundation for basic research in biodiversity and information science that serves human understanding of the functioning and state of natural systems
3. To provide a platform for continuous growth in understanding of biodiversity by preserving, building on and improving existing knowledge

SUPPORT FOR OPEN DATA AND OPEN SCIENCE

4. To remove barriers to free and open sharing of data and to the adoption of FAIR data principles (**Wilkinson et al. 2016**) for biodiversity data
5. To describe all data resources with rich metadata that supports present and future reuse
6. To ensure all data resources are preserved in stable and persistent trusted repositories
7. To enable collaborative curation, annotation and improvement of all data by any relevant experts and expert communities
8. To enable all contributors of knowledge or expertise to have their contributions fully recorded, acknowledged and credited
9. To track the provenance and attribution of all sources of information

SUPPORT FOR HIGHLY-CONNECTED BIODIVERSITY DATA

10. To mobilize structured digital representations of historical data sources, including museum collections and literature
11. To ensure that all new observations and measurements are accessible in structured digital representations as rapidly as possible after capture
12. To enable the combination, querying and analysis of different classes of biodiversity information (distribution, traits, genes, etc.) as an interconnected whole
13. To work with other research communities and infrastructures to achieve interoperability with earth observations, social science data and other resources

Image credits. p.1: AuthaGraph map of the world with political borders adapted from **Justin Kunimune**, licensed under CC BY-SA 4.0. ***p.2:*** *(left)* British soldier lichen (*Cladonia cristatella*), by Mike Lewinski via **iNaturalist**, licensed under CC BY 2.0; (*right*) Black maidenhair fern (*Adiantum capillus-veneris*) by Robby Deans via **iNaturalist**, licensed under CC BY-NC 4.0.

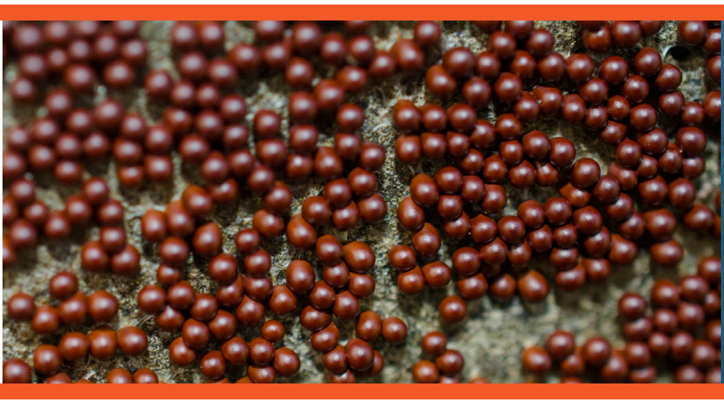

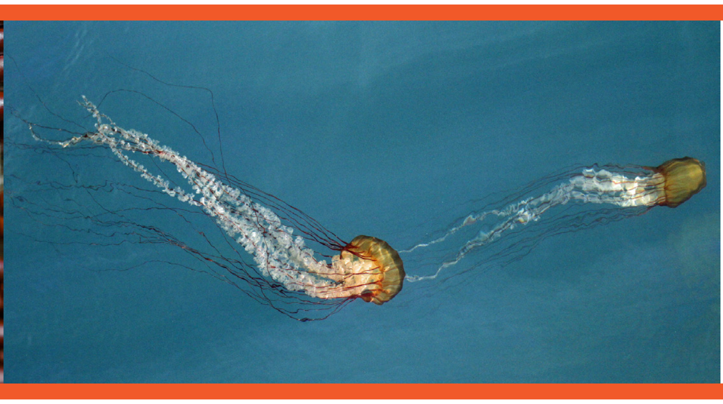

SUPPORT FOR INTERNATIONAL COLLABORATION

14. To address capacity needs around biodiversity informatics in all regions and in all sectors
15. To secure funding to maintain services and component that the community recognizes as critical elements within a distributed knowledge infrastructure
16. To develop flexible, collaborative approaches to designing, building and sustaining all components of this distributed knowledge infrastructure
17. To enable stakeholders in every country and region to adopt and benefit from advances in infrastructure, tools, services, practices and capacity
18. To enable full participation of and collaboration with all stakeholder groups in all regions at all stages, from data generation to analysis and application
19. To enable data repatriation that supports science and policy-making in all countries and regions
20. To ensure effective access to, and use of, data at every scale—global, regional, national and local
21. To acknowledge and support the role of regional, national and local investments as critical and effective components within a global solution
22. To overcome barriers to data sharing or use arising from language or culture
23. To support the practical implementation of international agreements with reference to access and benefit sharing

NEXT STEPS

All stakeholders with an interest in the production, management, use and integration of data on the world's biodiversity are urged to contribute to establishing this proposed alliance for biodiversity knowledge by providing input to the following initial processes.

Further information, and opportunities to contribute to associated discussions, is maintained on the *alliance* website, biodiversityinformatics.org. Visit the *Discussions* tab on the website to contribute to discussions on each of these five areas. **Contributions in languages other than English are welcomed**.

EXPAND ENGAGEMENT

The workshop, its report, and this call to action have been prepared for the global community. Individuals and institutions with an interest in mobilizing, improving, integrating or using biodiversity information are encouraged to indicate their support by signing on in support and/or subscribing for further updates on the *alliance* website, biodiversityinformatics.org.

p.3: *(left)* Insect-egg mold (*Leocarpus fragilis*) by Tim Sage via **iNaturalist**, licensed under CC BY-NC 4.0; (*right*) Pacific sea nettle (*Chrysaora fuscescens*) by Robin Agarwal via **iNaturalist**, licensed under CC BY-NC 4.0.

EVALUATE MODELS

More work is required to address the needs of this complex and diverse stakeholder community, but models from other similar alliances, coalitions and consortia (such as the merit-based 'Apache Way' in use by open source software projects), can guide and inform longer term approaches. Important questions also remain around the basis of membership (individuals, institutional or both).

CLARIFY SCOPE AND TARGET OUTCOMES

Greater cooperation will undoubtedly bring significant benefits and efficiencies within the field of biodiversity informatics, but the ultimate goal is to deliver impact for science, policy and society. GBIC2 attendees proposed to engage diverse stakeholders—including research groups, taxonomic facilities, the CBD, IPBES, FAO, conservation bodies, and other user communities—to develop a set of defining questions and achievable use cases against which to measure progress. These should be sufficiently precise and detailed to guide priorities for collaborative planning, development and implementation.

MAP STAKEHOLDERS

Understanding the landscape of stakeholders for the work of the alliance is difficult, owing to the number of activities, their often overlapping missions, and their implementation via work programmes on different timelines and at different scales of responsibility. Unless this complexity is understood, there is great risk of inadvertent conflict or duplication of effort. GBIF will coordinate an initial network analysis—one with tightly and clearly scoped limits—seeking to outline the roles, responsibilities and relationships of major organizations, particularly at global, regional and national scales. This effort will help the alliance identify critical services that need to be created or sustained, and indicate opportunities for better alignment or unification.

ADOPT PROOF-OF-CONCEPT PROJECTS

A key goal for the alliance is to enable stakeholders to converge around shared needs and to incubate sustainable projects to deliver tools, services, models and resources that contribute to delivering an interconnected digital knowledge system. Formal processes will be required to prioritize, incubate, deliver and sustain such projects. In the short term, there is value in identifying current activities which support the vision for an open alliance and that can be adopted as proof-of-concept projects. Such projects can offer lessons that can be incorporated into future governance models, while at the same time giving early examples of alliance-based cooperation. We seek suggestions for suitable existing activities as candidates for early proof-of-concept projects, not just in software development or data management, but also in other areas like capacity enhancement and sustainability planning.

Hobern D, Baptiste B, Copas K, Guralnick R, Hahn A, van Huis E, Kim E-S, McGeoch M, Naicker I, Navarro L, Noesgaard D, Price M, Rodrigues A, Schigel D, Sheffield CA & Wieczorek J (2019) Connecting data and expertise: a new alliance for biodiversity knowledge. Biodiversity Data Journal. **doi:10.3897/BDJ.7.e33679**
