# Supplementary material for: Connecting data and expertise: a new alliance for biodiversity knowledge
Source: Biodivers Data J. 2019 Mar 8;7:e33679. doi: 10.3897/BDJ.7.e33679 (PMC6420472; doi:10.3897/BDJ.7.e33679)
Supplement: Supplementary material 4 — دعوة لتكوين تحالف لمعرفة التنوع البيولوجي [file bdj-07-e33679-s004.pdf]

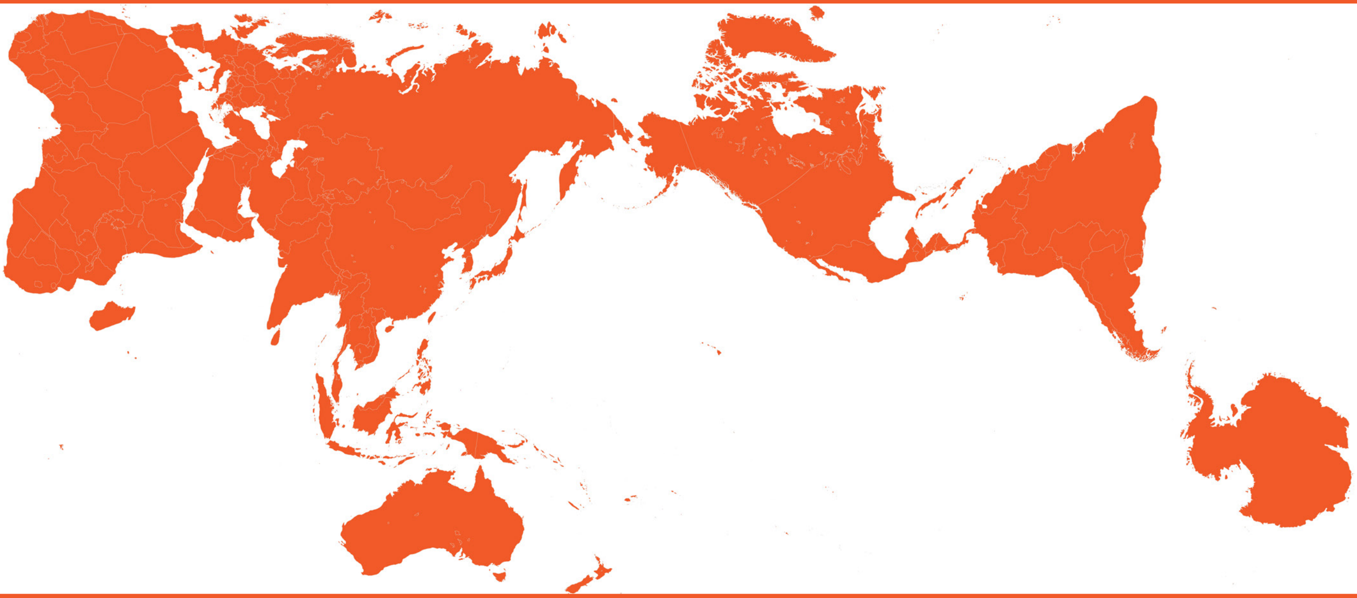

دعوة لتكوين تحالف لمعرفة التنوع البيولوجي

الرؤيه

حدث تقدم كبير خلال العقدين الأخيرين في التحويل الرقمي للمعرفة التاريخية للتنوع البيولوجي وفي الحصول على بيانات التنوع البيولوجي بحرية وعلانية. وتجمع الجهود المتشابكة بين الشراكات والشبكات الدولية والمشروعات والاستثمارات الوطنية والإقليمية والمؤسسية وعدد لا يحصى من المساهمين الأفراد. وتمتد هذه التعاونات عبر مجالات بحثية بيولوجية وبيئية متنوعة ووكالات حكومية ومنظمات غير حكومية وعلم المواطن ومؤسسات تجارية.

ومع ذلك، مازالت الجهود الحالية غير فعالة وغير كافية لتلبية الحاجة العالمية إلى بيانات دقيقة عن الأنواع في العالم وعن الأنماط والاتجاهات المتغيرة في التنوع البيولوجي. وتشمل التحديات الهامة ما يلي:

- اختلال توازن المشاركة الإقليمية في نشاط معلوماتية التنوع البيولوجي
- عدم انتظام التقدم في جمع البيانات وتقاسمها
- عدم وجود محددات ثابتة ومستقرة لسجلات البيانات
- زيادة وتضارب عمليات تنقية وتفسير البيانات
- عدم وجود آليات وظيفية للخبراء المطلعين لتنظيم وتحسين البيانات

وإدراكا للحاجة إلى مزيد من المواءمة بين الجهود على جميع الصعد، عقد المرفق العالمي لمعلومات التنوع البيولوجي (GBIF) في يوليه/ تموز 2018 من أجل اقتراح آلية تنسيق لتطوير خرائط (GBIC2) المؤتمر العالمي الثاني لمعلوماتية التنوع البيولوجي الطرق المشتركة لمعلوماتية التنوع البيولوجي. وتوصل الحضور في هذا المؤتمر إلى توافق في الآراء بشأن ضرورة وجود تحالف (GA4GH) عالمي لمعرفة التنوع البيولوجي، والتعلم من أمثلة مثل التحالف العالمي للمعلوماتية الجينومية والمتعلقة بالصحة ومجتمعات البرمجيات المفتوحة في إطار مؤسسة أباتشي للبرمجيات. وتقدم هذه المبادرات نماذج لجهات معنية متعددة بتمويل لامركزي وحوكمة مستقلة لجمع الموارد ووضع حلول مستدامة تلبي الاحتياجات المشتركة.

يمكن أن توفر زيادة التعاون وتحسين إدارة البيانات الحالية وتنسيق تطوير مصادر جديدة للبيانات قاعدة معارف متكاملة ومترابطة لجميع أوجه التنوع البيولوجي – قاعدة بيانات يتم نشرها بحرية وعلانية لكل من يحتاج إليها أو يهتم باستخدامها. سيمكننا تحقيق مثل هذا النظام من تضمين الفهم العلمي الحالي للتنوع البيولوجي في عمليات صنع القرار المنطقية وفي الوقت المناسب التي تدعم مستقبلًا مستدامًا.

Supplementary Material 4: دعوة لتكوين تحالف لمعرفة التنوع البيولوجي
doi:10.3897/BDJ.7.e33679.suppl4

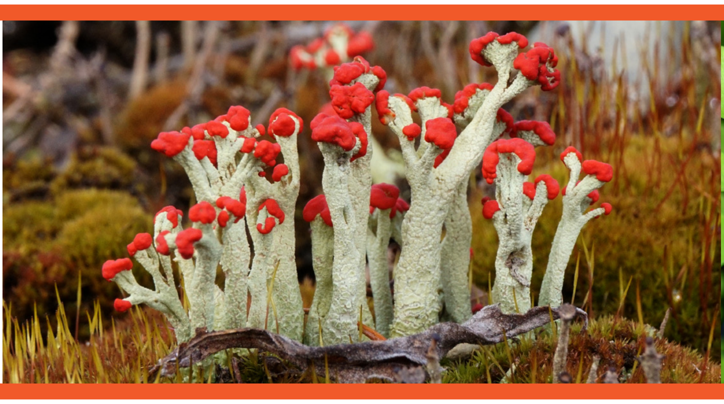 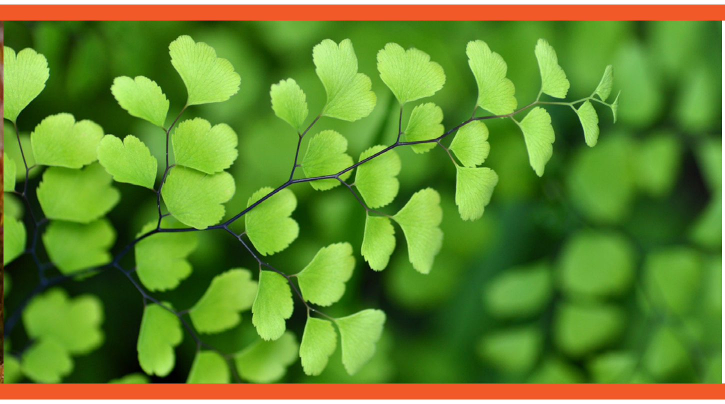

طموحات

:واقترح حضور هذا المؤتمر الرؤية متعددة الأوجه التالية لتحديد الطموحات لإنشاء تحالف لمعرفة التنوع البيولوجي

دعم العلم والتخطيط القائم على الأدلة

1. لكي يقدم معرفة وفهم التنوع البيولوجي في النماذج التي تدعم متطلبات البحوث الحرجة وتمكن من قياس وتقييم التنوع البيولوجي بشكل صحيح من أجل الأهداف المجتمعية
2. لكي يعمل بمثابة الأساس للبحوث الأساسية في مجال التنوع البيولوجي وعلم المعلومات التي تخدم فهم الإنسان لأداء النظم الطبيعية وحالتها
3. لكي يوفر محفلا للنمو المستمر لفهم التنوع البيولوجي عن طريق الحفاظ على المعرفة الحالية والبناء عليها وتحسينها

دعم البيانات المفتوحة والعلم المفتوح

4. لكي يزيل العوائق التي تحول دون تقاسم البيانات بحرية وعلانية واعتماد مبادئ البيانات التي يمكن العثور عليها والوصول إليها والقابلة للتبادل والتكامل والقابلة لإعادة استخدامها (FAIR data) لبيانات التنوع البيولوجي (ويلكنسون وآخرون 2016)
5. لكي يصف جميع موارد البيانات ببيانات تعريف البيانات الغنية التي تدعم إعادة الاستخدام حاليا وفي المستقبل
6. لكي يضمن الحفاظ على جميع موارد البيانات في مستودعات موثوقة ومستمرة ومستقرة
7. لكي يمكن أي خبير معني أو مجتمع من الخبراء من العناية بجميع البيانات وتفسيرها وتحسينها بشكل تعاوني
8. لكي يمكن جميع المساهمين بالمعرفة أو الخبرة من تسجيل مساهماتهم بالكامل والاعتراف بها والاعتماد عليها
9. لكي يتتبع مصدر وإسناد جميع مصادر المعلومات

دعم بيانات التنوع البيولوجي المتصلة ببعضها

10. لكي يحشد تمثيلات رقمية منظمة لمصادر البيانات التاريخية، بما في ذلك مجموعات المتاحف والأدب
11. لكي يضمن إمكانية الوصول إلى جميع الملاحظات والقياسات الجديدة في شكل تمثيلات رقمية منظمة بأسرع ما يمكن بعد جمعها
12. لكي يمكن جمع وتحليل والاستفسار عن مختلف فئات معلومات التنوع البيولوجي (التوزيع والصفات والجينات وغير ذلك) باعتبارها وحدة كاملة مترابطة
13. لكي يعمل مع مجتمعات البحوث والبنى التحتية الأخرى لتحقيق قابلية التبادل مع ملاحظات الأرض وبيانات العلوم الاجتماعية والموارد الأخرى

Image credits. p.1: AuthaGraph map of the world with political borders adapted from **Justin Kunimune**, licensed under CC BY-SA 4.0. ***p.2:*** *(left)* British soldier lichen (*Cladonia cristatella*), by Mike Lewinski via **iNaturalist**, licensed under CC BY 2.0; (*right*) Black maidenhair fern (*Adiantum capillus-veneris*) by Robby Deans via **iNaturalist**, licensed under CC BY-NC 4.0.

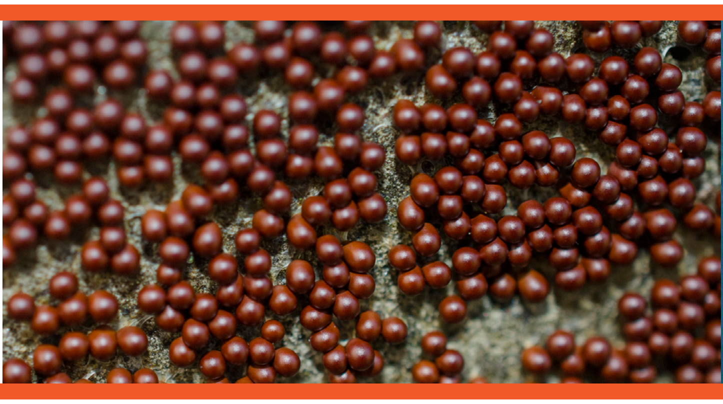

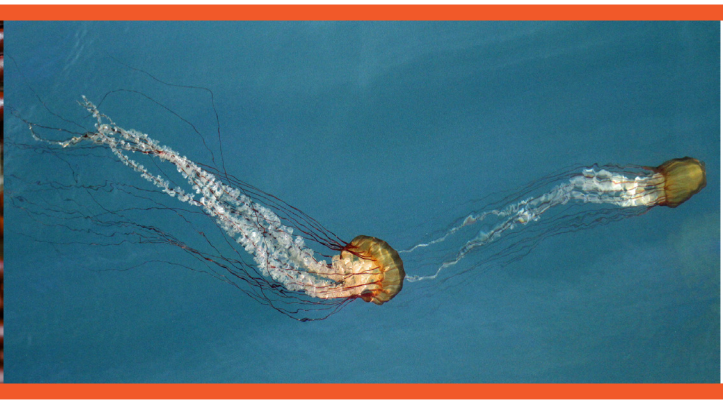

دعم التعاون الدولي

14. لكي يعالج الاحتياجات من القدرات حول معلوماتية التنوع البيولوجي في جميع المناطق والقطاعات
15. لكي يؤمن تمويلا للحفاظ على الخدمات والعناصر التي يعترف بها المجتمع كعناصر حاسمة داخل بنية أساسية معرفية موزعة
16. لكي يضع نُهج مرنة وتعاونية من أجل تصميم وبناء واستدامة جميع مكونات البنية التحتية المعرفية الموزعة
17. لكي يمكن الجهات المعنية في كل بلد وكل منطقة من تبني واستغلال أوجه التقدم في البنية التحتية والأدوات والخدمات والممارسات والقدرات
18. لكي يمكن المشاركة الكاملة والتعاون مع جميع فئات الجهات المعنية في جميع المناطق وجميع المراحل من توليد البيانات إلى التحليل والتطبيق
19. لكي يمكن إعادة البيانات التي تدعم العلوم وصنع السياسات في جميع البلدان والمناطق
20. لكي يضمن الوصول الفعال إلى البيانات واستخدامها على كل الصعد - العالمية والإقليمية والوطنية والمحلية
21. لكي يقر ويدعم دور الاستثمارات الإقليمية والوطنية والمحلية بصفتها عناصر أساسية وفعالة في إطار حل عالمي
22. لكي يتغلب على العوائق الناشئة عن اللغة أو الثقافة التي تحول دون تقاسم البيانات أو استخدامها
23. لكي يدعم التنفيذ العملي للاتفاقات الدولية فيما يتعلق بالحصول عليها وتقاسم المنافع

الخطوات التالية

ينبغي حث جميع الجهات المعنية المهتمة بإنتاج وإدارة واستخدام وتكامل البيانات المتعلقة بالتنوع البيولوجي في العالم على المساهمة في إنشاء هذا التحالف المقترح لمعرفة التنوع البيولوجي من خلال توفير مدخلات للعمليات الأولية التالية.

سيتم الاحتفاظ بمزيد من المعلومات وفرص المساهمة في المناقشات ذات الصلة على الموقع الإلكتروني للتحالف. يرجى زيارة علامة تبويب المناقشات على الموقع الإلكتروني للمساهمة في المناقشات حول كل من هذه المجالات الخم. والمساهمات بلغات غير الإنجليزية مرحب بها.

توسيع نطاق المشاركة

أعدت حلقة العمل وتقريرها وهذه الدعوة إلى العمل من أجل المجتمع العالمي. ويتم حث الأفراد والمؤسسات المهتمين بجمع أو تحسين أو تكامل أو استخدام معلومات التنوع البيولوجي على التدليل على دعمهم من خلال تسجيل الدخول بالدعم و / أو الاشتراك للحصول على مزيد من التحديثات على الموقع الإلكتروني للتحالف.

p.3: *(left)* Insect-egg mold (*Leocarpus fragilis*) by Tim Sage via **iNaturalist**, licensed under CC BY-NC 4.0; *(right)* Pacific sea nettle (*Chrysaora fuscescens*) by Robin Agarwal via **iNaturalist**, licensed under CC BY-NC 4.0.

تقييم النماذج

هناك حاجة إلى مزيد من العمل لتلبية احتياجات مجتمع الجهات المعنية المعقد والمتنوع هذا، ولكن تستطيع النماذج من التحالفات والائتلافات والاتحادات المماثلة الأخرى (مثل "طريقة أباتشي" المستندة إلى الجدارة والمستخدمة في مشروعات البرامج مفتوحة المصدر) أن ترشد وتنير النهج طويلة الأجل. وكذلك تظل الأسئلة الهامة قائمة على أساس العضوية (أفراد أو مؤسسات أو كليهما).

توضيح النطاق والنتائج المستهدفة

سيحقق المزيد من التعاون بلا شك منافع وفعاليات هامة في مجال معلوماتية التنوع البيولوجي، ولكن الهدف النهائي هو إشراك مختلف الجهات المعنية بالتنوع - بما في ذلك GBIC2 التأثير على العلم والسياسة والمجتمع. واقترح الحضور في المؤتمر المجموعات البحثية والمرافق التصنيفية واتفاقية التنوع البيولوجي، والمنبر الحكومي الدولي للعلوم والسياسات في مجال التنوع البيولوجي وخدمات النظام الإيكولوجي، ومنظمة الأغذية والزراعة وهيئات المحميات ومجتمعات المستخدمين الأخرى – في وضع مجموعة من الأسئلة التعريفية وحالات الاستخدام القابل للإنجاز اللاتي في ضوئها يمكن قياس التقدم المحرز. ويجب أن تكون دقيقة ومفصلة بالقدر الكافي لكي توجه الأولويات نحو التخطيط التعاوني والتطوير والتنفيذ.

تنظيم الجهات المعنية

من الصعب فهم طبيعة الجهات المعنية لعمل التحالف، وذلك بسبب عدد الأنشطة ومهامها المتداخلة في كثير من الأحيان وتنفيذها من خلال برامج العمل على جداول زمنية مختلفة وعلى مستويات مختلفة من المسؤولية. وإذا لم يتم فهم هذه الصعوبة، هناك خطر كبير من التضارب غير المقصود أو ازدواجية الجهود. سوف ينسق المرفق العالمي لمعلومات التنوع البيولوجي (GBIF) تحليلا أوليا للشبكة - تحليل ذو حدود محددة النطاق بإحكام ووضوح - يسعى إلى تحديد أدوار ومسؤوليات وعلاقات المنظمات الرئيسية، وخاصة على الصعد العالمية والإقليمية والوطنية. وسوف يساعد هذا الجهد التحالف في تحديد الخدمات الحيوية التي يجب إنشاؤها أو استدامتها ويبين الفرص لتحسين التوافق أو التوحيد.

تبني مشروعات إثبات صحة المفاهيم

يتمثل أحد الأهداف الرئيسية للتحالف في تمكين الجهات المعنية من التجمع حول الاحتياجات المشتركة وتطوير مشروعات مستدامة لتقديم الأدوات والخدمات والنماذج والموارد التي تسهم في توفير نظام معرفي رقمي مترابط. وسيلزم وجود عمليات رسمية لتحديد أولويات هذه المشروعات وتطويرها وتقديمها والمحافظة عليها. وعلى المدى القصير، توجد قيمة في تحديد الأنشطة الحالية التي تدعم رؤية التحالف المفتوح والتي يمكن تبنيها كمشروعات إثبات صحة المفاهيم. ويمكن أن تقدم هذه المشروعات دروسا يمكن دمجها في نماذج الحوكمة المستقبلية، وإعطاء أمثلة مبكرة على التعاون القائم على التحالف في نفس الوقت. إننا نسعى للحصول على اقتراحات للأنشطة الحالية المناسبة كمرشحات لمشروعات إثبات صحة المفاهيم المبدئية، ليس في تطوير البرمجيات أو إدارة البيانات فحسب، بل في مجالات أخرى أيضًا مثل تعزيز القدرات وتخطيط الاستدامة.

Hobern D, Baptiste B, Copas K, Guralnick R, Hahn A, van Huis E, Kim E-S, McGeoch M, Naicker I, Navarro L, Noesgaard D, Price M, Rodrigues A, Schigel D, Sheffield CA & Wieczorek J (2019) Connecting data and expertise: a new alliance for biodiversity knowledge. Biodiversity Data Journal. doi:10.3897/BDJ.7.e33679
