# Supplementary material for: Connecting data and expertise: a new alliance for biodiversity knowledge
Source: Biodivers Data J. 2019 Mar 8;7:e33679. doi: 10.3897/BDJ.7.e33679 (PMC6420472; doi:10.3897/BDJ.7.e33679)
Supplement: Supplementary material 5 — Convocatoria de una alianza para el conocimiento de la biodiversidad [file bdj-07-e33679-s005.pdf]

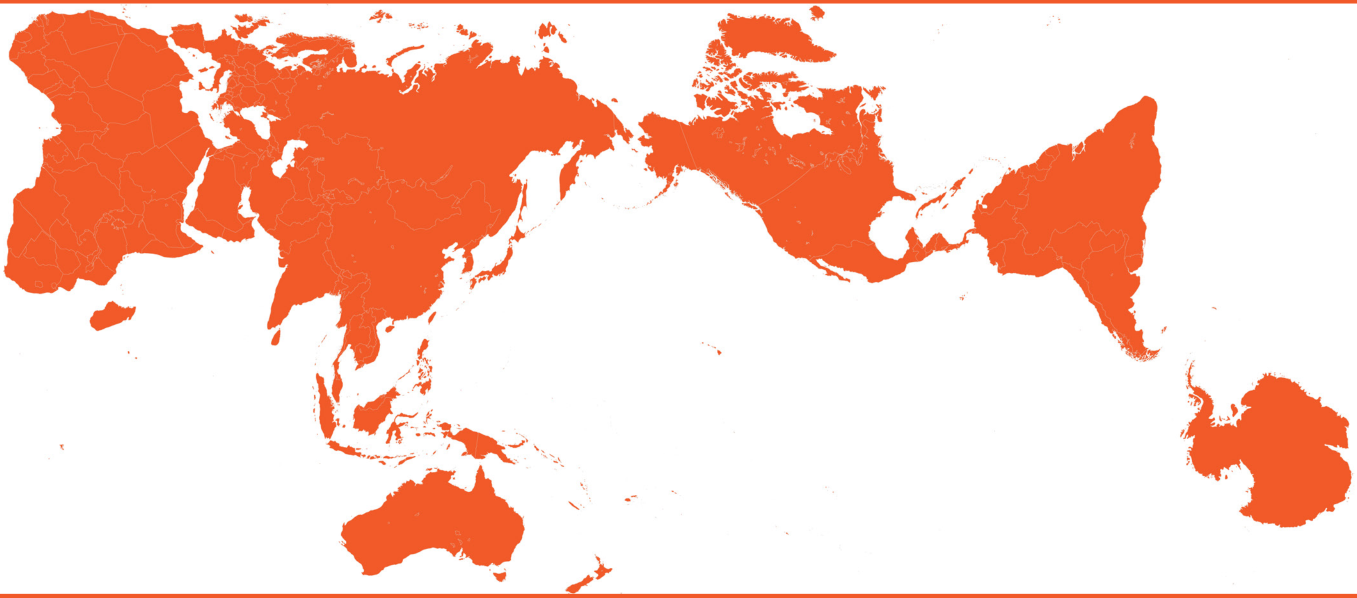

Convocatoria de **una alianza para el conocimiento de la biodiversidad**

VISIÓN

En las últimas dos décadas, se ha experimentado un gran avance en la digitalización del conocimiento histórico sobre Biodiversidad y en hacer libres y accesibles los datos de biodiversidad. La suma de esfuerzos ha traído consigo multitud de asociaciones y redes de trabajo internacionales, proyectos nacionales, regionales e institucionales, e incontables contribuciones individuales. Estas colaboraciones abarcan diversos ámbitos de investigación en diversidad biológica y medio ambiente, agencias gubernamentales y organizaciones no gubernamentales, ciencia ciudadana y el sector privado.

Pese a todo esto, los esfuerzos actuales continúan siendo ineficientes e inadecuados para abordar la necesidad global de contar con datos precisos sobre las especies que habitan la Tierra y sobre patrones y tendencias cambiantes en la Biodiversidad. Algunos retos significativos a abordar son:

- desequilibrios en los compromisos regionales sobre actividades relacionadas con la Informática de la Biodiversidad
- progreso desigual en la movilización y acceso a los datos
- falta de identificadores persistentes estables para los registros
- procesos redundantes e incompatibles para la limpieza e interpretación de datos
- ausencia de mecanismos funcionales que posibiliten a los expertos la depuración y mejora de datos

Habiendo reconocido la necesidad de un mayor alineamiento entre los esfuerzos a todos los niveles, la Infraestructura Mundial de Información en Biodiversidad (GBIF) convocó, en julio de 2018, la segunda Conferencia Mundial de Informática de la Biodiversidad (GBIC2) con el fin de proponer un mecanismo de coordinación que desarrolle los pasos a seguir en el ámbito de la Informática de la Biodiversidad. Los participantes en GBIC2 coincidieron en la necesidad de establecer una alianza global para el conocimiento de la biodiversidad, aprendiendo de ejemplos tales como **la Alianza Mundial para la Genómica y la Salud** (GA4GH) y las comunidades de software abierto como **la Fundación de Software Apache** (Apache Software Foundation). Estas iniciativas proporcionan modelos interesantes, con fuentes de financiación descentralizadas y gobernanzas independientes, que combinan recursos y desarrollan soluciones sostenibles para abordar necesidades comunes.

Supplementary Material 5: Convocatoria de una alianza para el conocimiento de la biodiversidad
doi:10.3897/BDJ.7.e33679.suppl5

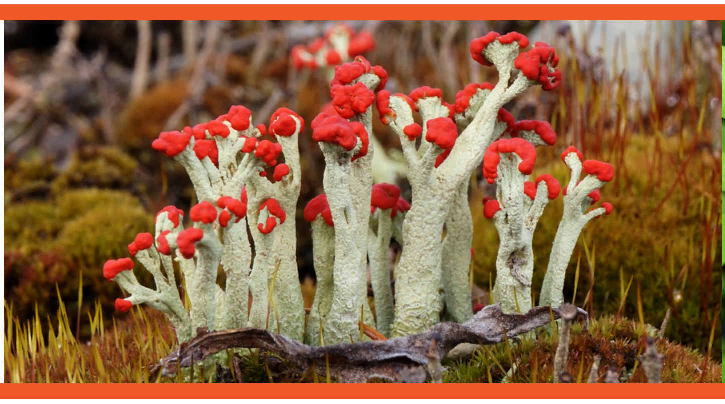

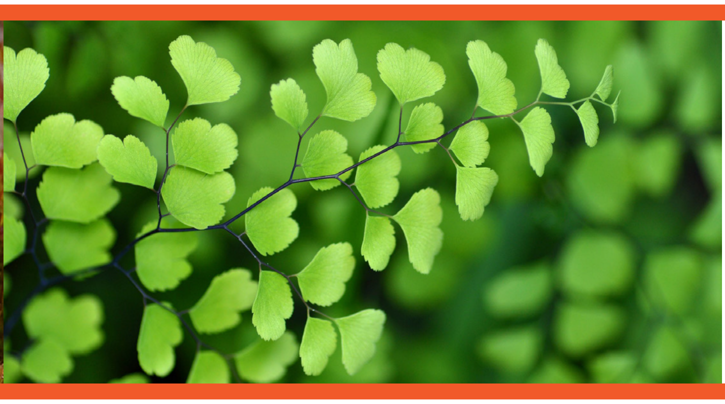

METAS

Los asistentes al GBIC2 propusieron la siguiente visión polifacética para definir las ambiciones de una alianza global para el conocimiento de la biodiversidad:

APOYO A LA CIENCIA Y LA PLANIFICACIÓN BASADA EN EVIDENCIAS CIENTÍFICAS

1. Ofrecer un entendimiento y conocimiento en Biodiversidad de manera que sirvan para apoyar las necesidades de la Ciencia y permitan que la Biodiversidad sea medida y evaluada correctamente para alcanzar metas sociales.
2. Servir como fundamento para investigaciones básicas en Biodiversidad y Ciencias de la Información que sirvan para el entendimiento humano de la funcionalidad y estado de los sistemas naturales.
3. Proporcionar una plataforma en continuo crecimiento para el entendimiento de la Biodiversidad mediante la conservación, desarrollo y mejora de los conocimientos existentes.

APOYO A LA CIENCIA ABIERTA Y LOS DATOS ABIERTOS

4. Eliminar las barreras al acceso libre y gratuito de los datos y adopción de los principios FAIR (**Wilkinson et al. 2016**) para los datos de Biodiversidad.
5. Describir todos los recursos de datos con metadatos de calidad que apoyen la reutilización presente y futura.
6. Asegurar que todos los recursos de datos son conservados en repositorios confiables estables y persistentes.
7. Permitir la depuración, anotación y mejora colaborativas de todos los datos por parte de profesionales relevantes y comunidades de expertos.
8. Permitir a los proveedores de conocimiento y experiencia mantener sus contribuciones completamente registradas, reconocidas y registradas.
9. Monitorear la procedencia y atribución de todos los recursos de información.

APOYO A LOS DATOS DE BIODIVERSIDAD ALTAMENTE CONECTADOS

10. Movilizar representaciones digitales estructuradas de fuentes de datos históricos, incluyendo colecciones de museos y literatura.
11. Asegurar que todas las nuevas observaciones y medidas sean accesibles en representaciones digitales estructuradas tan pronto como sea posible una vez se hayan tomado.
12. Permitir la combinación, consulta y análisis de diferentes tipos de información en biodiversidad (distribución, rasgos, genes, etc.) como un todo interconectado.
13. Trabajar con otras comunidades e infraestructuras de investigación para alcanzar interoperabilidad con observaciones de la Tierra, datos de ciencias sociales y otras fuentes de recursos

Image credits. p.1: AuthaGraph map of the world with political borders adapted from **Justin Kunimune**, licensed under CC BY-SA 4.0. ***p.2:*** *(left)* British soldier lichen (*Cladonia cristatella*), by Mike Lewinski via **iNaturalist**, licensed under CC BY 2.0; (*right*) Black maidenhair fern (*Adiantum capillus-veneris*) by Robby Deans via **iNaturalist**, licensed under CC BY-NC 4.0.

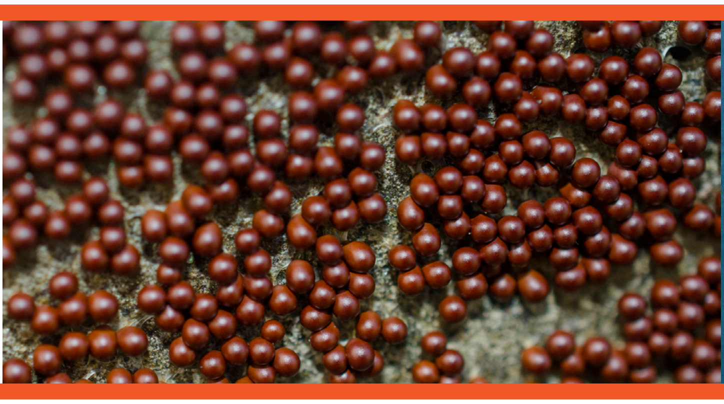

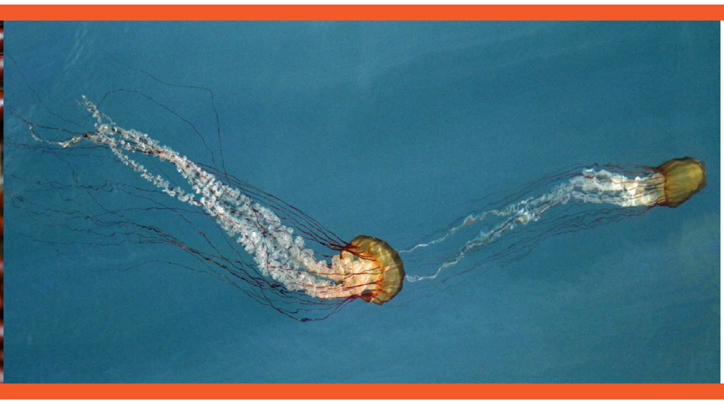

APOYO A LA COLABORACIÓN INTERNACIONAL

14. Abordar las necesidades de capacidades en torno a la informática de la biodiversidad en todas las regiones y sectores.
15. Asegurar financiación para mantener los servicios y componentes que la comunidad reconoce como elementos críticos de una infraestructura de conocimientos distribuidos.
16. Desarrollar enfoques flexibles y colaborativos para diseñar, construir y sostener todos los componentes de esta infraestructura de conocimientos distribuidos.
17. Permitir a los actores claves de cada país y región adoptar y beneficiarse de los avances en la infraestructura, herramientas, servicios, prácticas y capacidades.
18. Permitir la completa participación y colaboración con todos los grupos clave en todas las regiones y en todas las fases, desde la generación de datos hasta su análisis y aplicación.
19. Permitir la repatriación de datos que apoyen la Ciencia y la formulación de políticas en todos los países y regiones.
20. Asegurar el acceso efectivo, y el uso de los datos a todas las escalas, global, regional, nacional y local.
21. Reconocer y apoyar el papel de las inversiones regionales, nacionales y locales como componentes críticos y efectivos de una solución global.
22. Superar las barreras a la publicación y uso de datos derivadas de idiomas y contextos culturales.
23. Apoyar la implementación práctica de acuerdos internacionales con referencia al acceso a datos y que beneficien la publicación de éstos.

PRÓXIMOS PASOS

Se urge a todas las personas y entidades clave con interés en la producción, gestión, uso e integración de datos de la Biodiversidad mundial a contribuir al establecimiento de esta alianza global para el conocimiento de la biodiversidad colaborando con los siguientes procesos iniciales.

Further information, and opportunities to contribute to associated discussions, is maintained on the ***alliance*** website, **biodiversityinformatics.org**. Visita la pestaña *Discussions* de la web para contribuir a las discussions sobre cada une de estas cinco áreas. **Las contribuciones en otros idiomas diferentes al inglés son bienvenidas**.

AMPLIAR LA PARTICIPACIÓN

El taller, su informe, y este llamado a la acción han sido preparados para la comunidad global. Se pretende que los individuos e instituciones con interés en movilizar, mejorar, integrar o utilizar información de Biodiversidad muestren su apoyo mediante su firma y/o suscripción a posteriores actualizaciones en la página web de la ***alianza***, **biodiversityinformatics.org**.

p.3: *(left)* Insect-egg mold (*Leocarpus fragilis*) by Tim Sage via **iNaturalist**, licensed under CC BY-NC 4.0; (*right*) Pacific sea nettle (*Chrysaora fuscescens*) by Robin Agarwal via **iNaturalist**, licensed under CC BY-NC 4.0.

EVALUAR MODELOS

Se requiere un mayor esfuerzo para abordar las necesidades de esta compleja y diversa comunidad de actores clave, pero los modelos de otras alianzas, coaliciones y consorcios similares (como el meritocrático "Apache Way" utilizado por proyectos de herramientas informáticas de código abierto), pueden guiar y nutrir a enfoques de más largo plazo. También persisten preguntas importantes en torno a los tipos de membresía (individuales, institucionales o ambas).

ACLARAR EL ALCANCE Y LOS RESULTADOS PREVISTOS

Una mayor cooperación traerá, sin lugar a dudas, ventajas y eficiencias significativas en el ámbito de la Informática de la Biodiversidad, pero la meta final es generar un impacto en la Ciencia, la política y la sociedad. Los asistentes al GBIC2 propusieron integrar diversos actores clave –incluyendo grupos de investigación, servicios taxonómicos, el CBD, IPBES, FAO, entidades de conservación, y otras comunidades de usuarios– para desarrollar un conjunto de preguntas definitorias y casos de uso alcanzables contra los cuales medir el progreso. Éstos deberían ser lo suficientemente precisos y detallados para guiar las prioridades de planificación, desarrollo e implementación colaborativos.

IDENTIFICAR LAS PARTES INTERESADAS

Comprender el panorama de las partes interesadas en el trabajo de la alianza es complicado, debido al gran número de actividades, sus frecuentes misiones solapadas, y su implementación vía programas de trabajo en diferentes líneas temporales y escalas de responsabilidad. A menos que esta complejidad sea entendida, existe un elevado riesgo de conflicto involuntario o duplicidad de esfuerzo. GBIF coordinará una red de análisis inicial –con límites ajustados y claros –tratando de definir los roles, responsabilidades y relaciones de las principales organizaciones, particularmente a escalas global, regional y nacional. Este esfuerzo ayudará a la alianza a identificar servicios críticos que necesitan ser creados o sostenidos, y mostrar las oportunidades para un trabajo unificado y mejor alineado.

ADOPTAR PROYECTOS DE PRUEBA DE CONCEPTO

Una meta clave para la alianza es permitir a las partes interesadas converger en torno a necesidades compartidas e incubar proyectos sostenibles para ofrecer herramientas, servicios, modelos y recursos que contribuyan a la creación de un sistema de conocimientos digitales interconectados. Por ello, se requerirán procesos formales para priorizar, incubar, desarrollar y sostener tales proyectos. A corto plazo, resulta valioso identificar las actividades actuales que apoyan la visión de una alianza abierta y que pueden ser adaptados como proyectos de prueba de concepto. Tales proyectos pueden ofrecer lecciones que pueden ser incorporadas a futuros modelos de gobernanza, mientras que se proporcionan los primeros ejemplos de una cooperación basada en la alianza. Estamos buscando sugerencias sobre actividades existentes sostenibles como candidatas de los primeros proyectos de prueba de concepto, no únicamente en el desarrollo de herramientas informáticas o la gestión de datos, sino también en otras áreas como la mejora de capacidades y la planificación sostenible.

Hobern D, Baptiste B, Copas K, Guralnick R, Hahn A, van Huis E, Kim E-S, McGeoch M, Naicker I, Navarro L, Noesgaard D, Price M, Rodrigues A, Schigel D, Sheffield CA & Wieczorek J (2019) Connecting data and expertise: a new alliance for biodiversity knowledge. Biodiversity Data Journal. **doi:10.3897/BDJ.7.e33679**
