# Supplementary material for: Connecting data and expertise: a new alliance for biodiversity knowledge
Source: Biodivers Data J. 2019 Mar 8;7:e33679. doi: 10.3897/BDJ.7.e33679 (PMC6420472; doi:10.3897/BDJ.7.e33679)
Supplement: Supplementary material 6 — Biodibertsitatea ezagutzeko aliantza baterako deialdia [file bdj-07-e33679-s006.pdf]

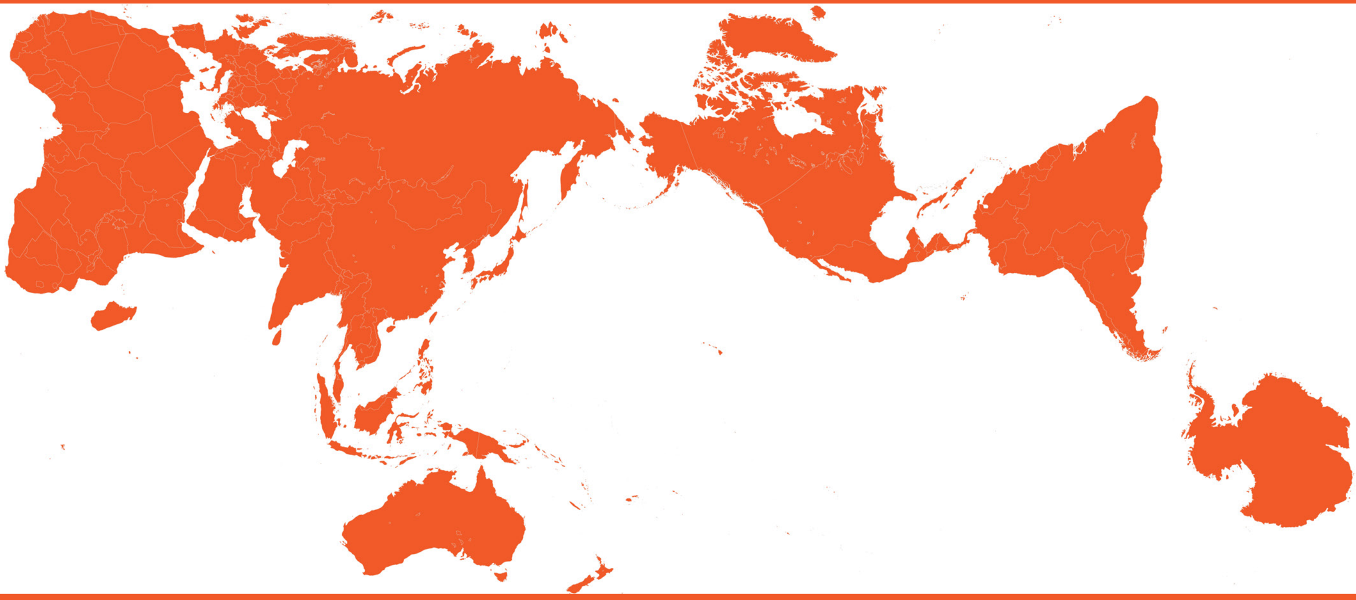

# Biodibertsitatea ezagutzeko aliantza baterako deialdia

## IKUSMEN

Azken bi hamarkadotan, asko aurreratu da Biodibertsitatearen ezagutza historikoaren digitalizazioan eta Biodibertsitate-datuetarako irispide librean. Ahaleginaren batuketak lankidetza ugari ekarri ditu, hala nola nazioarteko lan-elkarte eta -sareak, estatu-, eskualde- eta erakunde-mailako proiektuak eta banakako beste hainbat eta hainbat ekarpen. Lankidetza horiek arlo ezberdinetan eman dira: dibertsitate biologikoaren eta ingurumenaren ikerketak, gobernu-agentziak eta gobernuz kanpoko erakundeak, herritar-zientzia eta sektore pribatua.

Baina, hala ere, egindako ahalegin horiek guztiak ez dira nahikoa, eta egokiak ere ez, Biodibertsitateari buruz datu zehatzak izateko daukagun behar globalari erantzuteko: zenbat espezie bizi diren Lurrean eta zein diren Biodibertsitatearen patroiak eta aldatzeko joerak. Hona aurre egin beharreko erronka esanguratsu batzuk:

- eskualde-mailako desorekak Biodibertsitatearen Informatikari buruzko jardueretan, denek ez baitute konpromiso-maila bera.
- progresio ezberdinak datuak mugiarazteko eta haietara iristeko
- erregistroetarako etengabeko identifikatzaile egonkorren falta
- datuak garbitzeko eta interpretatzeko prozedura erredundante eta bateraezinak
- datuen garbiketa hobetu ahal izateko mekanismo funtzionalik ez adituentzat

Aitortuta maila guztietan egiten diren ahaleginak bateratu beharraren premia, Biodibertsitate Globalari buruzko Informazio Sistemak (GBIF) Biodibertsitatearen Informatikari buruzko Bigarren Mundu Konferentzia (GBIC2) deitu zuen 2018ko uztailean, helburu honekin: Biodibertsitatearen eremuan jarraitu beharreko urratsak garatzeko koordinazio-mekanismo bat proposatzea. GBIC2an parte hartu zuten guztiek ikusten zuten beharrezkoa zela Biodibertsitatea ezagutzen lagunduko duen aliantza global bat ezartzea, adibidetzat hartuz **Genomikarako eta Osasunerako Mundu Aliantza** (GA4GH) eta **Apache Softwarearen Fundazioa** bezalako software irekiko komunitateak. Ekimen horiek eredu interesgarriak dira, zeren eta finantzazio-iturri deszentralizatuak eta gobernantza independenteak baitituzte, eta premia partekatuei ekiteko baliabideak konbinatzen eta konponbide jasangarriak garatzen.

*Supplementary Material 6: Biodibertsitatea ezagutzeko aliantza baterako deialdia*
**doi:10.3897/BDJ.7.e33679.suppl6**

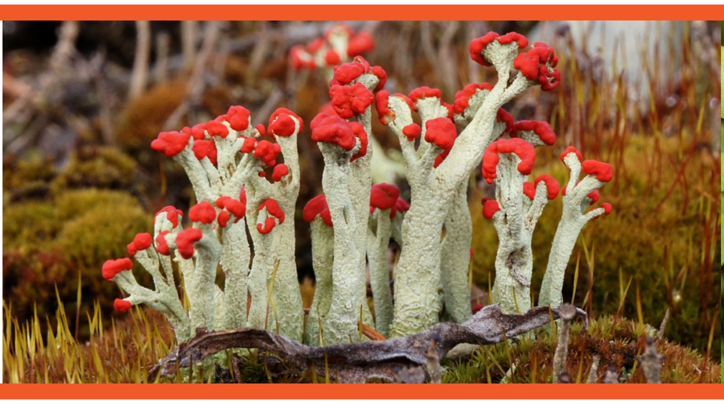

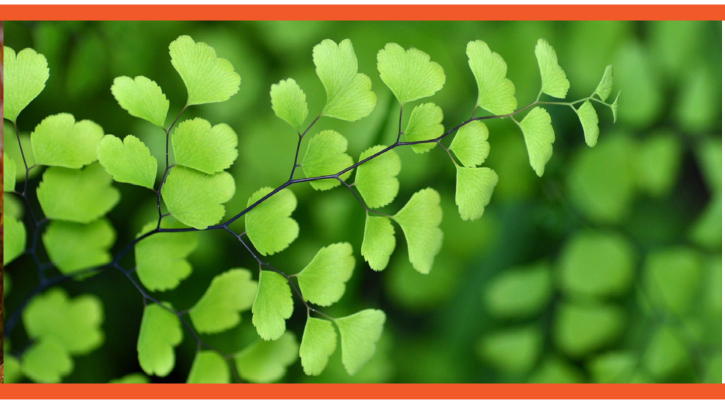

Lankidetza areagotuz, dauden datuen kudeaketa hobetuz eta datu-iturri berrien garapena koordinatuz, interkonektatutako ezagutza-base integratu bat sor daiteke, Biodibertsitatearen alderdi guztiei buruzkoa, eta modu irekian eta doan zabaldu informazio hori behar edo erabili nahi duen ororentzat. Horrelako sistema batekin, integratu ahal izango genituzke, etorkizun jasangarri bati begira, egungo ezagutza zientifikoa eta erabakiak hartzeko prozesu arrazional eta egokiak.

# XEDE PARTEKATUAK

GBIC2ko parte-hartzaileek ikuspen polifazetiko hau proposatu zuten Biodibertsitatearen ezagutzarako aliantza global baten anbizioak definitzeko:

## BABESA ZIENTZIARI ETA EBIDENTZIAN OINARRITUTAKO PLANGINTZARI

1. Biodibertsitateari buruzko ulermena eta ezagutza eskaintzea, Zientziaren premiei erantzuteko eta Biodibertsitatea gizarte-xedeetarako behar bezala neurtzea eta baloratzea ahalbidetzeko.
2. Biodibertsitatearen eta Informazio Zientzien arloko oinarrizko ikerketarako funtsa izatea, oinarrizko ikerketa horrek gizakiok natura-sistemen funtzionamendua eta egoera ulertzeko balio dezan.
3. Biodibertsitatearen ulermenean etengabe hazteko plataforma bat eskaintzea, gaur egungo ezagutza zaintzeko, eraikitzeko eta hobetzeko.

## BABESA DATU IREKIEI ETA ZIENTZIA IREKIARI

4. Datuetarako irispide libre eta dohainerako dauden trabak suntsitzea, eta Biodibertsitate-datuetarako FAIR printzipioetara atxikitzea (**Wilkinson et al. 2016**)
5. Datu-baliabide guztiak kalitatezko metadatuekin deskribatzea, gaur eta bihar berrerabili ahal izateko.
6. Datu-baliabide guztiak errepositorio fidagarri, egonkor eta iraunkorretan kontserbatzen direla bermatzea.
7. Profesional garrantzitsuek eta aditu-komunitateek datu guztiak elkarlaneko sistemaetan garbitzea, jasotzea eta hobetzea ahalbidetzea.
8. Ezagutza eta esperientzia eransten dutenek egiten dituzten ekarpenak erabat erregistratuta eta aitortuta edukitzea ahalbidetzea.
9. Informazio-baliabide guztien jatorria eta eskuduntza arakatzea

## BABESA OSO KONEKTATUTA DAUDEN BIODIBERTSITATE-DATUEI

10. Datu historikoen iturrien irudikapen digital egituratuak mugiaraztea, museotako bildumak eta literatura barne hartuta.
11. Behaketa eta neurketa berriak egin ondoren, horiek guztiak irudikapen digitaletan ahalik eta lasterren irisgarri egotea bermatzea.
12. Biodibertsitateari buruzko informazio mota ororen (banaketa, ezaugarriak, geneak eta abar) konbinaketa, kontsulta eta analisia ahalbidetzea, osotasun interkonektatua balitz bezala.
13. Beste ikerketa-komunitate eta -azpiegitura batzuekin lan egitea, elkarreragingarritasuna lortzeko Lurraren gaineko behaketen, gizarte-zientzien datuen eta bestelako baliabideen bidez.

***Image credits. p.1:*** AuthaGraph map of the world with political borders adapted from **Justin Kunimune**, licensed under CC BY-SA 4.0. ***p.2:*** *(left)* British soldier lichen (*Cladonia cristatella*), by Mike Lewinski via **iNaturalist**, licensed under CC BY 2.0; (*right*) Black maidenhair fern (*Adiantum capillus-veneris*) by Robby Deans via **iNaturalist**, licensed under CC BY-NC 4.0.

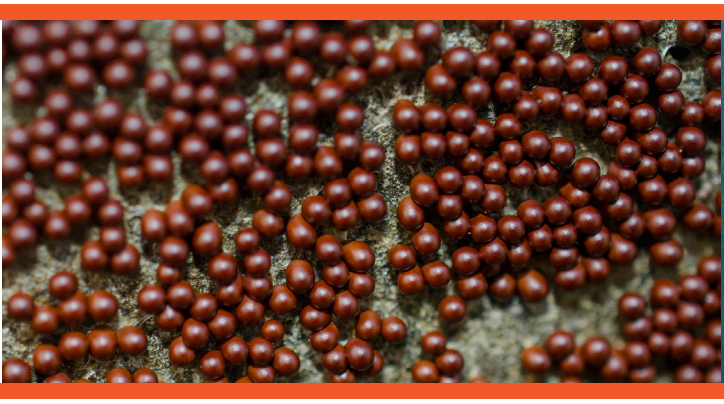

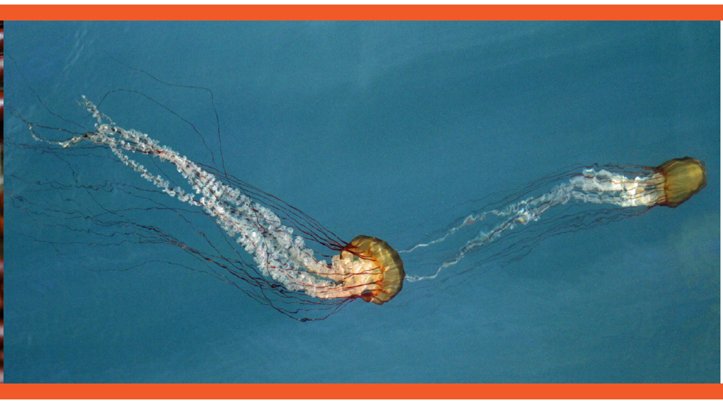

## BABESA NAZIOARTEKO LANKIDETZARI

14. Eskualde eta sektore guztietan jorratzea Biodibertsitatearen Informatikaren inguruko gaikuntza-premiak.
15. Komunitateak ezagutza-azpiegitura antolatu baten barruan elementu kritikotzat jotzen dituen zerbitzuei eta osagaiei eusteko finantzazioa ziurtatzea.
16. Ikuspegi malgu eta elkarlanekoak garatzea, ezagutza-azpiegitura antolatu horren osagai guztiak diseinatzeko, eraikitzeko eta mantentzeko.
17. Herrialde edo eskualde bakoitzeko interesdun alderdi guztiek azpiegituran, tresnetan, zerbitzuetan, jardueretan eta gaikuntzan egiten diren aurrerapenak hartzea eta horietatik etekina ateratzea ahalbidetzea.
18. Eskualde eta etapa guztietan ahalbidetzea agente-talde guztiek parte hartzea eta elkarlanean aritzea, datuak sortzetik hasi eta horiek aztertu eta ezarri arte.
19. Zientzia babesten duten datuak eta politiken formulazioa aberriratzea ahalbidetzea herrialde eta eskualde guztietan.
20. Edozein eskalatan bermatzea datuetarako irispidea eta horien erabilera eraginkorra: mundu-, eskualde-, estatu- eta toki-mailan.
21. Eskualdeko, estatuko eta tokiko inbertsioak irtenbide orokor baten barruan osagai kritikoak eta eraginkorrak direla aitortzea eta babestea.
22. Hizkuntzatik edo kulturatik eratorritako datuen erabilerak eta trukeak dauzkaten oztopoak gainditzea.
23. Datuetarako irispidea eta datuen argitalpena babesten dituzten nazioarteko akordioen inplementazio praktikoa babestea.

# HURRENGO URRATSAK

Munduko Biodibertsitate-datuak ekoizteko, kudeatzeko eta integratzeko interesa duten pertsona eta entitate guztiei dei egiten zaie Biodibertsitatearen ezagutzarako aliantza global hau ezartzen lagun dezaten beren lankidetza eskainiz hasiera-prozesuetan.

Informazio gehiagorako eta elkartutako eztabaidetara bideratzeko aukera, aliatuen web orrian mantentzen da, **biodiversityinformatics.org**. Bisita ezazu **webeko Discussions fitxa**, bost arlo hauetako eztabaidetan parte hartu ahal izateko. **Ingelesez ez beste hizkuntzetan ere egin daitezke ekarpenak**.

## PARTE-HARTZEA ZABALTZEA

Tailerra, horri buruzko txostena eta deialdi hau komunitate globalarentzat prestatu dira. Helburua da Biodibertsitatearen inguruko informazioa mugiarazteko, hobetzeko, integratzeko edo erabiltzeko interesa duten banakako eta erakunde guztiek beren babesa erakustea, aliantza hau sinatuz edo aliantzaren web orrian gerora egingo diren eguneraketetara harpidetuz.

***p.3:*** *(left)* Insect-egg mold (*Leocarpus fragilis*) by Tim Sage via **iNaturalist**, licensed under CC BY-NC 4.0; (*right*) Pacific sea nettle (*Chrysaora fuscescens*) by Robin Agarwal via **iNaturalist**, licensed under CC BY-NC 4.0.

## EREDUAK EBALUATZEA

Ahalegin handiagoa egin behar da interesdun alderdiek osatzen duten komunitate konplexu eta askotariko honen premiei ekiteko; horretan gidari izan daitezke eta epe luzeagorako ikuspegiak eskaini ditzakete beste aliantza-, koalizio- eta partzuergo-eredu batzuk, adibidez, "Apache Way" meritukratikoa, kode irekiko tresna informatikoen proiektuetan erabilia. Hortxe jarraitzen dute, halaber, kide-motei buruzko galdera garrantzitsuek ere (banakakoak, erakundeak edo biak).

## ARGITZEA AURREIKUSITAKO IRISMENA ETA EMAITZAK

Lankidetza handiago batek abantaila eta ondorio esanguratsuak ekarriko ditu, zalantzarik gabe, Biodibertsitatearen Informatikaren eremuan, baina azken xedea da inpaktu bat eragitea Zientzian, Politikan eta gizartean. GBIC2ko parte-hartzaileek interesdun alderdi ezberdinak integratzea proposatu zuten –barruan sartuta ikerketa-taldeak, zerbitzu taxonomikoak, CBD, IPBES. FAO, kontserbazio-entitateak eta beste erabiltzaile-komunitate batzuk– galdera definitzaileen multzo bat eta erabilera-kasu irisgarri batzuk garatuko ditu, eta aurrerapena haien kontra neurtu ahal izateko – galdera definitzaileen multzoa eta erabilera-kasuak nahikoa zehatzak izan behar dute, elkarlanean oinarritutako plangintza, garapen eta inplementazio lehenetsiak gidatzeko.

## INTERESDUN ALDERDIAK IDENTIFIKATZEA

Zaila da ulertzea zein den aliantzaren lanean interesa duten alderdien egoera: jarduera asko egiten dituzte, misio gainjarriak dituzte askotan, eta lan-programen bidez inplementatzen dituzten jarduerok denbora-lerro eta erantzukizun-eskala ezberdinetan egiten dituzte. Ez bada konplexutasun hori ulertzen, arrisku handia dago nahigabeko gatazkak eragiteko, edo ahaleginak bikoizteko. GBIFk hasierako azterketa-sare bat koordinatuko du –muga zehatz eta argiekin–, eta saiatuko da rolak, ardurak eta erakunde nagusien zerrenda definitzen, batez ere mundu-, eskualde-, estatu- eta toki-mailakoak. Ahalegin horri esker, sortu eta sostengatu behar diren zerbitzu kritikoak identifikatuko ditu aliantzak, eta lana modu batuago eta lotuago batean egiteko aukerak erakutsi.

## KONTZEPTU-PROBARAKO PROIEKTUAK ERABILTZEA

Aliantzarentzat, funtsezko xede bat da interesdun-alderdi guztiek premia partekatuen inguruan bat egitea eta proiektu jasangarriak hastea, ezagutza digital interkonektatuen sistema bat sortzen laguntzen duten tresnak, zerbitzuak, ereduak eta baliabideak eskaintzeko. Horregatik, proiektu horiek lehenesteko, hasteko, garatzeko eta sostengatzeko prozesu formalak beharko dira. Epe motzera, baliagarria da identifikatzea zein diren gaur egun aliantza ireki baten ikuspegia babesten duten jarduerak, kontzeptu-probarako proiektu gisa egokitu ahal izateko. Proiektu horiek, aliantzan oinarritutako lehen lankidetza-adibideak izateaz gain, etorkizuneko gobernantza-ereduetan aplikagarri izan daitezkeen irakaspenak eskaini ditzakete. Iradokizunen bila gabiltza, kontzeptu-proben lehen proiektuetarako dagoeneko egiten diren jarduera jasangarriei buruz, eta ez bakarrik tresna informatikoen garapenari edo datu-kudeaketari dagokienez, baizik eta gaikuntzen hobekuntzaren eta plangintza jasangarriaren arloei dagokienez ere bai.

Hobern D, Baptiste B, Copas K, Guralnick R, Hahn A, van Huis E, Kim E-S, McGeoch M, Naicker I, Navarro L, Noesgaard D, Price M, Rodrigues A, Schigel D, Sheffield CA & Wieczorek J (2019) Connecting data and expertise: a new alliance for biodiversity knowledge. Biodiversity Data Journal. **doi:10.3897/BDJ.7.e33679**
