# Supplementary material for: Connecting data and expertise: a new alliance for biodiversity knowledge
Source: Biodivers Data J. 2019 Mar 8;7:e33679. doi: 10.3897/BDJ.7.e33679 (PMC6420472; doi:10.3897/BDJ.7.e33679)
Supplement: Supplementary material 7 — Appel à une alliance pour la connaissance sur la biodiversité [file bdj-07-e33679-s007.pdf]

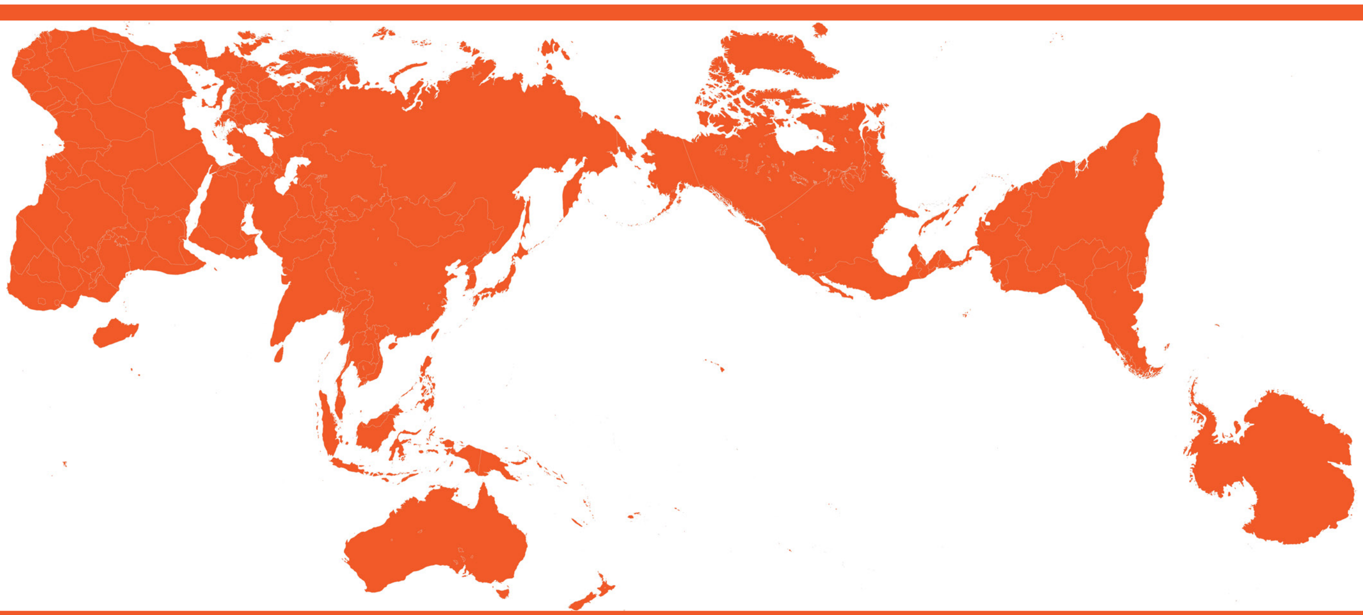

Appel à
une alliance pour la connaissance sur la biodiversité

VISION

Dans les deux dernières décennies, de grandes avancées ont été observées en ce qui concerne la numérisation des connaissances historiques en biodiversité, ainsi que l'accessibilité des données de biodiversité sous forme de données ouvertes. Des efforts conjoints ont permis la naissance de partenariats et réseaux internationaux, des projets et investissements nationaux, régionaux et institutionnels, ainsi que d'innombrables contributions individuelles. Ces collaborations englobent des domaines de recherche variés en biologie et en environnement, des agences gouvernementales et des organisations non-gouvernementales, des projets de science citoyenne et des entreprises commerciales.

Néanmoins, les efforts actuels restent inefficaces et inadéquats pour répondre au besoin global de données précises sur les espèces du monde entier, ainsi que sur les modèles et tendances de changements en biodiversité. Les défis les plus critiques incluent :

- un déséquilibre dans l'engagement régional pour les activités en informatique de la biodiversité
- des progrès inégaux en ce qui concerne la mobilisation et le partage des données de biodiversité
- l'absence d'identifiants uniques et stables pour les occurrences
- des protocoles redondants et incompatibles pour le nettoyage et l'interprétation des données
- l'absence de mécanisme fonctionnel afin que des experts puissent vérifier et améliorer les données

En reconnaissant le besoin pour une meilleure coordination entre les différents efforts à tous les niveaux, le Système Mondial d'Information sur la Biodiversité (GBIF) a organisé la seconde Conférence Globale en Informatique de la Biodiversité (GBIC2) en juillet 2018 dans le but de proposer un mécanisme de coordination afin de développer des feuilles de route communes en informatique de la biodiversité. Les participants au GBIC2 sont arrivés à un consensus par rapport à la nécessité d'une alliance globale pour les connaissances en biodiversité, en se basant sur des exemples tels que **l'Alliance Globale pour la Génomique et la Santé** (GA4GH) et les communautés de logiciels ouverts maintenues par **la Fondation des Logiciels Apache** (Apache Software Foundation). Ces initiatives fournissent des modèles pour de multiples intervenants ayant des sources de financement décentralisées et une gouvernance indépendante, afin de combiner les ressources et de développer des solutions durables qui répondent aux besoins de tous.

Supplementary Material 7: Appel à une alliance pour la connaissance sur la biodiversité
doi:10.3897/BDJ.7.e33679.suppl7

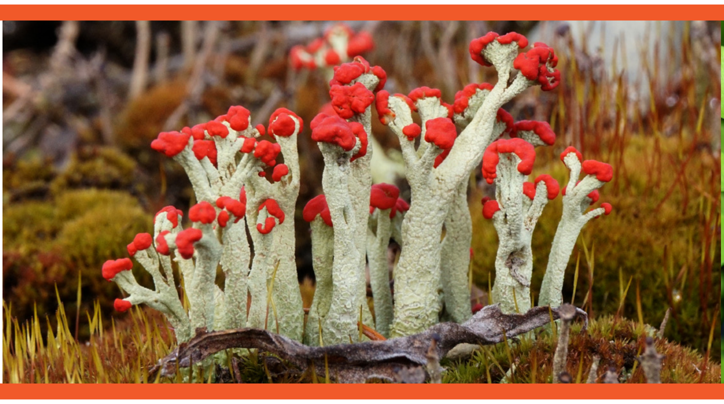

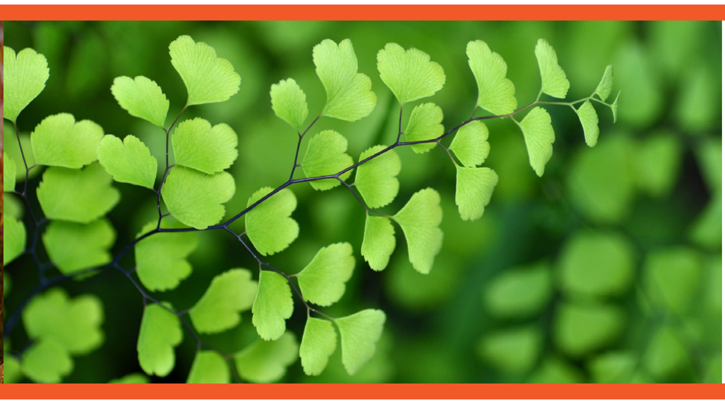

Une meilleure coopération, une amélioration de la gestion des données existantes et le développement coordonné de nouvelles sources de données pourrait engendrer la création d'une base de connaissance intégrée et inter-reliée entre tous les aspects de biodiversité—une base librement et ouvertement diffusée à quiconque en aurait besoin ou serait intéressé à utiliser. Mettre en place un tel système nous permettrait d'intégrer les connaissances scientifiques actuelles en biodiversité au sein de processus décisionnels rationnels et opportuns soutenant un futur durable.

AMBITIONS

Les participants au GBIC2 proposent la vision multi-facette suivante afin de définir les ambitions pour une alliance pour les connaissances en biodiversité :

SOUTIEN À LA SCIENCE ET À LA PLANIFICATION BASÉE SUR LES PREUVES

1. Fournir connaissance et compréhension sur la biodiversité sous des formes qui soutiennent les pré-requis de la recherche et qui permettent de mesurer et d'évaluer correctement la biodiversité dans des buts sociétaux.
2. Servir de fondation pour la recherche en biodiversité et en science de l'information, et qu'elle soit utile à notre compréhension du fonctionnement et de l'état des systèmes naturels.
3. Fournir une plateforme permettant une amélioration constante de notre compréhension de la biodiversité en préservant, en s'appuyant sur et en améliorant les connaissances actuelles.

SOUTIEN POUR LES DONNÉES ET LA SCIENCE OUVERTES

4. Supprimer les barrières au partage de données ouvertes et adopter les principes de partage FAIR (**Wilkinson et al. 2016**) pour les données de biodiversité
5. Décrire tous les jeux de données à l'aide de métadonnées riches, supportant ainsi la réutilisation présente et future.
6. S'assurer que tous les jeux de données sont conservés dans des dépôts stables et persistants
7. Permettre la conservation collaborative, l'annotation et l'amélioration de toutes les données, par n'importe quels experts et communautés d'experts pertinents
8. Permettre à tous les fournisseurs de connaissances ou d'expertise de voir leurs contributions enregistrées, reconnues et créditées
9. Faire un suivi de la provenance et de l'attribution de toutes les sources d'information

SOUTIEN POUR DES DONNÉES DE BIODIVERSITÉ HYPER-CONNECTÉES

10. Mobiliser des formats numériques structurés de sources de données historiques, incluant les collections muséales et la littérature
11. S'assurer que toutes les nouvelles observations et mesures sont accessibles sous forme de données numériques structurées aussi rapidement que possible après la capture des données
12. Permettre la mise en commun, la recherche et l'analyse des différentes classes d'information sur la biodiversité (distribution, traits, gènes, etc.) en tant qu'éléments interconnectés
13. Travailler avec d'autres communautés de recherche et infrastructures afin d'arriver à une inter-opérabilité avec les mesures environnementales terrestres, les sciences sociales et d'autres ressources

Image credits. p.1: AuthaGraph map of the world with political borders adapted from **Justin Kunimune**, licensed under CC BY-SA 4.0. ***p.2:*** *(left*) British soldier lichen (*Cladonia cristatella*), by Mike Lewinski via **iNaturalist**, licensed under CC BY 2.0; (*right*) Black maidenhair fern (*Adiantum capillus-veneris*) by Robby Deans via **iNaturalist**, licensed under CC BY-NC 4.0.

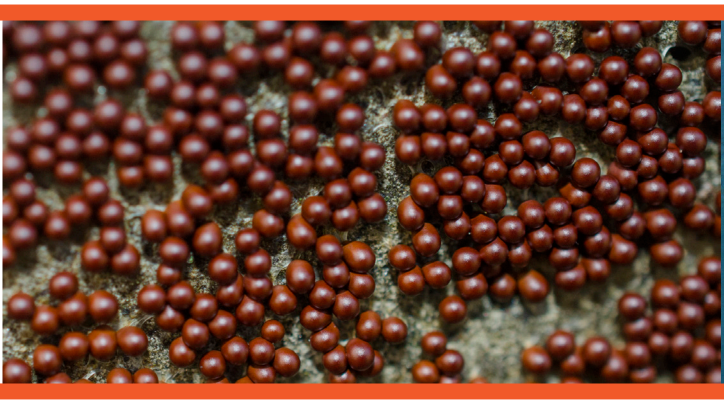

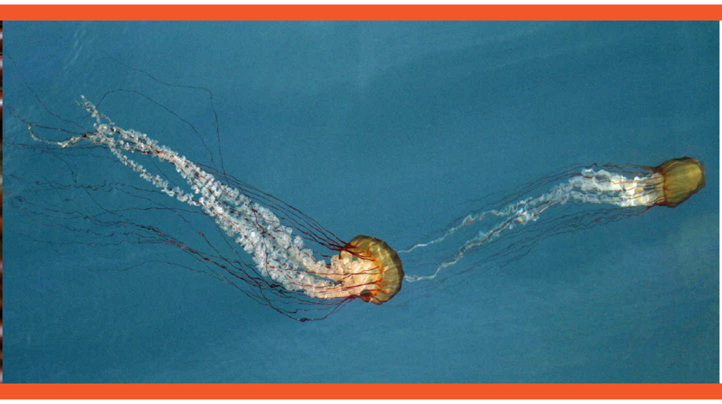

SOUTIEN POUR LA COLLABORATION INTERNATIONALE

14. Répondre aux besoins liés à l'informatique de la biodiversité dans toutes les régions et tous les secteurs
15. Sécuriser du financement afin de maintenir les services et les composantes que la communauté reconnaît comme des éléments primordiaux au sein d'une infrastructure de connaissances partagées
16. Développer des approches flexibles et collaboratives afin de concevoir, construire et maintenir toutes les composantes de cette infrastructure de connaissances partagées
17. Permettre aux intervenants de tout pays et région d'adopter et de bénéficier des avancées en terme d'infrastructure, d'outils, de services, de pratiques et de capacité
18. Permettre la participation complète et la collaboration avec tous les groupes d'intervenants dans toutes les régions à toutes les étapes, depuis la production de données à l'analyse de celles-ci et les applications qui en découlent
19. Permettre le rapatriement des données soutenant la science et les processus décisionnels
20. S'assurer d'un accès efficace aux données, ainsi que leur l'utilisation, à tous les niveaux - international, régional, national et local
21. Reconnaître et supporter le rôle des investissements régionaux, nationaux et locaux comme des composantes importantes et efficaces dans un contexte international
22. Surmonter les barrières limitant le partage ou l'utilisation des données en raison de la langue ou de la culture
23. Supporter la mise en oeuvre pratique des accords internationaux par rapport à l'accès et au partage des bénéfices

LES PROCHAINES ÉTAPES

Tous les intervenants intéressés dans la production, la gestion, l'utilisation ou l'intégration de données à propos de la biodiversité mondiale sont invités à contribuer à l'établissement de l'alliance pour les connaissances en biodiversité telle que proposée, en donnant leur avis sur cette proposition initiale.

De plus amples informations, et des opportunités de contribution aux discussions associées, seront disponibles sur le site Internet de l'alliance, **biodiversityinformatics.org**. Visiter la page "Discussions" sur le site Internet afin de contribuer aux **discussions sur chacun de ces cinq thèmes**. **Les contributions dans d'autres langues que l'anglais sont les bienvenues**.

AUGMENTER LA PARTICIPATION

L'atelier technique, son compte-rendu et cet appel à actions ont été préparés pour la communauté internationale. Les personnes et institutions intéressées par la mobilisation, l'amélioration, l'intégration et l'utilisation d'informations sur la biodiversité sont invitées à signaler leur soutien en s'inscrivant et/ou en s'abonnant au site de l'alliance (**biodiversityinformatics.org)** pour être informées des futures développements.

p.3: (*left*) Insect-egg mold (*Leocarpus fragilis*) by Tim Sage via **iNaturalist**, licensed under CC BY-NC 4.0; (*right*) Pacific sea nettle (*Chrysaora fuscescens*) by Robin Agarwal via **iNaturalist**, licensed under CC BY-NC 4.0.

ÉVALUER LES MODÈLES

Des actions supplémentaires sont nécessaires pour répondre aux besoins de cette communauté de décideurs diverse et complexe. Des modèles issus d'alliances, coalitions et consortiums similaires (comme "Apache Way" basé sur le mérite, utilisé par de nombreux projets de logiciels open-source) peuvent guider et fournir des informations pour des approches à plus long terme. Des questions importantes restent en suspens concernant les conditions d'adhésion (personnelle, institutionnelle, ou les deux).

CLARIFIER LE PÉRIMÈTRE ET CIBLER LES RÉSULTATS

Une meilleure coopération apportera sans nul doute des avantages significatifs et une plus grande efficacité dans le domaine de l'informatique de la biodiversité, mais l'objectif ultime est d'avoir un impact sur la science, les politiques publiques et la société. Les participants au GBIC2 ont proposé de solliciter des intervenants variés — incluant des groupes de recherche, des systèmes d'information taxonomiques, la CDB, l'IPBES, la FAO, des organismes de conservation, et d'autres communautés d'utilisateurs— afin d'établir une gamme de problématiques majeures et de cas d'utilisation réalisables sur la base desquels évaluer les progrès effectués. Ceux-ci devraient être suffisamment précis et détaillés pour orienter les priorités en termes de planification collaborative, développements et implémentation.

IDENTIFIER LES DÉCIDEURS

Le paysage des décideurs est difficile à appréhender dans le cadre des travaux de l'alliance, en raison du nombre d'activités, de leurs missions qui se recoupent, et de leur implémentation via des programmes de travail à différentes échelles de temps et de responsabilité. Si cette complexité n'est pas prise en compte, il y a un fort risque de conflits involontaires ou de duplication d'efforts. Le GBIF coordonnera une analyse initiale du réseau—avec des limites nettes et clairement évaluées— afin de définir les rôles, responsabilités et relations des principales organisations, en particulier à l'échelle internationale, régionale et nationale. Ce travail permettra à l'alliance d'identifier les services indispensables qui devront être créés ou maintenus, et d'indiquer les opportunités pour mieux s'accorder ou s'unifier.

ADOPTER DES PROJETS "PREUVE DE CONCEPT"

Un des objectifs principaux de l'alliance est de permettre aux décideurs de se rassembler autour de besoins communs et de développer des projets durables pour produire des outils, services, modèles et ressources qui contribuent à faire fonctionner un système interconnecté de connaissances numériques. Des processus formels seront requis afin de prioriser, incuber, produire et maintenir de tels projets. A court terme, il est intéressant d'identifier les activités en cours qui soutiennent la vision pour une alliance ouverte et qui peuvent être adoptées comme preuves de concept. De tels projets offrent des enseignements qui peuvent être incorporés dans de futurs modèles de gouvernance, tout en donnant des exemples précoces de coopération au sein de l'alliance. Nous recherchons des suggestions d'activités existantes qui conviendraient comme preuves de concept, non seulement en développement logiciel ou en gestion de données, mais aussi dans d'autres domaines tels que l'amélioration des capacités et la planification durable.

Hobern D, Baptiste B, Copas K, Guralnick R, Hahn A, van Huis E, Kim E-S, McGeoch M, Naicker I, Navarro L, Noesgaard D, Price M, Rodrigues A, Schigel D, Sheffield CA & Wieczorek J (2019) Connecting data and expertise: a new alliance for biodiversity knowledge. Biodiversity Data Journal. **doi:10.3897/BDJ.7.e33679**
