# Supplementary material for: Connecting data and expertise: a new alliance for biodiversity knowledge
Source: Biodivers Data J. 2019 Mar 8;7:e33679. doi: 10.3897/BDJ.7.e33679 (PMC6420472; doi:10.3897/BDJ.7.e33679)
Supplement: Supplementary material 8 — 生物多様性の知識のための連携協力の要請 [file bdj-07-e33679-s008.pdf]

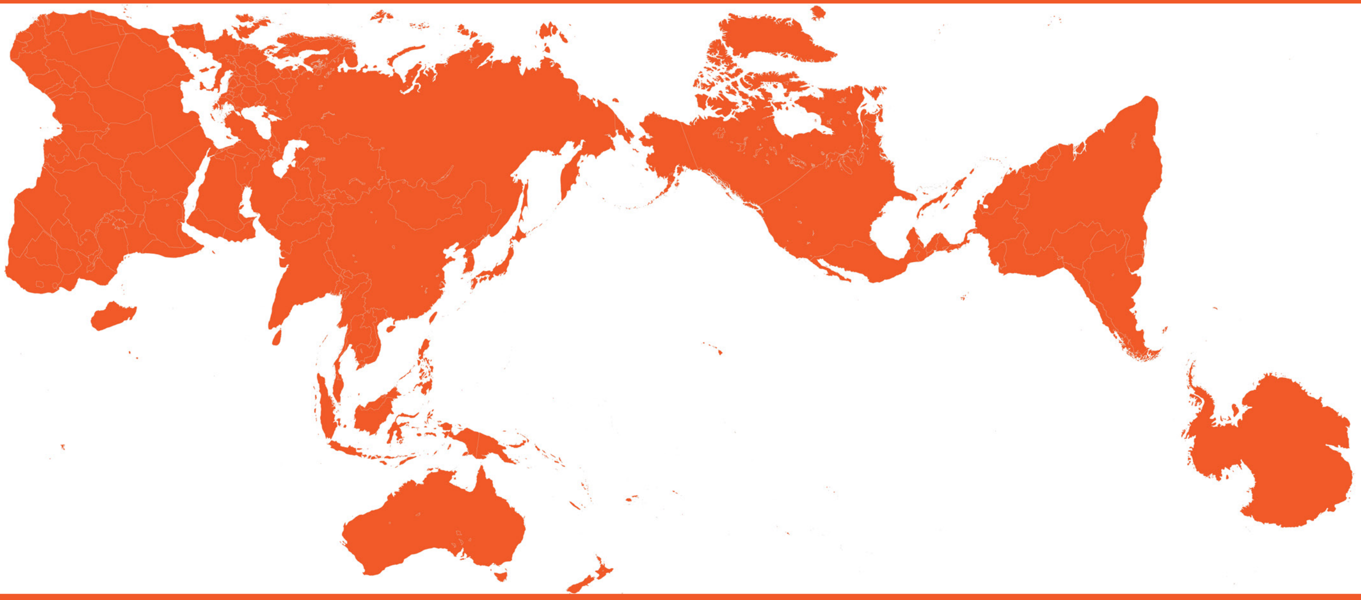

生物多様性の知識のための連携協力の要請

ビジョン

生物多様性に関する知識をデジタル化し、そのデータをフリーでオープンにアクセスできるようにすることに関して、この20年の間に大きな進展がありました。連携の努力によって、国際パートナーシップ・ネットワーク、国・地域・機関のプロジェクトと資金提供、数えきれないほどの個人による貢献がここに集結しました。この協働は様々な生物多様性および環境研究に関わる団体、政府機関、非政府機関、市民科学、民間企業の垣根を超えて実現しました。

しかしながら、世界の生物種や生物多様性の変動パターンおよび傾向に関する正確なデータを求める世界のニーズに対応するには、現状の努力では不十分かつ不適当です。次のような点において、変革が望まれます。

- 生物多様性情報学に関する活動における地域ごとの取り組みの格差
- データを使える状態にし共有することについての進捗状況の違い
- データレコードに対する永続的で安定したIDの欠如
- データクリーニングおよびデータ解釈の過程で生じる無駄と矛盾
- 知識のある専門家がデータの改善およびキュレーションを行うための機能的な仕組みの欠落

あらゆる規模でなされている努力を（格差なく）横並びにする必要性を認識し、地球規模生物多様性情報機構（GBIF）は、2018年7月に第二回地球規模生物多様性情報会議（GBIC2）を開催しました。これは、生物多様性情報学のロードマップを共有し、開発するための調整機構を提案するために開催されたものです。GBIC2の参加者は、Global Alliance for Genomics and Health (GA4GH)やApache Software Foundationに属するオープンソフトウェアコミュニティなどの事例に学び、生物多様性の知識のための国際連携協力の必要性について合意しました。これらの団体は、（中央集権的ではなく）分散型の資金調達と独立した管理体制をもつ利害関係者で構成され、リソースを統合することで共通のニーズを持続可能な方法で解決しようとする組織のお手本です。

連携の強化、現有データの管理方法の改善、そして新たな情報源の開拓を進めることで、情報が統合され、互いにリンクし、生物多様性のあらゆる側面で使える知識ベースが作成できます。これはフリーでオープンな情報として、必要とする人、興味を持った人なら誰でも利用できるものです。そのようなシステムを実現することで、持続可能な未来を支える合理的で時宜を得た意思決定プロセスに生物多様性に関する最新の科学的知見を取り込むことができるようになります。

Supplementary Material 8: 生物多様性の知識のための連携協力の要請
doi:10.3897/BDJ.7.e33679.suppl8

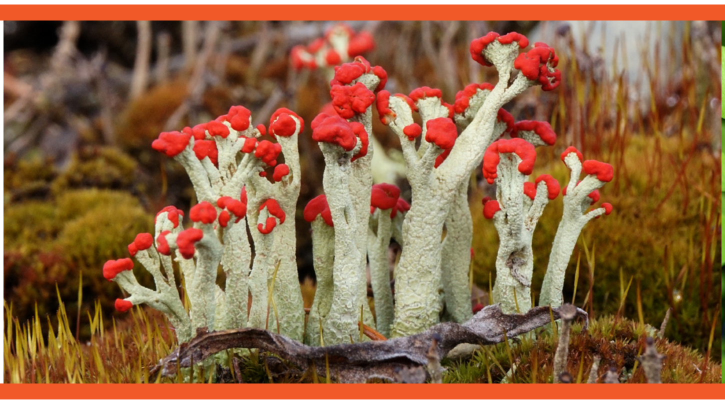 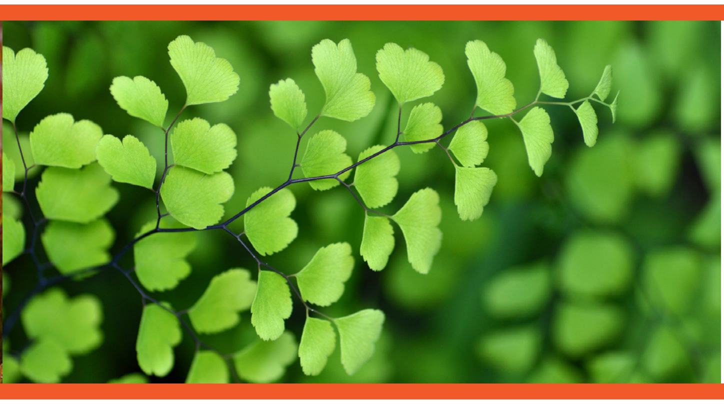

野心

GBIC2の参加者は、生物多様性の知識のための連携協力を要請するねらいを明確にするため、以下の多面的なビジョンを提案しました。

科学と証拠に基づく計画立案の支援

1. 生物多様性の知識および知見を提供する上で、研究において重要な要件を満たした形式であり、かつ社会の目指すゴールに対して生物多様性が正しく測定・評価できる形式であること。
2. 自然の体系の機能や状態に関する知見が得られる生物多様性情報科学の基礎研究の基盤を提供すること。
3. 現有の知識を保存し、それに基づいて改善することによって、生物多様性の知見をさらに広げるプラットフォームを提供すること。

オープンデータとオープンサイエンスをサポートする

4. 生物多様性データの公開において、フリーでオープンなデータ共有に対する障壁を取り除き、FAIR原則(Wilkinson et al. 2016)にしたがうこと。
5. すべてのデータリソースは、現在および将来の再利用のため、豊富なメタデータを伴って記述すること。
6. すべてのデータリソースは、安定して、永続的で、信頼できるリポジトリに必ず保管されるようにすること。
7. 関係した専門家や専門家のコミュニティが協同作業によるデータのキュレーション、アノーテーションおよび改善を行えるようにすること。
8. 専門的知識の提供に貢献したすべての人に対して、その貢献が完全に記録され、謝辞に述べられ、クレジットされるようにすること。
9. すべての情報のソースの出所と帰属が追跡可能であること。

高度に連結する生物多様性データのサポート

10. 博物館コレクションや文献など歴史的なデータソースの構造化されたデジタル情報を利用できるように集約すること。
11. 新規の観察・測定情報について取得後可能な限り早くに構造化されたデジタル情報としてアクセスできるようにすること。
12. 異なるクラスの生物多様性情報（分布・形質・遺伝子など）を全体に相互連結した形で組み合わせたり、検索したり、解析したりできるようにすること。
13. 他の研究コミュニティやインフラとの連携により、地球観測、社会科学的データ、その他のリソースとの相互運用性を実現すること。

Image credits. p.1: AuthaGraph map of the world with political borders adapted from **Justin Kunimune**, licensed under CC BY-SA 4.0. ***p.2:*** *(left)* British soldier lichen (*Cladonia cristatella*), by Mike Lewinski via **iNaturalist**, licensed under CC BY 2.0; (*right*) Black maidenhair fern (*Adiantum capillus-veneris*) by Robby Deans via **iNaturalist**, licensed under CC BY-NC 4.0.

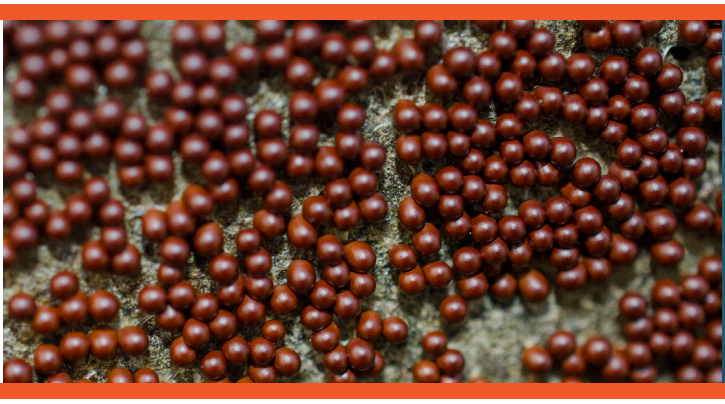 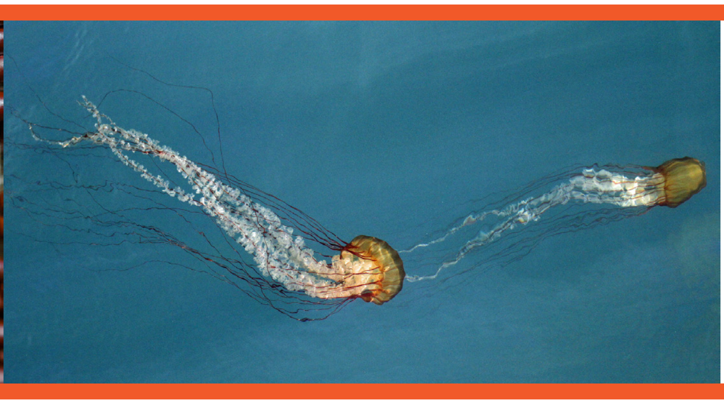

国際的なコラボレーションのサポート

14. すべての地域とすべてのセクターで、生物多様性情報学に関する能力強化のニーズを解決すること。
15. 提供されている知識インフラのうち、コミュニティが不可欠な要素として認識する
16. 前述の知識インフラを構成するすべてのコンポーネントをデザインし、構築し、維持する上で、柔軟かつ協働可能なアプローチを考案すること。
17. インフラ・ツール・サービスの進歩、事例の積み重ね、能力の向上がすべての国・地域の関係者に行き渡り、その利益を享受できること。
18. データをつくる段階から解析や応用に至る全工程で、すべての地域のすべての関係者グループが参加でき、協力し合えるようにすること。
19. すべての国・地域において科学および政策決定の土台となるデータのレパトリエーション（原産国にデータを受け渡すこと）が可能であること。
20. すべてのスケール（世界、地域、国、地方）でデータへの効果的なアクセスと利用が保証されること。
21. 国際的なソリューションにおいて不可欠で効果的な要素である地域・国・地方レベルでの投資に対して謝意と支持を表明すること。
22. 言語・文化の壁を超えてデータが共有や使用がなされること。
23. に鑑み、国際的な合意の実行をサポートすること。

次のステップ

世界の生物多様性データの作成、管理、使用、統合に興味をもつ関係者の皆様は、以下の初期プロセスに意見を出すことによって、ここに提案する生物多様性の知識のための連携協力体制の確立にぜひ貢献してください。

本件の詳細、また関連するディスカッションへの参加方法についてはallianceウェブサイトに掲載されています。後述の5つの分野に分けたディスカッションに参加するにはウェブサイトのDiscussionsタブをご覧ください。**英語以外の言語での参加も歓迎します。**

連携を拡大する

ワークショップ、そのレポート、この行動への呼びかけは、国際コミュニティに対して行われます。生物多様性情報を使える状態にし、改善し、統合し、利用したいと考えている個人および機関は、alliance ウェブサイトにおいてサインオンすることで支援の意思表明を行ってください。なおオプションとして今後の更新情報を購読することができます。

p.3: (*left*) Insect-egg mold (*Leocarpus fragilis*) by Tim Sage via **iNaturalist**, licensed under CC BY-NC 4.0; (*right*) Pacific sea nettle (*Chrysaora fuscescens*) by Robin Agarwal via **iNaturalist**, licensed under CC BY-NC 4.0.

お手本を評価する

この複雑で多様な利害関係者のコミュニティのニーズに取り組むためには、もっと作業が必要です。しかし、他の類似した連携協力、連合、協会（例えばオープンソースソフトウェアプロジェクトで用いられている能力主義の「Apache Way」など）が長期的アプローチの参考となるでしょう。また、メンバーシップ（個人、機関あるいはその両方）の基準などの重要な問いが残っています。

範囲を明確にして成果を出す

さらなる協力によって、確かに生物多様性情報学の分野に大きな利益と効率がもたらされますが、究極のゴールは、科学、政策、社会においてインパクトを与えることです。GBIC2の参加者は、多様な利害関係者に関わってもらい、進捗状況を調べる目的で定義付けの質問と達成可能な使用事例の一式を作成する必要があると提案しました。利害関係者には、研究グループ、分類学施設、CBD、IPBES、FAO、保全団体、その他のユーザーコミュニティが含まれます。これらは、協働による計画・開発・実施の上で優先順位を決めるためには十分正確で詳細である必要があります。

利害関係者を明確にする

連携協力活動のためにステークホルダーの状況を理解することは困難です。なぜなら、活動の数が多く、異なる活動間でのミッションの重複があり、作業計画の実施時期が異なり、責任の範囲も違うからです。この複雑な状況を理解しておかないとされなければ、努力が不適切に競合したり重複したりする重大なリスクがあります。GBIFは厳密かつ明確なスコープの範囲をもって、主要な機関どうしの関係、役割、責任を、特に世界、地域、国家スケールにおいて、最初の段階でネットワーク分析を行う調整をします。この努力によって、連携が創造あるいは維持すべき重要なサービスを特定し、よりよい連携や統合を生むための機会を示す一助となるでしょう。

概念を実証するプロジェクトを立てる

この連携協力の主な目標は、利害関係者が共通のニーズを中心に集合し持続可能なプロジェクトを立ち上げ、役立つツール、サービス、モデル、リソースを創出することです。それが結果的に相互に接続されたデジタル知識システムに役立つことが期待されています。このようなプロジェクトの優先順位づけ、創出、提供、維持のためには、公式なプロセスが必要です。短期的にみれば、オープンな連携協力の構想を支えている現在の活動を特定することは価値があり、プルーフ・オブ・コンセプト・プロジェクトとして採択できるでしょう。そのようなプロジェクトは、将来の管理体制モデルに組み込める教訓を提供でき、同時に連携協力の初期の例となります。初期のプルーフ・オブ・コンセプト・プロジェクトの候補としてふさわしい現在進行中の活動があれば提案してください。ソフトウェア開発やデータ管理だけではなく、能力開発や持続可能性の計画など他の分野についても対象となります。

Hobern D, Baptiste B, Copas K, Guralnick R, Hahn A, van Huis E, Kim E-S, McGeoch M, Naicker I, Navarro L, Noesgaard D, Price M, Rodrigues A, Schigel D, Sheffield CA & Wieczorek J (2019) Connecting data and expertise: a new alliance for biodiversity knowledge. Biodiversity Data Journal. doi:10.3897/BDJ.7.e33679
