# Supplementary material for: Connecting data and expertise: a new alliance for biodiversity knowledge
Source: Biodivers Data J. 2019 Mar 8;7:e33679. doi: 10.3897/BDJ.7.e33679 (PMC6420472; doi:10.3897/BDJ.7.e33679)
Supplement: Supplementary material 9 — Call: Een alliantie voor kennis over biodiversiteit [file bdj-07-e33679-s009.pdf]

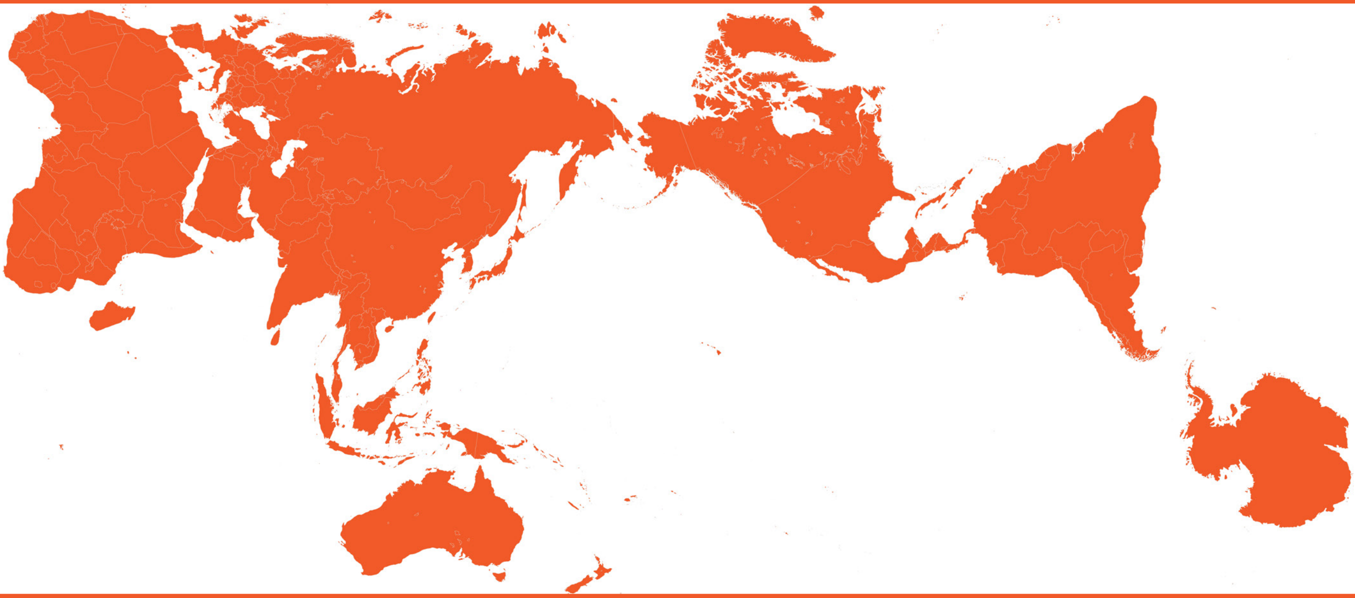

Call: **Een alliantie voor kennis over biodiversiteit**

VISIE

Er is de afgelopen twee decennia grote vooruitgang geboekt met het digitaliseren van historische kennis over onze biodiversiteit en met het vrij en open toegankelijk maken van deze biodiversiteitsgegevens. Tal van gecoördineerde inspanningen brengen internationale partnerschappen en netwerken, nationale, regionale en institutionele projecten, investeringen en talloze individuele bijdragers samen. Deze samenwerkingen strekken zich uit over diverse onderzoeksdomeinen (vnl. biologische en milieuwetenschappen), overheidsinstanties, NGO's, 'citizen science' initiatieven en zelfs commerciële ondernemingen.

De huidige inspanningen blijken echter ontoereikend om tegemoet te komen aan de wereldwijde behoefte aan nauwkeurige gegevens over soorten en over veranderende patronen en trends in de biodiversiteit. De grootste uitdagingen zijn onder meer:

- ongelijke regionale betrokkenheid bij 'biodiversity informatics'
- ongelijke voortgang bij het mobiliseren en delen van gegevens
- het ontbreken van 'stable persistent identifiers' voor data records
- redundante en incompatibele processen voor het opschonen en interpretatie van gegevens
- de afwezigheid van functionele mechanismen voor experten om gegevens te beheren en te verbeteren

De 'Global Biodiversity Information Facility' (GBIF) erkent de noodzaak tot meer afstemming van activiteiten tussen alle actoren en organiseerde daarop in juli 2018 de tweede 'Global Biodiversity Informatics Conference' (GBIC2) om een coördinerend mechanisme voor te stellen om gedeelde roadmaps voor Biodiversity-Informatics te ontwikkelen. De GBIC2-deelnemers bereikten consensus over een duidelijke behoefte aan een wereldwijde alliantie voor kennis over de biodiversiteit en laten zich inspireren door voorbeelden als de **Global Alliance for Genomics and Health** (GA4GH) en de open software communities onder de **Apache Software Foundation**. Deze initiatieven bieden stakeholders met gedecentraliseerde financiering en onafhankelijk bestuur modellen om middelen te combineren en duurzame oplossingen te ontwikkelen die inspelen op gemeenschappelijke behoeften.

Supplementary Material 9: Call: Een alliantie voor kennis over biodiversiteit
doi:10.3897/BDJ.7.e33679.suppl9

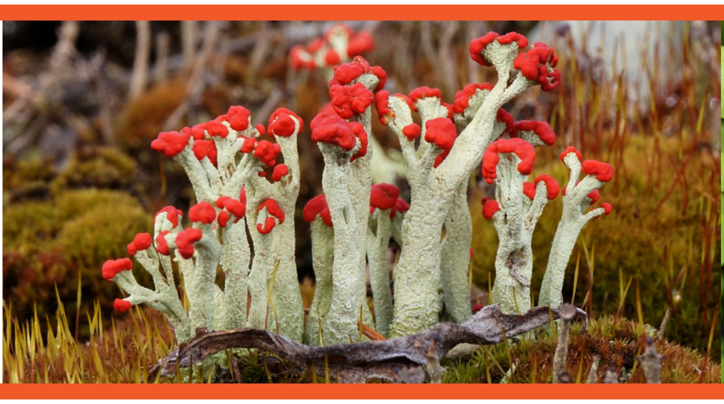
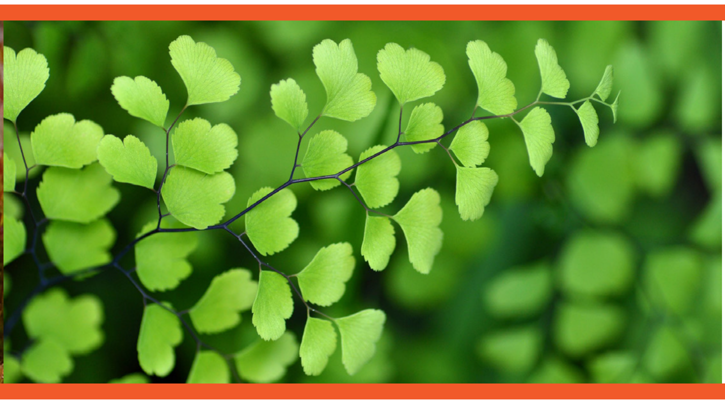

Meer samenwerking, verbeterd beheer van bestaande data en gecoördineerde ontwikkeling van nieuwe gegevensbronnen kan een geïntegreerde, onderling verbonden kennisbasis bieden voor alle aspecten van biodiversiteit - een die vrij en open wordt verspreid voor iedereen die behoefte heeft aan, of geïnteresseerd is in het gebruik ervan. Het realiseren van een dergelijk systeem zal ons in staat stellen de huidige wetenschappelijke kennis van biodiversiteit te verankeren in rationele en tijdige besluitvormingsprocessen. Dit ter ondersteuning van een duurzame toekomst.

AMBITIES

GBIC2 stelt de volgende veelzijdige visie voor om de ambities voor een alliantie voor biodiversiteitskennis te definiëren:

ONDERSTEUNING VAN WETENSCHAP EN 'EVIDENCE-BASED' PLANNING

1. Kennis en begrip over biodiversiteit leveren die gebouwd is op solide onderzoeksvragen en in staat stelt biodiversiteit correct kunnen meten en beoordelen in het belang van de maatschappij.
2. Een basis vormen voor fundamenteel onderzoek op het gebied van biodiversiteit en informatiewetenschap ter ondersteuning van het menselijk inzicht in het functioneren en de toestand van natuurlijke systemen.
3. Een platform bieden voor continue verbetering van het begrip van biodiversiteit door bestaande kennis te behouden, te vergroten en te verbeteren

ONDERSTEUNING VOOR 'OPEN DATA' EN 'OPEN SCIENCE'

4. Wegnemen van barrières rond vrij toegankelijke en 'open data', en adoptie van de FAIR-data principes (Wilkinson et al. 2016) voor biodiversiteitsgegevens
5. Beschrijven van alle gegevensbronnen met rijke metadata die het huidig gebruik en toekomstig hergebruik ondersteunen
6. Verzekeren dat alle datasets bewaard worden in stabiele en betrouwbare repositories
7. Faciliteren van collaboratief cureren, annoteren en verbeteren van alle data door iedere relevante experts en/of expertengemeenschappen
8. Zorgdragen dat bijdrage aan kennis en expertise geregistreerd en erkend wordt
9. Vastleggen en volgen van de herkomst en toewijzing van alle databronnen

ONDERSTEUNING VOOR STERK VERBONDEN BIODIVERSITEITSGEGEVENS

10. Mobiliseren van gestructureerde digitale representaties van historische gegevensbronnen, waaronder museumcollecties en literatuur
11. Zorg dragen dat alle nieuwe waarnemingen en metingen zo snel mogelijk na de opname toegankelijk zijn in gestructureerde digitale vorm
12. Het combineren, bevragen en analyseren van verschillende klassen van biodiversiteitsinformatie (distributie, eigenschappen, genen, enz.) mogelijk maken als zijnde een onderling verbonden geheel
13. Samenwerken met andere onderzoeksgemeenschappen en -infrastructuren om interoperabiliteit te bereiken met 'earth' observaties, sociale wetenschappen en andere databronnen

Image credits. p.1: AuthaGraph map of the world with political borders adapted from **Justin Kunimune**, licensed under CC BY-SA 4.0. ***p.2:*** *(left)* British soldier lichen (*Cladonia cristatella*), by Mike Lewinski via **iNaturalist**, licensed under CC BY 2.0; (*right*) Black maidenhair fern (*Adiantum capillus-veneris*) by Robby Deans via **iNaturalist**, licensed under CC BY-NC 4.0.

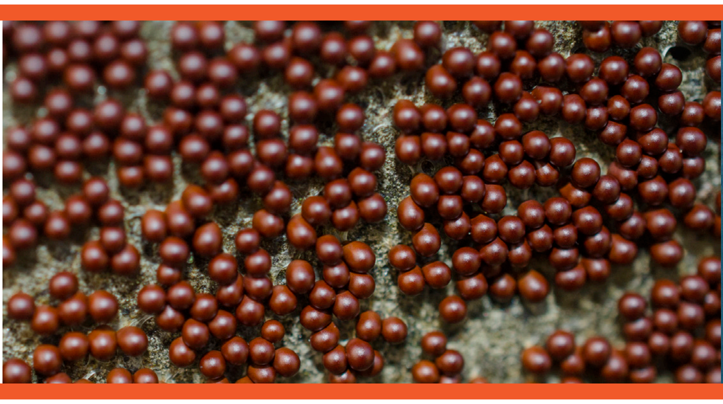

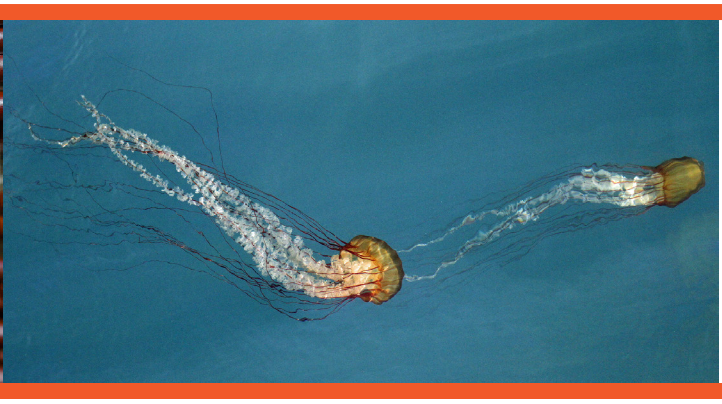

ONDERSTEUNING VOOR INTERNATIONALE SAMENWERKING

14. Onderkennen van capaciteitsbehoeften rond 'biodiversity informatics' in alle regio's en in alle sectoren
15. Financiering veilig stellen voor het onderhouden van diensten en componenten die de community herkent als kritieke elementen binnen een uitgebreide kennisinfrastructuur
16. Ontwikkeling van flexibele, collaboratieve benaderingen voor het ontwerpen, bouwen en onderhouden van alle componenten van deze kennisinfrastructuur
17. Stakeholders in alle landen en regio's in staat stellen te profiteren van de vooruitgang op het gebied van infrastructuur, tools, services, werkwijzen en capaciteit
18. Faciliteren van volledige participatie van en samenwerking met alle stakeholdergroepen in alle regio's in alle stadia mogelijk maken, van het genereren van gegevens tot de analyse en toepassing ervan
19. Data-repatriëring mogelijk maken die wetenschap en beleidsvorming in alle landen en regio's ondersteunt
20. Zorgen voor gemakkelijke effectieve toegang tot en gebruik van gegevens op elke schaal - mondiaal, regionaal, nationaal en lokaal
21. Erkennen van de rol van regionale, nationale en lokale investeringen als kritische en effectieve componenten van een mondiale oplossing
22. Wegnemen van belemmeringen voor gegevensuitwisseling of gebruik in taal of cultuur
23. Ondersteuning bieden voor de praktische uitvoering van internationale overeenkomsten met betrekking tot toegang en 'benefit sharing'

VOLGENDE STAPPEN

Alle stakeholders met interesse in het creëren, integreren, beheren en gebruiken van gegevens over de biodiversiteit, worden aangespoord om mee te helpen tot de oprichting van deze alliantie voor biodiversiteitskennis onder andere door input te leveren voor de volgende initiële processen.

Meer informatie en opportuniteiten om bij te dragen aan bijbehorende discussies worden op de website van de ***alliantie***, **biodiversityinformatics.org**. Ga naar het tabblad Discussions op de website om een bijdrage te leveren aan het overleg over elk van deze vijf topics **delk van deze vijf topics**. **Andere talen dan het Engels zijn zeker ook welkom**.

BETROKKENHEID VERGROTEN

De workshop, het rapport en de 'call' tot actie zijn voorbereid. Individuen en instellingen die geïnteresseerd zijn in het mobiliseren, verbeteren, integreren of gebruiken van biodiversiteitsinformatie worden aangemoedigd om hun interesse aan te geven door in te loggen en/of zich te abonneren op verdere updates van de website.

p.3: *(left)* Insect-egg mold (*Leocarpus fragilis*) by Tim Sage via **iNaturalist**, licensed under CC BY-NC 4.0; (*right*) Pacific sea nettle (*Chrysaora fuscescens*) by Robin Agarwal via **iNaturalist**, licensed under CC BY-NC 4.0.

MODELLEN EVALUEREN

Er is meer werk nodig om aan de behoeften van deze complexe en diverse community tegemoet te komen, maar modellen van andere soortgelijke allianties, coalities en consortia (zoals het verdiensten-gebaseerde 'Apache Way' die wordt gebruikt door open source softwareprojecten), kunnen richtlijnen voor de langere termijn sturen en informeren. Belangrijke vragen blijven bestaan rond de basis van lidmaatschap (individueel, institutioneel of beide).

VERDUIDELIJKEN VAN DE SCOPE EN DE DOELEN

Meer samenwerking zal ongetwijfeld interessante voordelen en efficiëntie opleveren voor het veld 'biodiversity informatics', maar het uiteindelijke doel is om impact te hebben voor wetenschap, beleid en samenleving. GBIC2-deelnemers stelden voor om verschillende stakeholders, waaronder onderzoeksgroepen, taxonomische initiatieven, het CBD, IPBES, FAO, organisaties rond natuurbehoud en andere communities, te betrekken bij het ontwikkelen van een reeks definiërende vragen en haalbare use-cases om vooruitgang te meten. Deze moeten voldoende nauwkeurig en gedetailleerd zijn om de prioriteiten te bepalen voor gezamenlijke planning, ontwikkeling en implementatie.

IDENTIFICATIE VAN STAKEHOLDERS

Het begrijpen van het landschap van belanghebbenden voor de alliantie is moeilijk, vanwege het grote aantal activiteiten, de vaak overlappende missies en de implementatie ervan op verschillende momenten en op verschillende schalen van verantwoordelijkheid.

Tenzij deze complexiteit wordt begrepen, is er een groot risico van onbedoeld conflict of dubbel werk. GBIF zal een eerste netwerkanalyse coördineren - één met strakke en duidelijk omlijnde limieten - waarbij de rollen, verantwoordelijkheden en relaties van grote organisaties worden geschetst, met name op wereldwijde, regionale en nationale schaal. Deze inspanning zal de alliantie helpen cruciale onderdelen te identificeren die moeten worden gecreëerd en/of moeten worden onderhouden, om zodoende kansen aan te geven voor een betere coördinatie.

GOEDKEUREN VAN PROOF-OF-CONCEPT PROJECTEN

Een belangrijk doel van de alliantie is om stakeholders in staat te stellen zich te verenigen rond gedeelde behoeften en om duurzame projecten te initiëren om zo hulpmiddelen, services, modellen en middelen te leveren die bijdragen aan het op punt zetten van een met elkaar verbonden digitaal kennissysteem. Formele processen zijn vereist om dergelijke projecten te prioriteren, te initiëren, aan te leveren en in stand te houden. Op korte termijn is het waardevol om huidige activiteiten te identificeren die de visie voor een open alliantie ondersteunen en die kunnen worden aanvaard als proof-of-concept projecten. Dergelijke projecten kunnen lessen bieden die kunnen worden opgenomen in het toekomstige management, terwijl ze tegelijkertijd voorbeelden kunnen geven van samenwerking op basis van allianties. We zoeken suggesties voor geschikte bestaande activiteiten als kandidaat voor vroege proof-of-concept projecten. Niet alleen voor softwareontwikkeling of gegevensbeheer, maar ook over andere gebieden zoals capaciteitsvergroting en planning rond duurzame ontwikkeling.

Hobern D, Baptiste B, Copas K, Guralnick R, Hahn A, van Huis E, Kim E-S, McGeoch M, Naicker I, Navarro L, Noesgaard D, Price M, Rodrigues A, Schigel D, Sheffield CA & Wieczorek J (2019) Connecting data and expertise: a new alliance for biodiversity knowledge. Biodiversity Data Journal. **doi:10.3897/BDJ.7.e33679**
