# Supplementary material for: Connecting data and expertise: a new alliance for biodiversity knowledge
Source: Biodivers Data J. 2019 Mar 8;7:e33679. doi: 10.3897/BDJ.7.e33679 (PMC6420472; doi:10.3897/BDJ.7.e33679)
Supplement: Supplementary material 10 — Apelo a uma aliança para o conhecimento da biodiversidade [file bdj-07-e33679-s010.pdf]

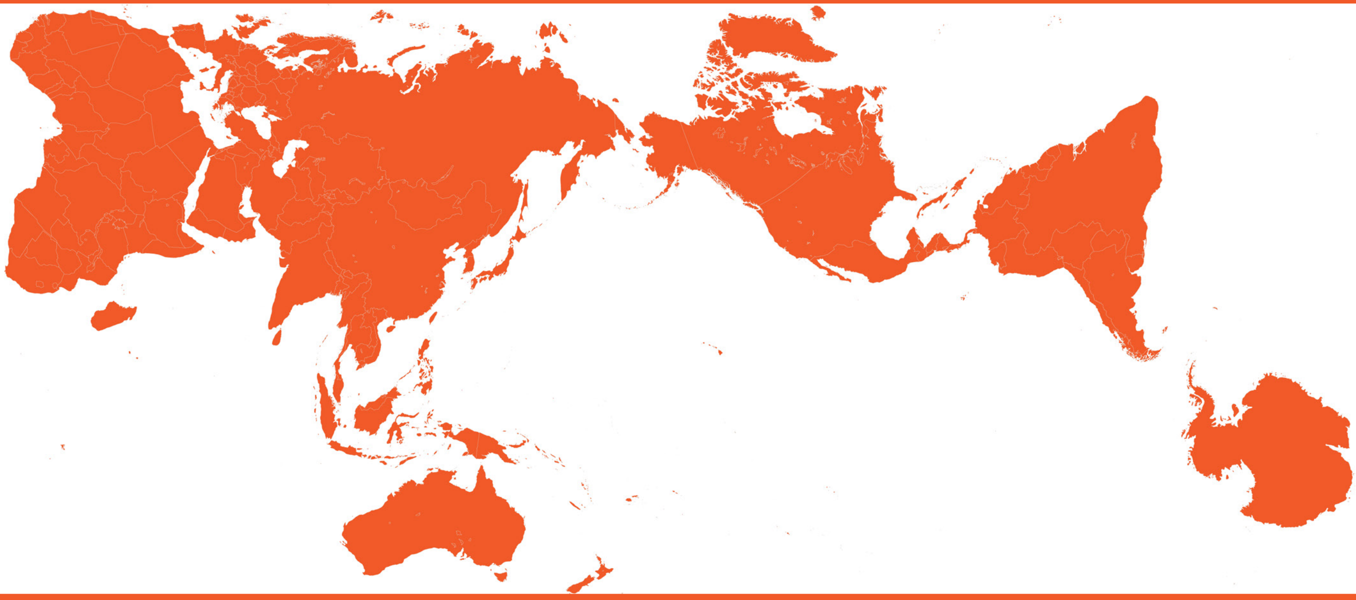

Apelo a
uma aliança para o conhecimento da biodiversidade

VISÃO

Houve grandes progressos nas últimas duas décadas na digitalização do conhecimento histórico da biodiversidade e na disponibilização livre e aberta de dados de biodiversidade. Os esforços de interligação reúnem parcerias e redes internacionais, projetos e investimentos nacionais, regionais, institucionais e inúmeros contribuidores individuais. Essas colaborações abrangem diversos domínios de pesquisa biológica e ambiental, agências governamentais e organizações não-governamentais, ciência cidadã e empresas comerciais.

No entanto, os esforços atuais permanecem ineficientes e inadequados para abordar a necessidade global de dados precisos sobre as espécies do mundo e sobre a mudança de padrões e tendências da biodiversidade. Os desafios significativos incluem::

- desequilíbrios no envolvimento regional na atividade Informática da biodiversidade
- progressos irregulares na mobilização e compartilhamento de dados
- a falta de identificadores persistentes e estáveis para registros de dados
- processos redundantes e incompatíveis para a limpeza e interpretação de dados
- a ausência de mecanismos funcionais para peritos curarem e melhorarem os dados

Reconhecendo a necessidade de maior alinhamento entre esforços em todas as escalas, o Global Biodiversity Information Facility (GBIF) convocou a segunda Conferência Mundial de Informática da Biodiversidade (GBIC2), em julho de 2018, para propor um mecanismo de coordenação para desenvolvimento de roteiros compartilhados para a Informática da Biodiversidade. Os participantes do GBIC2 chegaram a um consenso sobre a necessidade de uma aliança global para o conhecimento da biodiversidade, aprendendo a partir de exemplos como a **Aliança global para a genómica e saúde** (GA4GH) e as comunidades de software aberto sob a **Apache Software Foundation**. Essas iniciativas fornecem modelos para vários atores e partes interessadas, com financiamento descentralizado e governança independente, para combinar recursos e desenvolver soluções sustentáveis que abordem as necessidades comuns.

O aumento da cooperação, a melhoria da gestão dos dados existentes e o desenvolvimento coordenado de novas fontes de dados poderiam proporcionar uma base de conhecimento integrada

Supplementary Material 10: Apelo a uma aliança para o conhecimento da biodiversidade
doi:10.3897/BDJ.7.e33679.suppl10

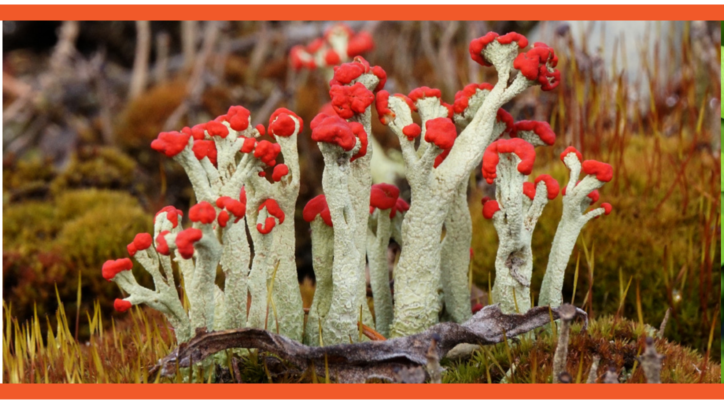
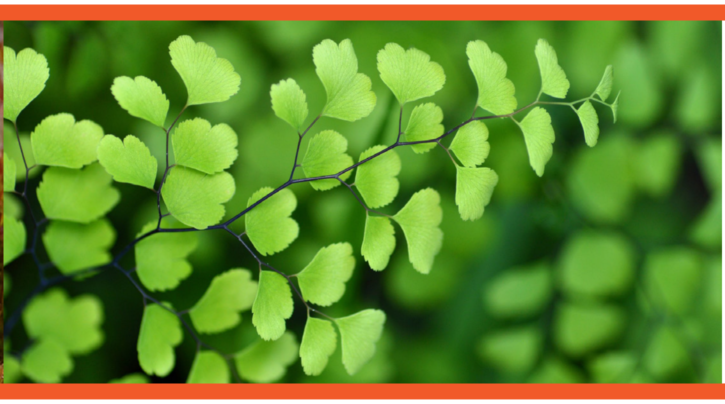

e interligada para todos os aspectos da biodiversidade — livre e abertamente disseminada para todos os que têm necessidade ou interesse em usar. O alcance deste sistema possibilitaria inserir o actual conhecimento científico dentro de processos de tomada de decisão racionais e oportunas, que suportem um futuro sustentável.

AMBIÇÕES

Os participantes da GBIC2 propuseram a seguinte visão multifacetada para definir as ambições de uma aliança para o conhecimento da biodiversidade:

APOIO AO PLANEAMENTO BASEADO EM CIÊNCIA E EM EVIDÊNCIAS

1. Fornecer conhecimento e compreensão sobre a biodiversidade para apoiar os requisitos críticos de pesquisa e que permitam que a biodiversidade seja corretamente medida e avaliada para objetivos sociais.
2. Servir como fundação para a pesquisa básica em biodiversidade e ciência da informação que atenda à compreensão humana sobre o funcionamento e estado dos sistemas naturais.
3. Proporcionar uma plataforma de crescimento contínuo para a compreensão da biodiversidade, preservando, construindo e melhorando o conhecimento existente.

SUPORTE PARA DADOS ABERTOS E CIÊNCIA ABERTA

4. Remover barreiras ao compartilhamento livre e aberto de dados e à adoção dos princípios de dados FAIR (**Wilkinson et al. 2016**), para dados de biodiversidade.
5. Descrever todos os recursos de dados com metadados ricos que suportem a reutilização no presente e no futuro.
6. Garantir que todos os recursos de dados sejam preservados em repositórios confiáveis, estáveis e persistentes.
7. Permitir a curadoria colaborativa, anotação e melhoria de todos os dados por quaisquer peritos e comunidades de peritos relevantes.
8. Permitir que todos os colaboradores de conhecimento ou especialização tenham as suas contribuições totalmente registradas, reconhecidas e creditadas.
9. Rastrear a proveniência e a atribuição de todas as fontes de informação

SUPORTE PARA DADOS DE BIODIVERSIDADE ALTAMENTE CONECTADOS

10. Mobilizar representações digitais estruturadas de fontes de dados históricos, incluindo coleções de museus e literatura.
11. Garantir que todas as novas observações e medições sejam acessíveis em representações digitais estruturadas, o mais rapidamente possível após a captura.
12. Permitir a combinação, consulta e análise de diferentes classes de informação sobre biodiversidade (distribuição, características, genes, etc.) como um todo interligado.
13. Trabalhar com outras comunidades e infraestruturas de pesquisa para alcançar interoperabilidade para as observações da Terra, dados de ciências sociais e outros recursos.

Image credits. p.1: AuthaGraph map of the world with political borders adapted from **Justin Kunimune**, licensed under CC BY-SA 4.0. ***p.2:*** *(left)* British soldier lichen (*Cladonia cristatella*), by Mike Lewinski via **iNaturalist**, licensed under CC BY 2.0; (*right*) Black maidenhair fern (*Adiantum capillus-veneris*) by Robby Deans via **iNaturalist**, licensed under CC BY-NC 4.0.

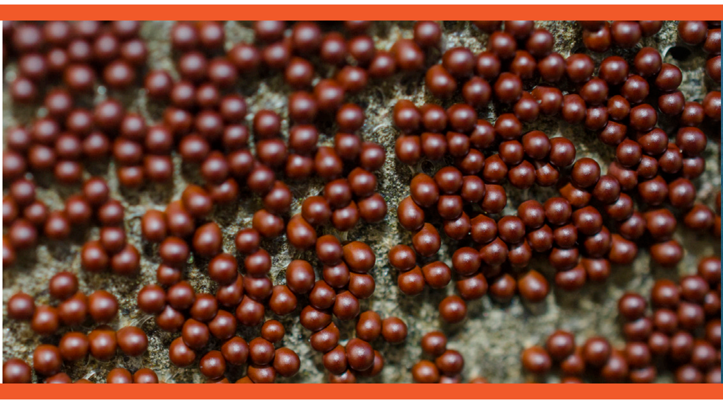
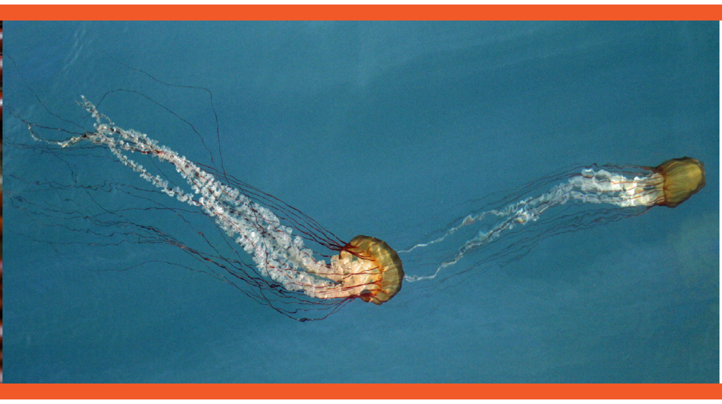

APOIO À COLABORAÇÃO INTERNACIONAL

14. Abordar as necessidades de formação em torno da Informática para a biodiversidade em todas as regiões e em todos os setores.
15. Garantir o financiamento para manter os serviços e componentes que a comunidade reconhece como elementos críticos dentro de uma infraestrutura de conhecimento distribuído.
16. Desenvolver abordagens flexíveis e colaborativas para desenvolver, construir e sustentar todos os componentes desta infraestrutura de conhecimento distribuído.
17. Permitir que as partes interessadas em todos os países e regiões adotem e se beneficiem dos avanços nas infraestruturas, ferramentas, serviços, práticas e formação.
18. Permitir a plena participação e colaboração entre e com todos os atores e partes interessadas em todas as regiões e em todas as fases, desde a geração de dados até a análise e aplicação.
19. Permitir o repatriamento de dados que apoiam a ciência e a elaboração de políticas em todos os países e regiões.
20. Assegurar o acesso eficaz e a utilização de dados em todas as escalas — globais, regionais, nacionais e locais.
21. Reconhecer e apoiar o papel dos investimentos locais, nacionais e regionais, como componentes efetivos e críticos dentro de uma solução global.
22. Ultrapassar as barreiras ao compartilhamento de dados ou ao uso decorrente de linguagem ou cultura.
23. Apoiar a aplicação prática de acordos internacionais com referência ao acesso e repartição de benefícios.

PRÓXIMOS PASSOS

Todos os atores e partes interessadas na produção, gestão, utilização e integração de dados sobre a biodiversidade mundial são incitados a contribuir para o estabelecimento desta aliança proposta para o conhecimento da biodiversidade, fornecendo subsídios para os seguintes processos iniciais.

Mais informações e oportunidades de contribuir para discussões associadas serão mantidas no site da ***aliança*** website, **biodiversityinformatics.org**. Visite a aba Discussões no portal para contribuir para discussões sobre cada uma dessas cinco áreas **discussões sobre cada uma dessas cinco áreas**. **Contribuições em idiomas diferentes do inglês são bem-vindas.**.

EXPANDIR O ENVOLVIMENTO

O workshop, o seu relatório, e este apelo à ação foram preparados para a comunidade global. Os indivíduos e instituições com interesse em mobilizar, melhorar, integrar ou utilizar informações sobre biodiversidade são encorajados a indicar o seu apoio, assinando em apoio e/ou inscrevendo-se para novas atualizações no portal da aliança,, **biodiversityinformatics.org**.

p.3: *(left)* Insect-egg mold (*Leocarpus fragilis*) by Tim Sage via **iNaturalist**, licensed under CC BY-NC 4.0; (*right*) Pacific sea nettle (*Chrysaora fuscescens*) by Robin Agarwal via **iNaturalist**, licensed under CC BY-NC 4.0.

AVALIAR MODELOS

Mais trabalho é necessário para atender às necessidades desta comunidade complexa e diversificada de atores e partes interessadas, mas modelos de outras alianças semelhantes, coalizões e consórcios (como o "Apache Way" baseado em mérito, em uso por projetos de software de código aberto), podem orientar e informar abordagens de longo prazo. Questões importantes também permanecem em torno da base da adesão (indivíduos, institucionais ou ambos).

CLARIFICAR O ESCOPO E OS RESULTADOS-ALVO

Uma maior cooperação trará, sem dúvida, benefícios e eficiências significativas no campo da informática para a biodiversidade, mas o objetivo final é proporcionar impacto para a ciência, a política e a sociedade. Os participantes do GBIC2 se propuseram a envolver diversas partes interessadas—incluindo grupos de pesquisa, recursos taxonômicos, CBD, IPBES, FAO, organismos de conservação e outras comunidades de usuários—para desenvolver um conjunto de questões definidoras e casos de uso realizáveis contra os quais medir o progresso. Estes devem ser suficientemente precisos e detalhados para orientar as prioridades para o planeamento, desenvolvimento e implementação colaborativos.

IDENTIFICAR AS PARTES INTERESSADAS

Entender o panorama de atores e partes interessadas para o trabalho da aliança é difícil, devido ao número de atividades, às suas missões frequentemente sobrepostas, e à sua implementação através de programas de trabalho em diferentes prazos e em diferentes escalas de responsabilidade. A menos que essa complexidade seja compreendida, há um grande risco de conflito inadvertido ou duplicação de esforços. O GBIF coordenará uma análise de rede inicial — uma com limites firmemente e claramente delimitados — buscando delinear os papéis, as responsabilidades e as relações das grandes organizações, especialmente em escalas globais, regionais e nacionais. Este esforço ajudará a aliança a identificar serviços críticos que precisam ser criados ou sustentados, e indicar oportunidades para um melhor alinhamento ou unificação.

ADOTAR PROJETOS DE PROVA DE CONCEITO

Um dos principais objetivos da aliança é capacitar os atores e partes interessadas a convergir em torno de necessidades compartilhadas e incubar projetos sustentáveis para fornecer ferramentas, serviços, modelos e recursos que contribuam para a entrega de um sistema de conhecimento digital interconectado. Serão necessários processos formais para priorizar, incubar, entregar e sustentar tais projetos. A curto prazo, há valor na identificação de atividades atuais que apoiam a visão de uma aliança aberta e que podem ser adotadas como projetos de prova de conceito. Tais projetos podem oferecer lições que poderão ser incorporadas a modelos futuros de governança e, ao mesmo tempo, dar exemplos iniciais de cooperação baseada em alianças. Buscamos sugestões para atividades existentes adequadas como candidatas para projetos de prova de conceito antecipado, não apenas em desenvolvimento de software ou gerenciamento de dados, mas também em outras áreas, como aprimoramento de capacidade e planeamento de sustentabilidade.

Hobern D, Baptiste B, Copas K, Guralnick R, Hahn A, van Huis E, Kim E-S, McGeoch M, Naicker I, Navarro L, Noesgaard D, Price M, Rodrigues A, Schigel D, Sheffield CA & Wieczorek J (2019) Connecting data and expertise: a new alliance for biodiversity knowledge. Biodiversity Data Journal. **doi:10.3897/BDJ.7.e33679**
