# Supplementary material for: Connecting data and expertise: a new alliance for biodiversity knowledge
Source: Biodivers Data J. 2019 Mar 8;7:e33679. doi: 10.3897/BDJ.7.e33679 (PMC6420472; doi:10.3897/BDJ.7.e33679)
Supplement: Supplementary material 11 — Призыв к созданию альянса знаний по биоразнообразию [file bdj-07-e33679-s011.pdf]

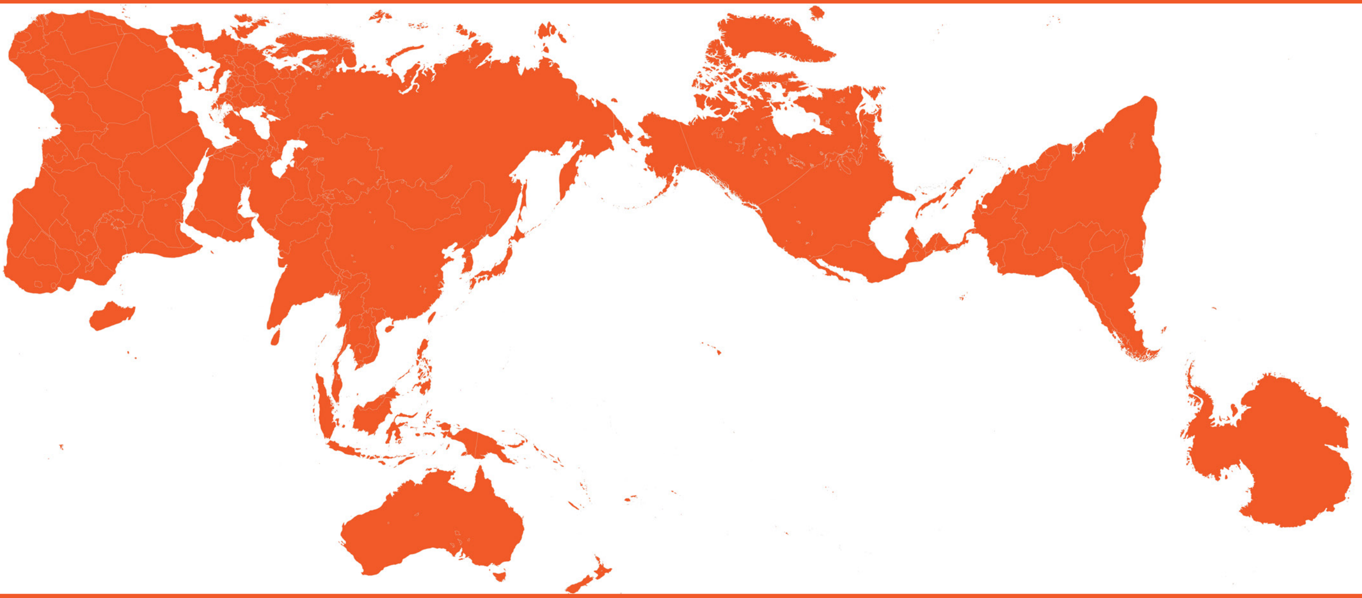

Призыв к созданию альянса знаний по биоразнообразию

КОНЦЕПЦИЯ

В последние два десятилетия был достигнут значительный прогресс в оцифровке и обеспечении свободного и открытого доступа к историческим данным о биоразнообразии. Взаимосвязанные усилия объединяют международные партнерства и сети, национальные, региональные и локальные проекты и инвестиции, и большое количество участников. Это сотрудничество охватывает широкое разнообразие дисциплин биологии и экологии, правительственные и неправительственные организации, инициативы гражданской науки и коммерческие проекты.

Тем не менее, текущие усилия все еще недостаточны для удовлетворения потребности в точных данных о глобальном биоразнообразии и трендах его изменения. Основные препятствия к этому:

- дисбаланс в региональных инициативах информатики биоразнообразия
- неравномерный прогресс в мобилизации и обмене данными
- отсутствие стабильных постоянных идентификаторов данных о находках видов
- избыточность и несогласованность процессов верификации и интерпретации данных
- отсутствие функциональных механизмов курирования данных экспертами

Признавая необходимость обеспечения большей согласованности усилий на всех уровнях, Глобальная информационная система по биоразнообразию (GBIF) организовала вторую Международную конференцию по информатике биоразнообразия (GBIC2) в июле 2018 года. На этой конференции была инициирована программа разработки общедоступных дорожных карт в области информатики биоразнообразия. Участники GBIC2 пришли к согласию в отношении необходимости создания глобального альянса знаний по биоразнообразию, опираясь на такие примеры, как **Международный альянс по геномике и здравоохранению** (GA4GH) и сообщество программного обеспечения с открытым исходным кодом **Apache Software Foundation**. Эти инициативы предоставляют модели объединения ресурсов и разработки устойчивых общих решений с децентрализованным финансированием и независимым управлением для партнеров и заинтересованных сторон.

Supplementary Material 11: Призыв к созданию альянса знаний по биоразнообразию
doi:10.3897/BDJ.7.e33679.suppl11

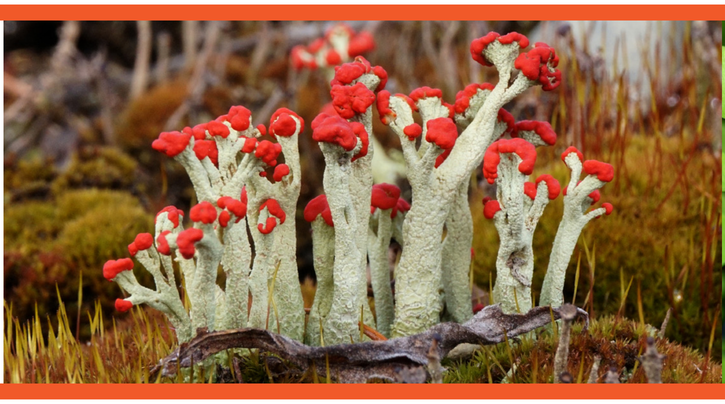 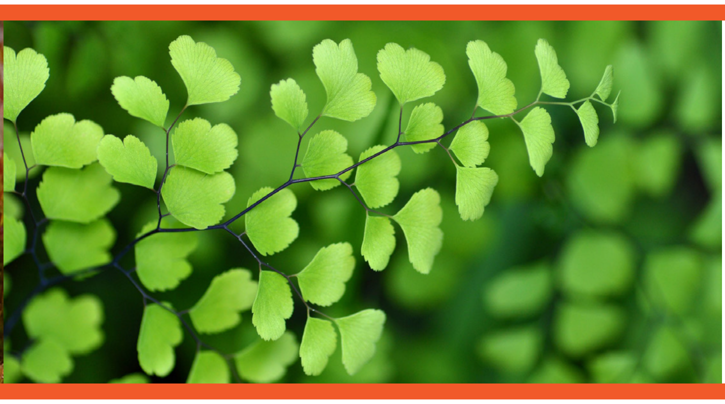

Расширение сотрудничества, улучшение менеджмента существующих данных и скоординированное развитие по публикации новых источников данных смогут предоставить интегрированную, взаимосвязанную базу знаний по всем аспектам биоразнообразия, свободно и открыто распространяемую среди всех, кто нуждается и заинтересован в их использовании. Такая система позволит внедрять современные научные знания о биоразнообразии в программы принятия решений для поддержания модели устойчивого развития.

ЦЕЛИ

Участники GBIC2 предложили комплексную концепцию и комплексные функции альянса знаний по биоразнообразию, включающую следующие пункты:

ПОДДЕРЖКА НАУКИ И ОБОСНОВАННОГО ПЛАНИРОВАНИЯ

1. Предоставлять знания о биоразнообразии в формах, удовлетворяющих исследовательским задачам и позволяющих корректно измерять и оценивать биоразнообразие для нужд общества.
2. Служить основой для фундаментальных исследований в области биоразнообразия и информатики для понимания основ функционирования и состояния природных экосистем
3. Обеспечивать платформу для постоянного роста осмысления биоразнообразия путем хранения, наращивания и улучшения существующих знаний

ПОДДЕРЖКА ОТКРЫТЫХ ДАННЫХ И ОТКРЫТОЙ НАУКИ

4. Устранять барьеры перед свободной и открытой публикацией и использованем данных, а также к применению принципов FAIR к данным по биоразнообразию (Wilkinson et al. 2016)
5. Обеспечить все источники данных подробными метаданными для поддержки их повторного использования в настоящем и будущем
6. Обеспечить хранение всех ресурсов данных в стабильных, постоянных и надежных репозиториях
7. Обеспечить совместное курирование, оценку и улучшение качества данных экспертами и их сообществами
8. Обеспечить авторам и экспертам полноценное признание их вклада через цитирование и благодарности
9. Отслеживать происхождение и авторство всех источников информации

ПОДДЕРЖКА ВЗАИМОСВЯЗАННЫХ ДАННЫХ О БИОРАЗНООБРАЗИИ

10. Мобилизоватьструктурированныецифровыеданныеисторическихисточников,включая музейные коллекции и научную литературу
11. Обеспечить доступность данных новых наблюдений или измерений в структурированной цифровой форме с минимальной задержкой после их появления
12. Обеспечить возможность объединения, запросов и анализа различных классов информации о биоразнообразии (распространение, признаки, гены и пр.) как частей взаимосвязанной системы

Image credits. p.1: AuthaGraph map of the world with political borders adapted from **Justin Kunimune**, licensed under CC BY-SA 4.0. ***p.2:*** *(left)* British soldier lichen (*Cladonia cristatella*), by Mike Lewinski via **iNaturalist**, licensed under CC BY 2.0; (*right*) Black maidenhair fern (*Adiantum capillus-veneris*) by Robby Deans via **iNaturalist**, licensed under CC BY-NC 4.0.

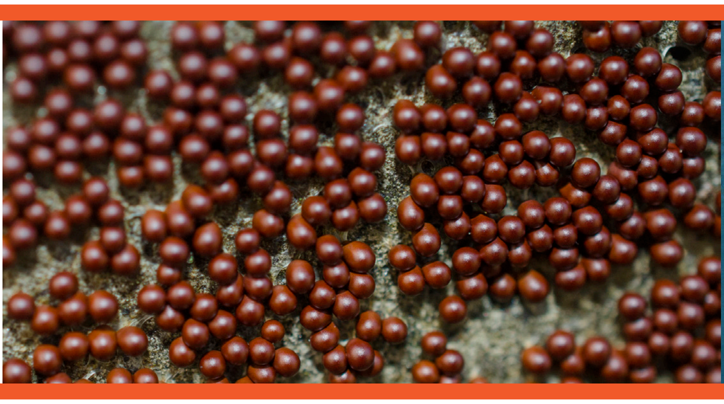 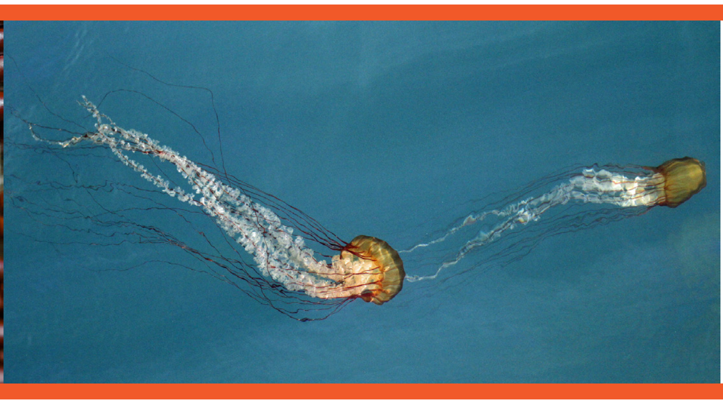

13. Работать с другими исследовательскими инфраструктурами и сообществами для обеспечения совместимости с другими естественно-научными наблюдениями, данными общественных наук, и других ресурсов

ПОДДЕРЖКА МЕЖДУНАРОДНОГО СОТРУДНИЧЕСТВА

14. Поддерживать наращивание потенциала и развитие ресурсов информатики биоразнообразия во всех регионах и секторах
15. Обеспечивать финансирование и поддержку отдельных критически важных компонентов и сервисов внутри распределенной инфраструктуры
16. Развивать гибкие совместные подходы к проектированию, созданию и поддержке всех компонентов распределенной инфраструктуры знаний
17. Дать возможность заинтересованным сторонам в каждой стране и регионе выгодно использовать инфраструктуру, а также инструменты, сервисы, подходы и ресурсы
18. Обеспечить полноценное участие и сотрудничество среди всех регионов и групп заинтересованных сторон на всех этапах, от сбора данных до их анализа и применения
19. Обеспечить репатриацию данных для поддержки науки и принятия политических решений во всех странах и регионах
20. Обеспечить активный доступ к данным и их использование на всех уровнях – глобальном, региональном, национальном и локальном
21. Признавать и поддерживать роль региональных, национальных и локальных инвестиций как критически важных и эффективных компонентов глобальной системы
22. Преодолевать барьеры на пути обмена данными, возникающие из-за языковых и культурных различий
23. Поддерживать практическое использование международных соглашений в контексте доступа и совместного использования выгод

ДАЛЬНЕЙШИЕ ШАГИ

Все стороны, заинтересованные в создании, управлении, использовании и интеграции данных о мировом биоразнообразии призываются внести свой вклад в организацию предложенного альянса знаний по биоразнообразию, посредством участия в следующих инициативах.

Дополнительная информация и возможности принять участие в обсуждениях размещены и поддерживаются на сайте ***альянса***, **biodiversityinformatics.org**. В разделе *Discussions* предусмотрена возможность участия в **дискуссиях по каждой из пяти тем**. **Приветствуется участие на других языках, кроме английского**.

РАСШИРЕНИЕ ВОВЛЕЧЕННОСТИ СТОРОН

Семинар, резолюция и этот призыв к действию были подготовлены и представлены глобальному сообществу. Организации и отдельные лица, заинтересованные в мобилизации, улучшении, интеграции и использовании данных о биологическом разнообразии приглашаются выразить свою поддержку через подписку на обновления сайта ***альянса***, **biodiversityinformatics.org**.

p.3: (*left*) Insect-egg mold (*Leocarpus fragilis*) by Tim Sage via **iNaturalist**, licensed under CC BY-NC 4.0; (*right*) Pacific sea nettle (*Chrysaora fuscescens*) by Robin Agarwal via **iNaturalist**, licensed under CC BY-NC 4.0.

ОЦЕНКА МОДЕЛЕЙ

Для того, чтобы оценить потребности комплексного и разнообразного сообщества заинтересованных сторон, требуется большая работа. Модели других подобных союзов, коалиций и консорциумов, в том числе таких, как сообщество программного обеспечения с открытым, совместно разрабатываемым кодом 'Apache Way', могут служить ориентиром и направлять долгосрочное развитие. Важными остаются вопросы членства (отдельные лица, организации или смешанное).

УТОЧНЕНИЕ СФЕРЫ ДЕЯТЕЛЬНОСТИ И ЗАДАЧ

Более тесное сотрудничество несомненно позволит привнести значительные выгоды и применять эффективные решения в информатике биоразнообразия, но конечной целью является вклад в науку, политику и общество. Участники GBIC2 предложили привлечь заинтересованные стороны, включая исследовательские группы, таксономические инфраструктуры, Конвенцию по Биологическому Разнообразию (CBD), Межправительственную научно-политическую платформу по биоразнообразию и экосистемным услугам (IPBES), Продовольственную и сельскохозяйственную организацию Объединенных Наций (FAO), природоохранные организации и другие сообщества к разработке ключевых вопросов и вариантов использования, которые позволят отслеживать прогресс. Эти вопросы и варианты должны быть достаточно конкретны и детальны для выработки приоритетных направлений для совместного планирования, развития и реализации.

ВЫЯВЛЕНИЕ ЗАИНТЕРЕСОВАННЫХ СТОРОН

Понимание спектра заинтересованных сторон для работы в рамках альянса представляется затруднительным из-за большого числа видов деятельности, пересекающихся целей и различий во временных масштабах и масштабах ответственности. До решения этих затруднений будет существовать риск непреднамеренного конфликта или дублирования усилий. GBIF проведет первичный анализ сети с четкими и определенно поставленными задачами, определяя роли, сферы ответственности и связи между основными организациями, особенно на глобальном, региональном и национальном уровнях. Эти действия помогут определить сервисную сферу задач альянса, роли, которые он будет создавать и поддерживать, а также возможность оптимального объединения и регулирования.

ЗАПУСК ПИЛОТНЫХ ПРОЕКТОВ

Ключевая цель альянса — это объединение усилий заинтересованных сторон для удовлетворения общих нужд, и создание благоприятных условий для развития проектов по обеспечению инструментами, сервисами, моделями и ресурсами, способствующих развитию информационной сети знаний. Для того, чтобы расставить приоритеты, заложить и поддерживать развитие подобных проектов, будут необходимы формальные процедуры. В краткосрочной перспективе, имеет смысл отобрать ряд существующих проектов для заложения основ альянса и демонстрации его ценности. Такие проекты могут служить примерами для развития моделей управления, и в то же время будут первыми направлениями сотрудничества в рамках альянса. Мы будем рады получить предложения о включении в число пилотных проекты в области развития программного обеспечения и управления данными, а также проекты, направленные на наращивание потенциала и планирование устойчивого развития.

Hobern D, Baptiste B, Copas K, Guralnick R, Hahn A, van Huis E, Kim E-S, McGeoch M, Naicker I, Navarro L, Noesgaard D, Price M, Rodrigues A, Schigel D, Sheffield CA & Wieczorek J (2019) Connecting data and expertise: a new alliance for biodiversity knowledge. Biodiversity Data Journal. **doi:10.3897/BDJ.7.e33679**
