# Supplementary material for: Connecting data and expertise: a new alliance for biodiversity knowledge
Source: Biodivers Data J. 2019 Mar 8;7:e33679. doi: 10.3897/BDJ.7.e33679 (PMC6420472; doi:10.3897/BDJ.7.e33679)
Supplement: Supplementary material 12 — 对建立生物多样性知识联盟的呼吁 [file bdj-07-e33679-s012.pdf]

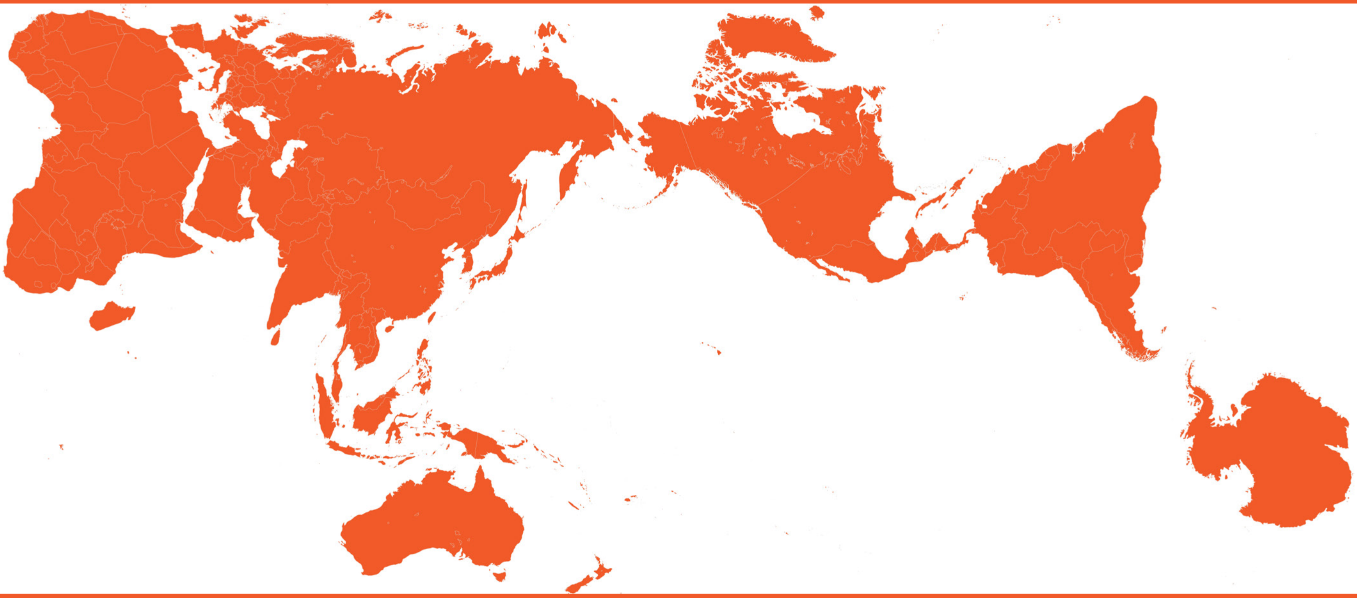

对建立生物多样性知识联盟的呼吁

愿景

在过去二十年里，围绕数字化生物多样性的历史知识，以及在使生物多样性数据自由和公开获取方面取得了重大进展。 交错的工作努力将国际伙伴关系和网络、国家、区域和机构项目和投资，以及无数个人贡献者聚集在一起。 这些合作涉及多个生物和环境研究领域、政府机构和非政府组织、公民科学和商业企业。

但是，目前的努力仍然效率低下，不足以满足全球对世界物种准确数据以及生物多样性格局和趋势变化的需求。重大的挑战包括：

- 区域参与生物多样性信息学活动的不平衡
- 数据调动和共享的进程不均衡
- 缺少数据记录的稳定持久的身份标识
- 用于清理和解释数据的冗余和不兼容的过程
- 缺乏有资质的专家管理、改进数据的功能机制

认识到需要在各种层面的努力之间实现更大的一致性，全球生物多样性信息网络（GBIF）于2018年7月召开了第二届全球生物多样性信息大会（GBIC2），以提出一个协调机制，从而制定生物多样性信息学的共同路线图。GBIC2与会者就建立生物多样性知识全球联盟的必要性达成了共识，从全球基因组学与健康联盟（GA4GH）和Apache软件基金会下的开放软件社区等实例中学习。这些举措为多个利益攸关者提供了分散资金和独立治理的模式，以整合资源并开发满足共同需求的可持续解决方案。

加强合作，改进对现有数据的管理以及协调开发新的数据来源，可以为生物多样性的所有方面提供一个综合的、相互关联的知识库——可以自由和公开地传播给有需要或有兴趣使用它的每个人。实现这样一个系统将会使我们能够将当前对生物多样性的科学理解融入到支持可持续未来的合理和及时的决策过程中。

Supplementary Material 12: 对建立生物多样性知识联盟的呼吁
doi:10.3897/BDJ.7.e33679.suppl12

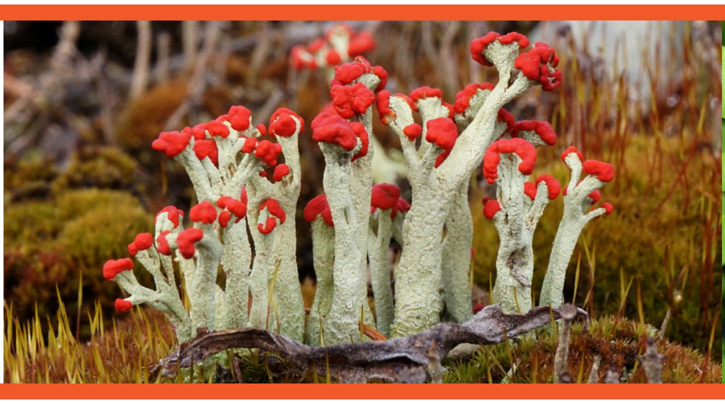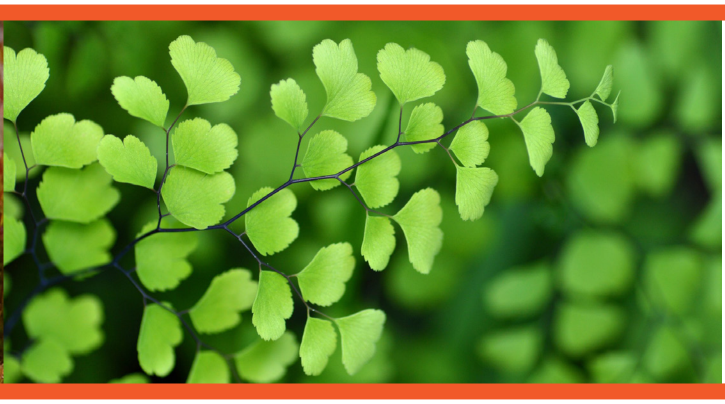

共同的志向

GBIC2的与会者提出了以下多方面的愿景，以确定生物多样性知识联盟的志向：

支持科学和循证规划

1. 为支持关键研究要求的形式而提供生物多样性的知识和理解，并使生物多样性得到正确的测量和评估，以实现社会目标
2. 作为对生物多样性和信息科学基础性研究的基础，使人类了解自然系统的功能和状态
3. 通过保护、建立和改进现有的知识，为不断增长的对生物多样性的理解提供平台

支持开放的数据和开放的科学

4. 消除自由和公开共享数据的障碍，并采用生物多样性数据的FAIR数据原则（Wilkinson等人（2016年））
5. 对所有数据资源加以丰富的元数据描述以支持当前和将来的重复使用
6. 确保所有数据资源存储在稳定、持久且受信任的存储库中
7. 实现任何相关专家和专家团体对所有数据的协作管理、注释和改进
8. 使所有知识或专长的贡献者的贡献都能够被充分记录、感谢和认可
9. 跟踪所有信息来源的来源和归属

支持高度关联的生物多样性数据

10. 调动有结构的、数字化的历史数据资源的呈现，包括标本馆藏和文献
11. 确保在获取到之后尽快在结构化数字表现中能够访问所有新的观测和测量
12. 使对不同类别的生物多样性信息（分布、性状、基因等）的组合、查询和分析成为一个相互关联 整体
13. 与其他研究团体和机构合作，实现与地球观测、社会科学数据和其他资源的互操作性

Image credits. p.1: AuthaGraph map of the world with political borders adapted from **Justin Kunimune**, licensed under CC BY-SA 4.0. ***p.2:*** *(left)* British soldier lichen (*Cladonia cristatella*), by Mike Lewinski via **iNaturalist**, licensed under CC BY 2.0; (*right*) Black maidenhair fern (*Adiantum capillus-veneris*) by Robby Deans via **iNaturalist**, licensed under CC BY-NC 4.0.

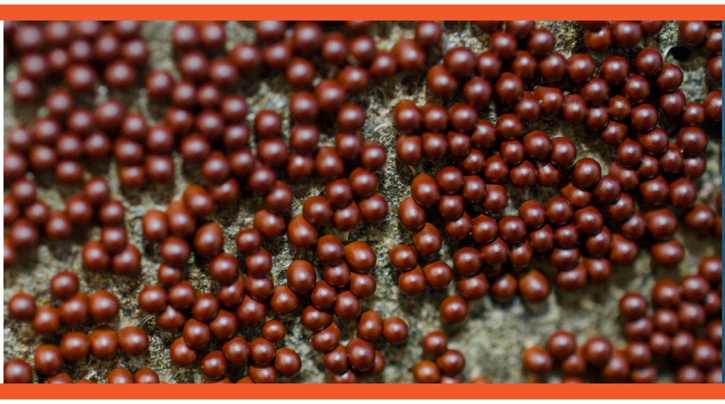
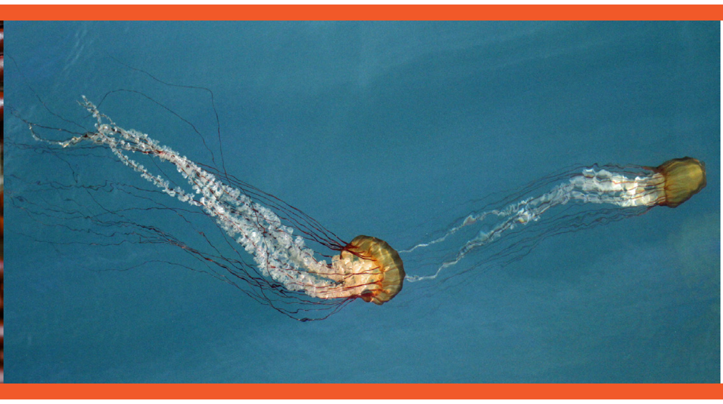

支持国际合作

14. 满足所有区域和所有领域的生物多样性信息学的能力需求
15. 获得资金以维持服务和被领域内认为是分布式知识基础设施中的关键要素的组成部分
16. 制定灵活的协作方法，以设计、建立和维持这种分布式知识基础设施的所有组成部分
17. 使每个国家和区域的利益攸关方能够采用基础设施，并从工具、服务、实践和能力的进步中受益
18. 从数据生成到分析和应用，使所有地区的所有利益攸关方群体充分参与和协作
19. 实现支持所有国家和地区的科学和决策的数据汇回
20. 确保数据在不同尺度上——全球的、地区的、国家和地方的有效获取和使用
21. 承认并支持地区、国家和地方的投资在全球解决方案中作为关键和有效组成部分的作用
22. 克服因语言或文化而产生的数据共享或使用的障碍
23. 支持在获取和惠益分享方面切实执行国际协定

下一步

敦促所有对世界生物多样性数据的生成、管理、使用和整合感兴趣的利益攸关方通过为以下初始过程提供投入，为建立这一拟议的生物多样性知识联盟做出贡献。

加入以下五个领域中的相关讨论。 **欢迎使用英语以外的语言。**

扩大参与度

研讨会及其报告和这一行动的呼吁都是为全球社会准备的。鼓励有兴趣调动、改进、整合或利用生物多样性信息的个人和机构通过在联盟网站上签署支持和/或订阅获取进一步的更新，来表明他们的支持。

p.3: *(left)* Insect-egg mold (*Leocarpus fragilis*) by Tim Sage via **iNaturalist**, licensed under CC BY-NC 4.0; (*right*) Pacific sea nettle (*Chrysaora fuscescens*) by Robin Agarwal via **iNaturalist**, licensed under CC BY-NC 4.0.

评估模型

需要做更多的工作来满足这个复杂多样的利益攸关方群体的需求，但其他类似联盟、联合会和联合体的模型（例如开源软件项目使用的择优的"Apache 方式"），可以指导和通报较长期的方法。关于成员资格（个人、机构或两者）的基础也存在重要的问题。

明确范围和目标结果

毫无疑问，加强合作将在生物多样性信息学领域带来显著的效益和效率，但最终目标是为科学、政策和社会带来影响。GBIC2与会者建议使不同的利益攸关者——包括研究组、生物分类设施、《生物多样性公约》（CBD）、"生物多样性和生态系统服务政府间科学政策平台"（IPBES）、联合国粮食及农业组织（FAO）、保护机构，以及其他用户群体——参与制定一套定义问题和可实现的用例，以衡量进展情况。这些应足够精确和详细，以指导用以协作规划、开发和实施的优先事项。

识别利益攸关者

由于活动的数量、经常重叠的任务以及通过不同时间表和不同责任范围的工作方案的实施，导致难以了解利益攸关方对联盟工作的影响。除非理解这种复杂性，否则存在无意冲突或重复工作的巨大风险。 GBIF将协调一个初步的网络分析 ——一个具有严格和明确范围限制的分析——寻求概述主要机构的作用、责任和关系，特别是在全球、区域和国家的范围内。这项工作将有助于联盟确定需要创建或维持的关键服务，并指明更好的协调或统一的机会。

采用概念验证项目

该联盟的一个关键目标是使利益攸关者能够围绕共同需求进行融合，并孵化可持续项目，以提供有助于传递互联数字知识系统的工具、服务、模型和资源。将需要正式的流程来确定此类项目的优先次序、孵化、交付和维持。在短期内，确定支持开放联盟愿景的当前活动是有价值的，可以作为概念验证项目采用。这些项目能够提供可以纳入未来治理模式的经验教训，同时提供基于联盟的合作的早期实例。我们寻求适当的现有活动的建议，作为早期概念验证项目的候选者，不仅在软件开发或数据管理方面，并且在其他领域，如能力提升和可持续性规划。

Hobern D, Baptiste B, Copas K, Guralnick R, Hahn A, van Huis E, Kim E-S, McGeoch M, Naicker I, Navarro L, Noesgaard D, Price M, Rodrigues A, Schigel D, Sheffield CA & Wieczorek J (2019) Connecting data and expertise: a new alliance for biodiversity knowledge. Biodiversity Data Journal. doi:10.3897/BDJ.7.e33679
