# Supplementary material for: Connecting data and expertise: a new alliance for biodiversity knowledge
Source: Biodivers Data J. 2019 Mar 8;7:e33679. doi: 10.3897/BDJ.7.e33679 (PMC6420472; doi:10.3897/BDJ.7.e33679)
Supplement: Supplementary material 13 — 呼籲成立生物多樣性知識聯盟 [file bdj-07-e33679-s013.pdf]

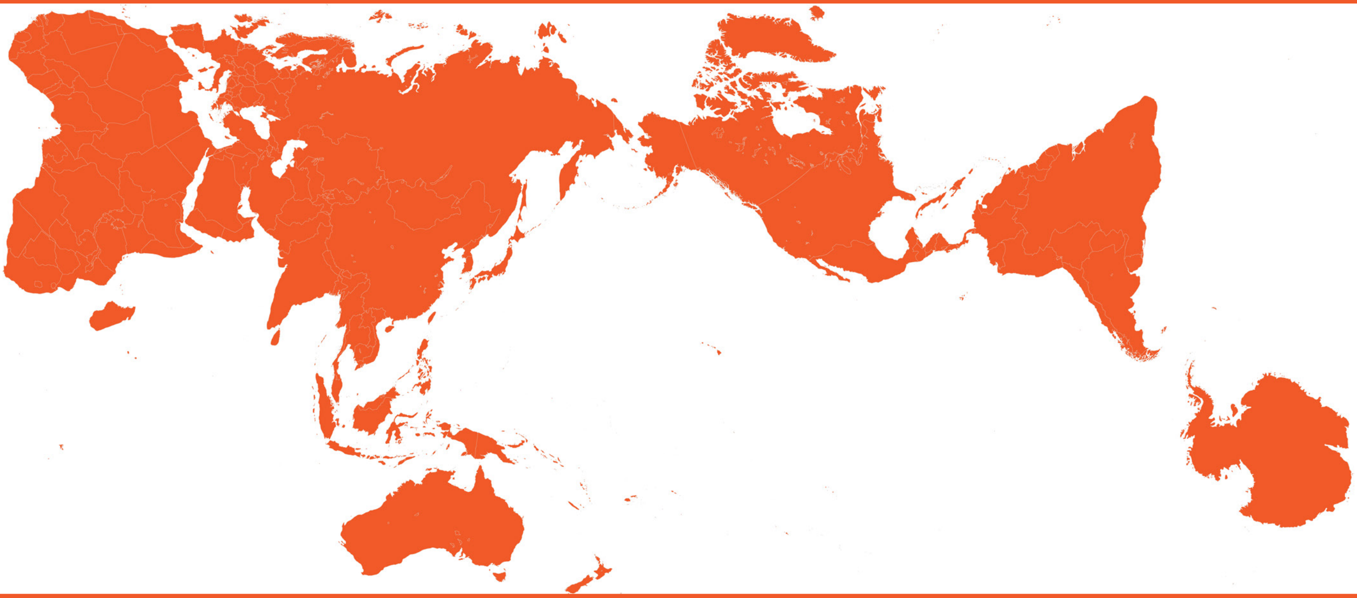

呼籲成立生物多樣性知識聯盟

願景

生物多樣性歷史知識的數位化與生物多樣性資料的開放存取，在過去二十年間有重大的進展。國際夥伴關係與網絡、國家、區域和機構計畫和投資，以及無數的個人貢獻者，因為相互連結的努力付出而集結在一起；這些合作跨越多個生物和環境研究領域、政府機構和非政府組織、公民科學和商業企業。

然而，對於解決全球在世界物種、生物多樣性的變動模式及趨勢之精確資料的需求，目前的努力仍然不夠恰當，也不夠有效。重大的挑戰包括：

- 區域間生物多樣性資訊活動的參與不平衡；
- 資料流通及共享的進展不一致；
- 資料記錄缺乏穩定的永久識別碼；
- 清理和解讀資料的程序繁冗且不相容；
- 缺少可以讓知識專家用來策畫並改善資料的功能機制。

由於意識到在各個規模的努力必須同步並進，全球生物多樣性機構 (GBIF) 於 2018 年 7 月舉辦第二屆全球生物多樣性資訊會議 (GBIC2)，提出一個發展生物多樣性資訊學共享路線圖的協調機制。經由全球基因組學和健康聯盟 (GA4GH) 和 Apache 軟體基金會開源軟體社群的案例分享—此些倡議為多個利益關係者提供分散基金和獨立管理的模式，以整合資源並發展永續的方案來解決共同需求—GBIC2 與會者對於建立一個生物多樣性知識全球聯盟的需求達成了共識。

提升合作機會、改善現有的資料管理及協同發展新的資料來源，可為生物多樣性在所有面向帶來一個整合、相互關聯的單一知識庫—可以自由、公開地傳播給有需要或有興趣使用它的每個人。實現這樣一個系統將使我們能夠把當前生物多樣性的科學知識融入到支持永續未來，合理、及時的決策過程中。

Supplementary Material 13: 呼籲成立生物多樣性知識聯盟
doi:10.3897/BDJ.7.e33679.suppl13

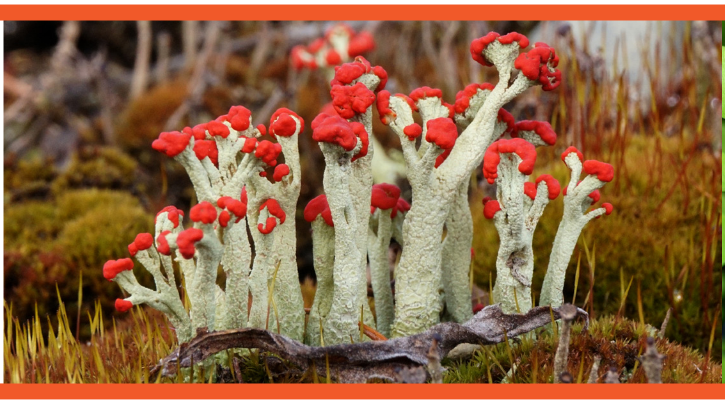

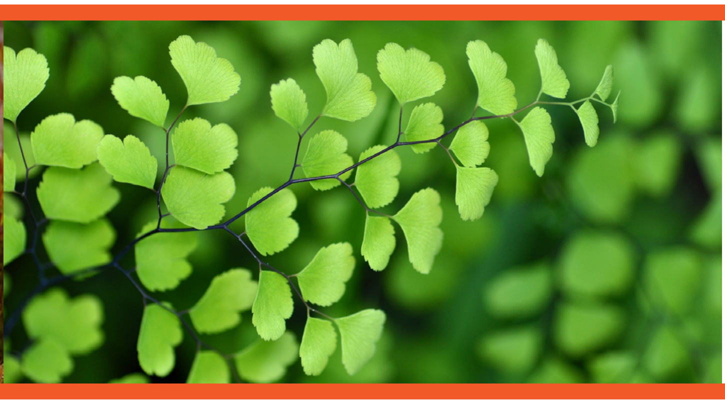

追求目標

GBIC2 的與會者提出以下多個面向的願景，以定**義生物多樣性知識聯盟**的理念：

支持科学和循证规划

1. 以支援關鍵研究需求及正確地測量和評估生物多樣性來實現社會目標的形式，提供生物多樣性的知識和理解。
2. 作為生物多樣性和資訊科學基礎研究的根基，使人類了解自然系統的功能和狀態。
3. 藉由保存、建構和改善現有知識，提供一個持續茁壯的生物多樣性知識平台。

支援開放資料與開放科學

4. 消除自由和開放共享資料的屏障，採用生物多樣性資料的FAIR資料原則（Wilkinsonetal.2016）。
5. 為各資料資源提供豐富的詮釋資料，以促進當前與未來的資料再使用。
6. 確保所有資料資源都被保存於穩定且持續可信的資料庫中。
7. 由相關專家和專家團體來協同策畫、註記和改善資料內容。
8. 確保所有知識或專長貢獻者的貢獻皆被充分記錄、認可與歸功。
9. 追蹤所有資訊來源的出處和歸屬。

支援高度關聯的生物多樣性資料

10. 流通歷史資料來源的結構化數位版本，包括博物館館藏與文獻。
11. 確保新的觀測記錄與測量能在擷取後儘快地以結構化的數位版本提供取用後。
12. 使不同類別的生物多樣性資訊（分布、特徵、基因等）能以一個相互連結的整體提供組合、查詢和分析。
13. 與其他研究社群和基礎設施合作，以實現與地球觀測、社會科學資料和其他資源的互通性。

Image credits. p.1: AuthaGraph map of the world with political borders adapted from **Justin Kunimune**, licensed under CC BY-SA 4.0. ***p.2:*** (*left*) British soldier lichen (*Cladonia cristatella*), by Mike Lewinski via **iNaturalist**, licensed under CC BY 2.0; (*right*) Black maidenhair fern (*Adiantum capillus-veneris*) by Robby Deans via **iNaturalist**, licensed under CC BY-NC 4.0.

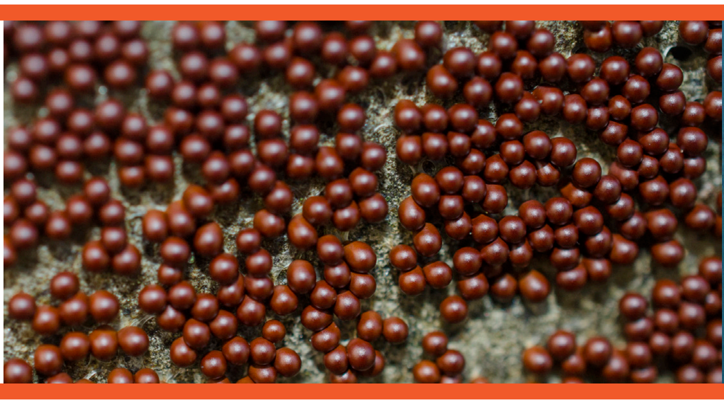 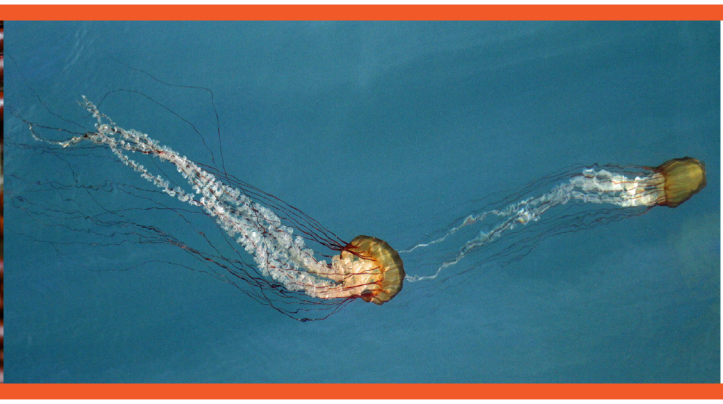

支援國際合作

14. 解決所有區域和部門的生物多樣性資訊學能力需求。
15. 獲取資金以維持社群認可為分散式知識基礎建設關鍵元素的服務和組成元件。
16. 發展彈性及協作的方法，以設計、建置和維持此一分散式知識基礎建設之所有組成元件。
17. 使每個國家和區域的利益關係者可以採用並獲益於基礎設施、工具、服務、做法及能量的進展。
18. 使所有區域的利益關係團體從資料產生、分析到應用的各階段皆能充分參與及合作。
19. 使所有國家及區域支援科學和政策制定的資料得以回歸國家及區域本身。
20. 使資料在各個尺度—全球、區域、國家、地方—均可有效率地取用。
21. 認可並支持區域、國家和地方的投資在全球解決方案中所扮演關鍵和具影響力的角色。
22. 克服在資料的共享和使用上的語言及文化隔閡。
23. 支持國際協定的實務履行，並強調取用及利益之共享。

下一步

我們敦促所有對生產、管理、使用和整合全球生物多樣性資料感興趣的利益關係者，藉由投入下列初步進程，貢獻於建立此研擬中的生物多樣性知識聯盟 。

更進一步的資訊，以及投入相關討論的機會，請參考聯盟**的網頁**。您可造訪網頁的討論選單，參與下面這五個單**元的討論**。**歡迎使用英語之外的任何語言**。

拓展參與

工作坊、工作報告、徵求活動等都已經為全球社群準備好了。我們鼓勵對生物多樣性資訊流通、改善、整合或使用有興趣的個人及機構，透過聯盟網站簽署和（或）訂閱進一步資訊來表達支持。

p.3: (*left*) Insect-egg mold (*Leocarpus fragilis*) by Tim Sage via **iNaturalist**, licensed under CC BY-NC 4.0; (*right*) Pacific sea nettle (*Chrysaora fuscescens*) by Robin Agarwal via **iNaturalist**, licensed under CC BY-NC 4.0.

評估模式

需要投入更多的工作來滿足複雜多元的利益關係社群之需求，但基於其他類似聯盟、結合與合夥關係的模式（例如許多開源軟體計畫使用以價值為基礎的「Apache Way」），可以指導與揭露長期的做法。重要的問題仍與會員（個人，機構或兩者）的形成基礎相關。

明定範圍及標的成果

毫無疑問地，加強合作將對生物多樣性資訊領域帶來顯著的益處和效率，但終極的目標是為科學、政策和社會帶來影響。GBIC2 與會者建議與不同的利益關係者—包括研究團體、物種分類機構、生物多樣性公約組織（CBD）、生物多樣性和生態系統服務政府間科學政策平台（IPBES）、聯合國糧農組織（FAO）、保育團體和其他使用者社群—發展一套定義問題與可實現的案例，以衡量進展情況。這些應足以精確、詳細地導引協作規劃、發展和實施之優先順序。

勘測利益關係者

由於活動的數量龐大，任務經常重疊，工作計畫的實施有不同的時間表及責任範圍，聯盟工作項目中利益關係者的全貌相當難以理解。除非已經理解此複雜性，否則將有無意的衝突或重工的巨大風險。

GBIF 將協調初步的網絡分析—一個具有嚴格和明確範圍限制的分析—設法概繪主要組織所扮演的角色、責任與關係，特別是在全球、區域和國家的尺度。 這項工作將有助於聯盟確定需要創立或維持的關鍵服務，並指出機會以為其達成更好地校準或統一。

採用概念驗證計畫

聯盟的關鍵目標即是讓利益相關者圍繞在共同需求之下匯集並醞釀永續的計畫，為貢獻及完成互聯的數位知識系統，提供工具、服務、模式和資源。我們需要有正式的流程來排定優先順序、培養、實現和維持這些計畫。短期內，確認當前支持開放聯盟願景的活動有其價值，可以將其採納為概念驗證計畫。這些計畫可以提供經驗，納入未來的理事模式，同時作為聯盟式合作的一個初期範例。我們不僅在軟體開發或資料管理方面，也在其他領域如能量倍增和永續規畫方面，尋求提議合適且進行中活動，以作為初期概念驗證計畫的候選者。

Hobern D, Baptiste B, Copas K, Guralnick R, Hahn A, van Huis E, Kim E-S, McGeoch M, Naicker I, Navarro L, Noesgaard D, Price M, Rodrigues A, Schigel D, Sheffield CA & Wieczorek J (2019) Connecting data and expertise: a new alliance for biodiversity knowledge. Biodiversity Data Journal. doi:10.3897/BDJ.7.e33679
